# Design, synthesis, biological evaluation and QSAR analysis of novel *N*-substituted benzimidazole derived carboxamides

**DOI:** 10.1080/14756366.2022.2070910

**Published:** 2022-05-06

**Authors:** Anja Beč, Marija Mioč, Branimir Bertoša, Marija Kos, Patricia Debogović, Marijeta Kralj, Kristina Starčević, Marijana Hranjec

**Affiliations:** a Department of Organic Chemistry, Faculty of Chemical Engineering and Technology, University of Zagreb, Zagreb, Croatia; b Division of Molecular Medicine, Ruđer Bošković Institute, Zagreb, Croatia; c Department of Chemistry, Faculty of Science, University of Zagreb, Zagreb, Croatia; d Department of Chemistry and Biochemistry, Faculty of Veterinary Medicine, University of Zagreb, Zagreb, Croatia

**Keywords:** Antioxidant activity, antiproliferative activity, benzimidazoles, carboxamides, QSAR, ROS

## Abstract

As a result of our previous research focussed on benzimidazoles, herein we present design, synthesis, QSAR analysis and biological activity of novel N-substituted benzimidazole derived carboxamides. Carboxamides were designed to study the influence of the number of methoxy groups, the type of the substituent placed at the benzimidazole core on biological activity. Pronounced antioxidative activity displayed unsubstituted **28** (IC_50_ ≈ 3.78 mM, 538.81 mmolFe^2+^/mmolC) and dimethoxy substituted derivative **34** (IC_50_ ≈ 5.68 mM, 618.10 mmolFe^2+^/mmolC). Trimethoxy substituted **43** and unsubstituted compound **40** with isobutyl side chain at N atom showed strong activity against HCT116 (IC_50_ ≈ 0.6 µM, both) and H 460 cells (IC_50_ ≈ 2.5 µM; 0.4 µM), being less cytotoxic towards non-tumour cell. Antioxidative activity in cell generally confirmed relatively modest antioxidant capacity obtained in DPPH/FRAP assays of derivatives **34** and **40**. The 3D-QSAR models were generated to explore molecular properties that have the highest influence on antioxidative activity.

## 1. Introduction

Heterocyclic compounds are indispensable building blocks in drug design and medicinal chemistry due to their structural tuneability and diverse pharmacological features^1^^,^^2^. Additionally, the structures of aromatic heterocycles are conformationally restricted which improve their selectivity and bioactivity in comparison to their acyclic analogues^3^. In the last few decades, benzimidazole derivatives, have attracted great interest of organic and medicinal chemists as an important multifunctional system for possible medicinal applications in various therapeutic fields^4^^,^^5^. Benzimidazole derivatives became important since the discovery of the vitamin B12 structure which confirmed that *N*-ribosyl-5,6-dimethylbenzimidazole serves as an axial ligand to cobalt^6^. Furthermore, the benzimidazole structural motif was identified in many biologically important molecules with an important role for the interaction with enzymes and different proteins^7^^,^^8^. The broad range of biological activities of benzimidazole derivatives includes primarily antitumor^9^, antiviral^10^, antimicrobial,^11^ anti-inflammantory^12^, antifungal^13^, antiprotozoal^14^, antihypertensive^15^, antioxidative^16^, etc. activities.

**Figure 1.**
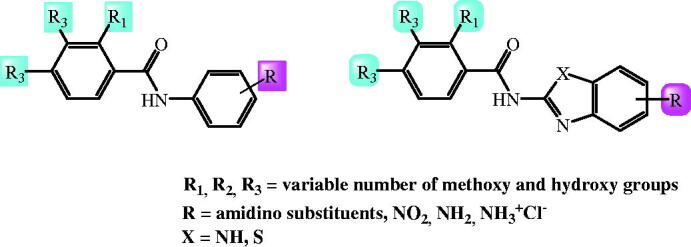
Recently published benzamides and benzimidazole-2-carboxamides with great antioxidative potential.

In our previous work, we described the design, synthesis, computational analysis and biological activity of multipurpose benzimidazole derivatives with significant antiproliferative, antioxidant and antimicrobial activities. We synthesised several classes of benzimidazole derivatives including amidino substituted derivatives^17^, styryl derivatives^18^^,^^19^, benzo[*b*]thienyl substituted derivatives^20^, tricyclic^21^, tetracyclic^22^ and pentacyclic^23^ derivatives substituted with amidino, amino and amido side chains, 2-carboxamides bearing amidino, nitro and amino substituents,^24^ acrylonitriles^25^ and Schiff bases^26^. Taking into account very promising and significant biological activities displayed by recently synthesised benzimidazole derivatives, our research has been focussed on the design and optimisation of novel benzimidazoles as potential antioxidants with antitumor activity. Among all synthesised derivatives, we demonstrated a great antioxidative potential of benzamides^27^ and benzimidazole/benzothiazole-2-carboxamides^16^^,^^27^ bearing variable number of hydroxyl or methoxy group, substituted with amidino, cyano, nitro and/or amino substituents placed at the phenyl or heteroaromatic scaffold (Figure 1). Importantly, we noticed that the type and the number of the substituents have shown a strong impact on the biological activity.

Within this manuscript we presented the design, synthesis, QSAR analysis and biological activity of novel *N*-substituted benzimidazole-2-carboxamides bearing different number of methoxy groups on the phenyl ring. Additionally, our intention was to study the influence of the substituent placed at the N atom of benzimidazole core on the biological activity since it is well known from the literature data that the most important positions for the substitution on benzimidazole core are N-1, C-2 and C-5(6). Newly prepared compounds were tested for their antioxidative and antiproliferative activity, while the 3D-QSAR analysis allowed the correlation of the chemico-physical properties with biological activity. Additionally, it is a well-known fact that oxidative stress is connected to promotion of many aspects of tumour development and progression^28^^,^^29^. At molecule level the key signalling protein, nuclear factor erythroid 2-related factor 2 (Nrf2) is considered to be the master regulator of the antioxidant response^30^^,^^31^. Therefore, we also investigated whether newly prepared compounds exert antioxidative activity by activating Nrf2 pathway.

## 2. Experimental part

### 2.1. Chemistry

#### 2.1.1. General methods

All chemicals and solvents were purchased from commercial suppliers Aldrich and Acros. Melting points were recorded on SMP11 Bibby and Büchi 535 apparatus. ^1^H and ^13^C NMR spectra were recorded on a Varian Gemini 300 or Varian Gemini 600 spectrophotometer at 300, 600, 150, and 75 MHz, respectively. All NMR spectra were measured in DMSO-d_6_ solutions using TMS as an internal standard.

Chemical shifts are reported in ppm (δ) relative to TMS. All compounds were routinely checked by thin layer chromatography (TLC) using precoated Merck silica gel 60 F-254 plates and the spots were detected under UV light (254 nm). Microwave-assisted synthesis was performed in a Milestone start S microwave oven using quartz cuvettes under the pressure of 40 bar. Elemental analysis for C, H and N were performed on a Perkin-Elmer 2400 elemental analyser. Where analyses are indicated only as symbols of elements, analytical results obtained are within 0.4% of the theoretical value.

#### 2.1.2. General method for preparation of compounds 3–6

Compounds **3**–**6** were prepared using microwave irradiation, at optimised reaction time at 170 °C with power 800 W and 40 bar pressure, from **1** or **2** in acetonitrile (10 ml) with excess of added corresponding amine. After cooling, resulting product was purified by column chromatography on SiO_2_ using dichlormethane/methanol as eluent.

*N-Isobutyl-2-nitroaniline* ***3***. Compound **3** was prepared from 1 (0.50 g, 3.2 mmol) and isobutylamine (1.60 ml, 15.9 mmol) after 3 h of irradiation to yield 0.60 g (94%) of orange oil. ^1^H NMR (300 MHz, DMSO-d_6_): δ/ppm = 8.19 (bs, 1H, NH), 8.07 (dd, 1H, J_1_ = 8.6 Hz, J_2_ = 1.6 Hz, H_arom_), 7.52 (td, 1H, J_1_ = 8.4 Hz, J_2_ = 1.2 Hz, H_arom_), 7.06 (d, 1H, J = 8.5 Hz, H_arom_), 6.68 (td, 1H, J_1_ = 8.3 Hz, J_2_ = 1.1 Hz, H_arom_), 3.19 (t, 2H, J = 6.3 Hz, CH_2_), 2.01‒1.90 (m, 1H, CH), 0.96 (d, 6H, J = 6.7 Hz, CH_3_); ^13^C NMR (75 MHz, DMSO-d_6_): δ/ppm = 145.9, 137.1, 131.2, 126.7, 115.6, 115.1, 50.0, 27.7, 20.5 (2 C); Anal. Calcd. for C_10_H_14_N_2_O_2_: C, 61.84; H, 7.27; N, 14.42. Found: C, 61.64; H, 7.35; N, 14.20%.

*3-N-(Methylamino)-4-nitrobenzonitrile* ***4***. Compound **4** was prepared from **2** (0.50 g, 2.7 mmol) and methylamine (2.60 ml, 58.5 mmol) after 2 h of irradiation to yield 0.50 g (74%) of yellow powder. m.p. 174‒178 °C; ^1^H NMR (300 MHz, DMSO-d_6_): δ/ppm = 8.64 (q, 1H, *J* = 5.5 Hz, NH), 8.49 (d, 1H, *J* = 2.0 Hz, H_arom_), 7.84 (dd, 1H, *J*_1_ = 9.1 Hz, *J*_2_ = 1.6 Hz, H_arom_), 7.10 (d, 1H, *J* = 9.1 Hz, H_arom_), 3,00 (d, 3H, *J* = 5.0 Hz, CH_3_); ^13^C NMR (75 MHz, DMSO-d_6_): δ/ppm = 148.0, 138.1, 132.2, 131.2, 118.8, 116.1, 96.5, 30.3; Anal. Calcd. for C_8_H_7_N_3_O_2_: C, 54.24; H, 3.98; N, 23.72. Found: C, 54.04; H, 3.80; N, 23.95%.

*3-N-(Isobutylamino)-4-nitrobenzonitrile* ***5.*** Compound **5** was prepared from **2** (0.50 g, 2.7 mmol) and isobutylamine (1.90 ml, 19.2 mmol) after 2 h of irradiation to yield 0.55 g (73%) of yellow powder. m.p. 99–101 °C; ^1^H NMR (300 MHz, DMSO-d_6_): δ/ppm = 8.62 (t, 1H, *J* = 5.5 Hz, NH), 8.51 (d, 1H, *J* = 2.0 Hz, H_arom_), 7.81 (dd, 1H, *J*_1_ = 9.0 Hz, *J*_2_ = 1.8 Hz, H_arom_), 7.21 (d, 1H, *J* = 9.2 Hz, H_arom_), 3.27 (t, 2H, *J* = 6.5 Hz, CH_2_), 2.03‒1.86 (m, 1H, CH), 0.95 (d, 6H, *J* = 6.7 Hz, CH_3_); ^13^C NMR (151 MHz, DMSO-d_6_): δ/ppm = 147.0, 137.5, 131.9, 130.7, 118.2, 115.9, 96.2, 49.5, 27.2, 19.8 (2 C); Anal. Calcd. for C_11_H_13_N_3_O_2_: C, 60.26; H, 5.98; N, 19.17. Found: C, 60.39; H, 5.80; N, 19.30%.

*4-Nitro-3-N-(phenylamino)benzonitrile* ***6***. Compound **6** was prepared from **2** (0.50 g, 2.7 mmol) and aniline (1.0 ml, 10.0 mmol) after 2 h of irradiation to yield 0.32 g (48%) of orange powder. m.p. 131–135 °C; ^1^H NMR (300 MHz, DMSO-d_6_): δ/ppm = 9.90 (s, 1H, NH), 8.59 (d, 1H, *J* = 2.0 Hz, H_arom_), 7.77 (dd, 1H, *J*_1_ = 9.0 Hz, *J*_2_ = 1.9 Hz, H_arom_), 7.51‒7.46 (m, 2H, H_arom_), 7.37‒7.30 (m, 3H, H_arom_), 7.09 (d, 1H, *J* = 9.0 Hz, H_arom_); ^13^C NMR (75 MHz, DMSO-d_6_): δ/ppm = 145.6, 138.3, 138.0, 132.9, 132.3, 130.2 (2 C), 127.0, 125.9 (2 C), 118.4, 117.7, 99.0; Anal. Calcd. for C_13_H_9_N_3_O_2_: C, 65.27; H, 3.79; N, 17.56. Found: C, 65.39; H, 3.99; N, 17.30%.

#### 2.1.3. General method for preparation of compounds 7–10

Derivative **3** or benzonitrile derivatives **4**–**6** and a solution of SnCl_2 _×2H_2_O in MeOH and concentrated HCl were refluxed for 0.5 h. The resulting solution was treated with 20% NaOH to pH = 14. The resulting precipitate was filtered off, washed with hot ethanol and filtered. The filtrate was evaporated at a reduced pressure and extracted with ethyl acetate. The organic layer was dried over anhydrous MgSO_4_ and concentrated at reduced pressure.

*N-Isobutylbenzene-1,2-diamine* ***7***. Compound **7** was prepared from **3** (2.35 g, 12.1 mmol), SnCl_2 _×2H_2_O (16.40 g, 72.7 mmol), HCl_conc._ (32 ml) and MeOH (32 ml) to obtain a orange oil (1.10 g, 55%). ^1^H NMR (300 MHz, DMSO-d_6_): δ/ppm = 6.53 (dd, 1H, *J*_1_ = 7.7 Hz, *J*_2_ = 1.6 Hz, H_arom_), 6.47 (dd, 1H, *J*_1_ = 7.1 Hz, *J*_2_ = 2.0 Hz, H_arom_), 6.43‒6.34 (m, 2H, H_arom_), 4.48 (s, 2H, NH_2_), 4.33 (bs, 1H, NH), 2.82 (t, 2H, *J* = 6.0 Hz, CH_2_), 1.93‒1.80 (m, 1H, CH), 0.95 (d, 6H, *J* = 6.6 Hz, CH_3_); Anal. Calcd. for C_10_H_16_N_2_: C, 73.13; H, 9.82; N, 17.06. Found: C, 73.25; H, 9.89; N, 16.86%.

*4-Amino-3-(methylamino)benzonitrile* ***8***. Compound **8** was prepared from **4** (2.51 g, 14.2 mmol), SnCl_2_ × 2H_2_O (26.59 g, 117.9 mmol), HCl_conc._ (39 ml) and H_2_O (39 ml) to obtain a light orange powder (1.01 g, 48%). m.p. 149–151 °C; ^1^H NMR (300 MHz, DMSO-d_6_): δ/ppm = 6.95 (dd, 1H, *J*_1_ = 8.1 Hz, *J*_2_ *=* 1.9 Hz, H_arom_), 6.77 (d, 1H, *J* = 2.0 Hz, H_arom_), 6.41 (d, 1H, *J* = 8.2 Hz, H_arom_), 5.57 (q, 1H, *J* = 4.7 Hz, NH), 4.89 (s, 2H, NH_2_), 2.77 (d, 3H, *J* = 4.8 Hz, CH_3_); ^13^C NMR (75 MHz, DMSO-d_6_): δ/ppm = 141.6, 135.6, 123.5, 121.6, 114.9, 108.5, 96.9, 29.9; Anal. Calcd. for C_18_H_9_N_3_: C, 65.29; H, 6.16; N, 28.55. Found: C, 65.10; H, 6.06; N, 24.84%.

*4-Amino-3-(isobutylamino)benzonitrile* ***9***. Compound **9** was prepared from **5** (2.20 g, 10.0 mmol), SnCl_2 _× 2H_2_O (18.10 g, 80.2 mmol), HCl_conc._ (26 ml) and H_2_O (26 ml) to obtain a white powder (1.05 g, 55%). m.p. 120–125 °C; ^1^H NMR (300 MHz, DMSO-d_6_): δ/ppm = 6.89 (dd, 1H, *J*_1_ = 8.2 Hz, *J*_2_ = 1.9 Hz, H_arom_), 6.76 (d, 1H, *J* = 2.0 Hz, H_arom_), 6.43 (d, 1H, *J* = 8.3 Hz, H_arom_), 5.44 (bs, 1H, NH), 5.03 (s, 2H, NH_2_), 2.91 (t, 2H, *J* = 6.2 Hz, CH_2_), 1.95‒1.80 (m, 1H, CH), 0.94 (d, 6H, *J* = 6.6 Hz, CH_3_); ^13^C NMR (75 MHz, DMSO-d_6_): δ/ppm = 140.5, 135.5, 123.2, 121.6, 115.1, 108.9, 96.6, 51.0, 27.4, 20.9 (2 C); Anal. Calcd. for C_11_H_15_N_3_: C, 69.81; H, 7.99; N, 22.20. Found: C, 69.95; H, 7.80; N, 22.25%.

*4-Amino-3-(phenylamino)benzonitrile* ***10***. Compound **10** was prepared from **6** (1.58 g, 6.6 mmol), SnCl_2 _×2H_2_O (12.39 g, 54.9 mmol), HCl_conc._ (18 ml) and H_2_O (18 ml) to obtain a light yellow powder (1.12 g, 81%). m.p. 150–153 °C; ^1^H NMR (300 MHz, DMSO-d_6_): δ/ppm = 7.52 (s, 1H, NH), 7.29‒7.24 (m, 2H, H_arom_), 7.09 (d, 1H, *J* = 8.2 Hz, H_arom_), 7.03 (d, 2H, *J* = 8.5 Hz, H_arom_), 7.00 (d, 1H, *J* = 1.9 Hz, H_arom_), 6.94‒6.87 (m, 2H, H_arom_), 5.25 (s, 2H, NH_2_); ^13^C NMR (75 MHz, DMSO-d_6_): δ/ppm = 142.9, 139.7, 134.7, 129.7 (2 C), 121.6, 121.3, 120.6, 118.8 (2 C), 117.3, 116.8, 102.5; Anal. Calcd. for C_13_H_11_N_3_: C, 74.62; H, 5.30; N, 20.08. Found: C, 74.70; H, 5.45; N, 19.85%.

#### 2.1.4. General method for preparation of compounds 13–14

BrCN was added dropwise to a solution of *o*-phenylenediamine **7** or **11** in 20 ml H_2_O and 5 ml acetonitrile. The reaction mixture was refluxed for 2 h and NH_4_OH was added to adjust to pH = 9. After cooling, the resulting precipitate was filtered off.

*2-Amino-1-methylbenzimidazole* ***13.*** Compound **13** was prepared from **11** (0.70 g, 5.7 mmol) and BrCN (0.61 g, 5.7 mmol) to obtain a brown powder (0.65 g, 77%). m.p. 159‒162 °C; ^1^H NMR (400 MHz, DMSO-d_6_): δ/ppm = 7.14‒7.09 (m, 2H, H_arom_), 6.94 (td, 1H, *J*_1_ = 7.5 Hz, *J*_2_ = 1.4 Hz, H_arom_), 6.89 (td, 1H, *J*_1_ = 7.4 Hz, *J*_2_ = 1.3 Hz, H_arom_), 6.40 (s, 2H, NH_2_), 3.49 (s, 3H, CH_3_); ^13^C NMR (75 MHz, DMSO-d_6_): δ/ppm = 150.7, 131.5, 129.2, 123.9, 123.4, 111.9, 110.6, 29.6; Anal. Calcd. for C_8_H_9_N_3_: C, 65.29; H, 6.16; N, 28.55. Found: C, 65.33; H, 6.22; N, 28.59%.

*2-Amino-1-isobutylbenzimidazole* ***14***. Compound **14** was prepared from **7** (1.10 g, 6.7 mmol) and BrCN (0.71 g, 6.7 mmol) to obtain a light orange powder (0.68 g, 54%). m.p. 121‒125 °C; 1H NMR (300 MHz, DMSO-d_6_): δ/ppm = 8.43 (s, 2H, NH2), 7.54–7.48 (m, 1H, H_arom_), 7.38–7.33 (m, 1H, H_arom_), 7.25–7.19 (m, 2H, H_arom_), 3.94 (d, 2H, *J* = 7.7 Hz, CH_2_), 2.18–2.07 (m, 1H, CH), 0.89 (d, 6H, *J* = 6.6 Hz, CH_3_); ^13^C NMR (151 MHz, DMSO-d_6_): δ/ppm = 150.8, 131.3, 123.0, 122.1, 121.7, 112.0, 110.2, 48.6, 27.3, 19.3 (2 C); Anal. Calcd. for C_11_H_15_N_3_: C, 69.81; H, 7.99; N, 22.20. Found: C, 69.77; H, 7.89; N, 22.15%.

#### 2.1.5. General method for preparation of compounds 15, 16–18

To a solution of *N*-phenyl-o-phenylenediamine **12** or benzonitrile derivatives **8**–**10** and H_2_O/MeOH, solution of BrCN in acetonitrile was added. The reaction mixture was stirred at room temperature for 24 h. NH_4_OH was added to adjust to pH = 9. After cooling, the resulting precipitate was filtered off purified by column chromatography on SiO_2_ using dichlormethane/methanol as eluent.

*2-Amino-1-phenylbenzimidazole* ***15***. Compound **15** was prepared from **12** (1.00 g, 5.4 mmol), H_2_O/MeOH (20 ml), BrCN (0.57 g, 5.4 mmol) in acetonitrile (1 ml) to obtain a light pink powder (0.57 g, 51%). m.p. 149‒152 °C; ^1^H NMR (400 MHz, DMSO-d_6_): δ/ppm = 7.66‒7.60 (m, 2H, H_arom_), 7.54 (d, 1H, *J* = 7.5 Hz, H_arom_), 7.50 (dd, 2H, *J*_1_ = 8.4 Hz, *J*_2_ = 1.2 Hz, H_arom_), 7.25 (d, 1H, *J* = 7.7 Hz, H_arom_), 7.04 (td, 1H, *J*_1_ = 7.8 Hz, *J*_2_ = 1.5 Hz, H_arom_), 6.90 (td, 1H, *J*_1_ = 7.5 Hz, *J*_2_ = 1.1 Hz, H_arom_), 6.86 (dd, 1H, *J*_1_ = 7.8 Hz, *J*_2_ = 0.9 Hz, H_arom_), 6.44 (s, 2H, NH_2_); ^13^C NMR (101 MHz, DMSO-d_6_): δ/ppm = 154.3, 142.3, 135.2, 135.1, 130.6 (2 C), 128.8, 127.2 (2 C), 121.9, 119.6, 115.3, 108.3; Anal. Calcd. for C_13_H_11_N_3_: C, 74.62; H, 5.30; N, 20.08. Found: C, 74.58; H, 5.37; N, 20.04%.

*2-Amino-6-cyano-1-methylbenzimidazole* ***16***. Compound **16** was prepared from **8** (1.01 g, 6.9 mmol), H_2_O/MeOH (15 ml), BrCN (0.73 g, 6.9 mmol) in acetonitrile (1.6 ml) to obtain a white powder (0.57 g, 48%). m.p. 276‒280 °C; ^1^H NMR (400 MHz, DMSO-d_6_): δ/ppm = 7.51‒7.49 (m, 1H, H_arom_), 7.31‒7.30 (m, 1H, H_arom_), 7.30 (s, 1H, H_arom_), 6.83 (s, 2H, NH_2_), 3.55 (s, 3H, CH_3_); ^13^C NMR (101 MHz, DMSO-d_6_): δ/ppm = 157.7, 143.3, 138.9, 122.9, 121.2, 118.2, 108.8, 102.5, 29.1; Anal. Calcd. for C_9_H_8_N_4_: C, 62.78; H, 4.68; N, 32.54. Found: C, 62.71; H, 4.61; N, 32.59%.

*2-Amino-6-cyano-1-isobutylbenzimidazole* ***17***. Compound **17** was prepared from **9** (1.05 g, 5.6 mmol), H_2_O/MeOH (20 ml), BrCN (0.59 g, 5.6 mmol) in acetonitrile (1 ml) to obtain a grey powder (0.55 g, 47%). m.p. 262‒266 °C; ^1^H NMR (400 MHz, DMSO-d_6_): δ/ppm = 7.50‒7.48 (m, 1H, H_arom_), 7.34 (d, 1H, *J* = 8.0 Hz, H_arom_), 7.28 (dd, 1H, *J*_1_ = 7.9 Hz, *J*_2_ = 1.3 Hz, H_arom_), 6.82 (s, 2H, NH_2_), 3.85 (d, 2H, *J* = 7.6 Hz, CH_2_), 2.13‒2.06 (m, 1H, CH), 0.85 (d, 6H, *J* = 6.7 Hz, CH_3_); ^13^C NMR (101 MHz, DMSO-d_6_): δ/ppm = 157.5, 143.3, 138.7, 122.7, 121.2, 118.2, 109.3, 102.4, 48.9, 28.2, 19.9 (2 C); Anal. Calcd. for C_12_H_14_N_4_: C, 67.27; H, 6.59; N, 26.15. Found: C, 67.24; H, 6.61; N, 26.22%.

*2-Amino-6-cyano-1-phenylbenzimidazole* ***18***. Compound **18** was prepared from **10** (1.12 g, 5.3 mmol), H_2_O/MeOH (25 ml), BrCN (0.57 g, 5.3 mmol) in acetonitrile (1 ml) to obtain a white powder (0.40 g, 16%). m.p. > 300 °C; ^1^H NMR (400 MHz, DMSO-d_6_): δ/ppm = 7.70‒7.60 (m, 3H, H_arom_), 7.59‒7.54 (m, 1H, H_arom_), 7.52‒7.48 (m, 2H, H_arom_), 7.28 (dd, 1H, *J*_1_ = 8.2 Hz, *J*_2_ = 1.5 Hz, H_arom_), 6.93 (d, 1H, *J* = 8.2 Hz, H_arom_), 6.70 (s, 2H, NH_2_); ^13^C NMR (101 MHz, DMSO-d_6_): δ/ppm = 156.7, 143.7, 138.9, 134.4, 130.7 (2 C), 129.4, 127.4 (2 C), 123.6, 120.9, 118.7, 108.9, 103.5; Anal. Calcd. for C_14_H_10_N_4_: C, 71.78; H, 4.30; N, 23.92. Found: C, 71.71; H, 4.36; N, 23.89%.

#### 2.1.6. General method for preparation of carboxamides 24‒51

To a solution of mono-, di-, or trimethoxy substituted benzoylchloride in dry toluene, corresponding amine was added followed by the addition of Et_3_N. The reaction mixture was refluxed for 24 h. After cooling, the resulting product was filtered off, washed with diluted HCl and water to obtain pure solid, and recrystallized from a proper solvent if needed.

*N-(1H-Benzo[d]imidazol-2-yl)benzamide* ***24***. From 0.10 g (0.8 mmol) of benzoylchloride **23**, 0,10 g (0.8 mmol) of 2-aminobenzimidazole **19** and 0.09 g (0.9 mmol) of Et_3_N in dry toluene (15 ml), 0.15 g (84%) of light yellow powder was obtained. m.p. 234–238 °C; ^1^H NMR (600 MHz, DMSO-d_6_): δ/ppm = 12.62 (s, 1H, NH_benz_) 8.16 (d, 2H, *J* = 7.3 Hz, H_arom_), 7.66–7.62 (m, 1H, *J* = 7.3 Hz, H_arom_), 7.58–7.54 (m, 2H, H_arom_), 7.54–7.52 (m, 2H, H_arom_), 7.23–7.19 (m, 2H, H_arom_); ^13^C NMR (151 MHz, DMSO-d_6_): δ/ppm = 167.8, 147.8, 133.7 (2 C), 133.1, 132.4 (2 C), 128.4 (2 C), 128.3 (2 C), 122.2 (2 C), 113.4; Anal. Calcd. for C_18_H_15_N_3_O: C, 70.87; H, 4.67; N, 17.71. Found: C, 70.84; H, 4.73; N, 17.74%.

*N-(1H-Benzo[d]imidazol-2-yl)-2-methoxybenzamide* ***25***. From 0.13 g (0.8 mmol) of 2-methoxybenzoylchloride **20**, 0.10 g (0.8 mmol) of 2-aminobenzimidazole **19** and 0.15 g (0.2 mmol) of Et_3_N in dry toluene (15 ml), 0.10 g (48%) of yellow powder was obtained. m.p. 224–227 °C; ^1^H NMR (600 MHz, DMSO-d_6_): δ/ppm = 12.13 (s, 1H, NH_benz_), 7.88 (dd, 1H, *J*_1_ = 7.7, *J*_2_ = 1.7 Hz, H_arom._), 7.64 (td, 1H, *J*_1_ = 7.0 Hz, *J*_2_ = 1.7 Hz, H_arom_), 7.59–7.55 (m, 2H, H_arom_), 7.28 (d, 1H, *J* = 8.4 Hz, H_arom_), 7.25–7.23 (m, 2H, H_arom_), 7.16 (t, 1H, *J* = 7.5 Hz, H_arom_), 4.02 (s, 3H, CH_3_); ^13^C NMR (151 MHz, DMSO-d_6_): δ/ppm = 167.8, 147.8, 133.7 (2 C), 133.1, 132.4 (2 C), 128.0, 129.3 (2 C), 124.2 (2 C), 113.4, 61.4; Anal. Calcd. for C_18_H_15_N_3_O: C, 67.40; H, 4.90; N, 15.72. Found: C, 67.45; H, 4.86; N, 15.74%.

*N-(1H-Benzo[d]imidazol-2-yl)-2,4-dimethoxybenzamide* ***26***. From 0.15 g (0.8 mmol) of 2,4-dimethoxybenzoylchloride **21**, 0.10 g (0.75 mmol) of 2-aminobenzimidazole **19** and 0.09 g (0.9 mmol) of Et_3_N in dry toluene (15 ml), 0.08 g (38%) of yellow powder was obtained. m.p. 249–253 °C; ^1^H NMR (600 MHz, DMSO-d_6_): δ/ppm = 11.53 (s, 1H, NH_benz_), 8.00 (d, 1H, *J* = 8.7 Hz, H_arom._), 7.65–7.62 (m, 2H, H_arom_), 7.35–7.32 (m, 2H, H_arom_), 6.80 (s, 1H, H_arom_), 6.79 (d, *J* = 8.8 Hz, 1H, H_arom_), 4.12 (s, 3H, CH_3_), 3.90 (s, 3H, CH_3_); ^13^C NMR (151 MHz, DMSO-d_6_): δ/ppm = 165.0 (2 C), 163.1 (2 C), 159.6, 144.3, 133.2, 123.4, 113.6, 111.7, 107.0 (2 C), 98.7 (2 C), 56.7, 55.9; Anal. Calcd. for C_18_H_15_N_3_O: C, 64.64; H, 5.09; N, 14.13. Found: C, 64.69; H, 5.04; N, 14.19%.

*N-(1H-Benzo[d]imidazol-2-yl)-3,4,5-trimethoxybenzamide* ***27***. From 0.15 g (0.8 mmol) of 3,4,5-trimethoxybenzoylchloride **22**, 0.10 g (0.8 mmol) of 2-aminobenzimidazole **19** and 0.09 g (0.9 mmol) of Et_3_N in dry toluene (15 ml), 0.20 g (80%) of light pink powder was obtained. m.p. 276–280 °C; ^1^H NMR (400 MHz, DMSO-d_6_): δ/ppm = 7.75–7.71 (m, 2H, H_arom_), 7.60 (s, 2H, H_arom_), 7.42–7.38 (m, 2H, H_arom_), 3.93 (s, 6H, CH_3_), 3.79 (s, 3H, CH_3_); ^13 ^C NMR (101 MHz, DMSO-d_6_): δ/ppm = 165.8, 153.3 (3 C), 145.1, 142.4, 130.1, 126.9, 124.7 (2 C), 114.1 (2 C), 106.8 (2 C), 60.7, 56.8 (2 C); Anal. Calcd. for C_18_H_15_N_3_O: C, 62.38; H, 5.23; N, 12.84. Found: C, 62.42; H, 5.24; N, 12.79%.

*N-(1-Methyl-1H-benzo[d]imidazol-2-yl)benzamide* ***28***. From 0.09 g (0.8 mmol) of benzoylchloride **23**, 0.10 g (0.8 mmol) of 2-amino-1-methylbenzimidazole **13** and 0.08 g (0.8 mmol) of Et_3_N in dry toluene (15 ml), 0.15 g (84%) of light pink powder was obtained. m.p. 243‒245 °C; ^1^H NMR (400 MHz, DMSO-d_6_): δ/ppm = 8.22 (dd, 2H, *J*_1_ = 7.0 Hz, *J*_2_ = 1.5 Hz, H_arom_), 7.62–7.55 (m, 3H, H_arom_), 7.54–7.50 (m, 2H, H_arom_), 7.35 (td, 1H, *J*_1_ = 6.5 Hz, *J*_2_ = 1.2 Hz, H_arom_), 7.31 (td, 1H, *J*_1_ = 7.5 Hz, *J*_2_ = 1.0 Hz, H_arom_), 3.80 (s, 3H, CH_3_); ^13^C NMR (101 MHz, DMSO-d_6_): δ/ppm = 133.3, 132.6, 130.5, 129.7 (2 C), 129.4, 129.0 (2 C), 128.7, 124.3, 124.4, 113.3, 111.0, 45.9; Anal. Calcd. for C_18_H_15_N_3_O: C, 71.70; H, 5.21; N, 16.72. Found: C, 71.69; H, 5.24; N, 16.77%.

*2-Methoxy-N-(1-methyl-1H-benzo[d]imidazol-2-yl)benzamide* ***29***. From 0.12 g (0.7 mmol) of 2-methoxybenzoylchloride **20**, 0.10 g (0.7 mmol) of 2-amino-1-methylbenzimidazole **13** and 0.08 g (0.8 mmol) of Et_3_N in dry toluene (15 ml), 0.01 g (7%) of light pink powder was obtained. m.p. 147–150 °C; ^1^H NMR (400 MHz, DMSO-d_6_): δ/ppm = 7.85 (td, 2H, *J*_1_ = 7.8 Hz, *J*_2_ = 1.6 Hz, H_arom_), 7.76 (dd, 1H, *J*_1_ = 6.4 Hz, *J*_2_ = 1.6 Hz, H_arom_), 7.70 (td, 1H, *J*_1_ = 8.8 Hz, *J*_2_ = 1.8 Hz, H_arom_), 7.54 (td, 1H, *J*_1_ = 6.7 Hz, *J*_2_ = 1.4 Hz, H_arom_), 7.50 (td, 1H, *J*_1_ = 6.6 Hz, *J*_2_ = 1.3 Hz, H_arom_), 7.33 (d, 1H, *J* = 8.3 Hz, H_arom_), 7.20 (td, 1H, *J*_1_ = 7.6 Hz, *J*_2_ = 0.8 Hz, H_arom_), 4.00 (s, 3H, CH_3_), 3.92 (s, 3H, CH_3_); ^13^C NMR (101 MHz, DMSO-d_6_): δ/ppm = 166.0, 158.0, 143.6, 135.0, 131.1, 130.5, 129.5, 125.6, 125.4, 121.7, 121.4, 114.5, 113.1, 112.2, 56.8, 31.0; Anal. Calcd. for C_18_H_15_N_3_O: C, 68.31; H, 5.37; N, 14.94. Found: C, 68.29; H, 5.44; N, 14.89%.

*2,4-Dimethoxy-N-(1-methyl-1H-benzo[d]imidazol-2-il)benzamide* ***30***. From 0.14 g (0.7 mmol) of 2,4-dimethoxybenzoylchloride **21**, 0.10 g (0.7 mmol) of 2-amino-1-methylbenzimidazole **13** and 0.08 g (0.8 mmol) of Et_3_N in dry toluene (15 ml), 0.08 g (38%) of white powder was obtained. m.p. 203–208 °C; ^1^H NMR (400 MHz, DMSO-d_6_): δ/ppm = 7.94 (d, 1H, *J* = 8.7 Hz, H_arom_), 7.88 (dd, 1H, *J*_1_ = 6.7 Hz, *J*_2_ = 1.4 Hz, H_arom_), 7.76 (dd, 1H, *J*_1_ = 6.4 Hz, *J*_2_ = 1.5 Hz, H_arom_), 7.53 (td, 1H, *J*_1_ = 6.5 Hz, *J*_2_ = 1.3 Hz, H_arom_), 7.50 (td, 1H, *J*_1_ = 6.5 Hz, *J*_2_ = 1.3 Hz, H_arom_), 6.85–6.83 (m, 1H, H_arom_), 6.80 (dd, 1H, *J*_1_ = 8.7 Hz, J_2_ = 2.3 Hz, H_arom_), 4.07 (s, 3H, CH_3_), 3.93 (s, 3H, CH_3_), 3.92 (s, 3H, CH_3_); ^13^C NMR (101 MHz, DMSO-d_6_): δ/ppm = 165.6, 164.2, 160.2, 143.5, 133.6, 130.5, 129.6, 125.6, 125.3, 114.6, 112.8, 112.1, 107.4, 99.4, 57.3, 56.4, 30.8; Anal. Calcd. for C_18_H_15_N_3_O: C, 65.58; H, 5.50; N, 13.50. Found: C, 65.54; H, 5.54; N, 5.46%.

*3,4,5-Trimethoxy-N-(1-methyl-1H-benzo[d]imidazol-2-yl)benzamide* ***31***. From 0.16 g (0.7 mmol) of 3,4,5-trimethoxybenzoylchloride **22**, 0.10 g (0.7 mmol) of 2-amino-1-methylbenzimidazole **13** and 0.08 g (0.8 mmol) of Et_3_N in dry toluene (10 ml), 0.18 g (77%) of purple powder was obtained. m.p. 232–236 °C; ^1^H NMR (400 MHz, DMSO-d_6_): δ/ppm = 7.70–7.67 (m, 2H, H_arom_), 7.56 (s, 2H, H_arom_), 7.45– 7.37 (m, 2H, H_arom_), 3.90 (s, 9H, CH_3_), 3.77 (s, 3H, CH_3_); ^13^C NMR (101 MHz, DMSO-d_6_): δ/ppm = 153.0 (3 C), 141.6, 130.7 (2 C), 129.4, 124.6, 124.4, 113.5, 111.3, 107.0 (2 C), 60.7 (2 C), 56.6 (2 C), 30.5; Anal. Calcd. for C_18_H_15_N_3_O: C, 63.33; H, 5.61; N, 12.31. Found: C, 63.28; H, 5.63; N, 12.36%.

*N-(6-Cyano-1-methyl-1H-benzo[d]imidazol-2-yl)benzamide* ***32***. From 0.08 g (0.6 mmol) of benzoylchloride **23**, 0.10 g (0.6 mmol) of 2-amino-5-cyano-1-methylbenzimidazole **16** and 0.07 g (0.7 mmol) of Et_3_N in dry toluene (15 ml), 0.10 g (62%) of light grey powder was obtained. m.p. 258–262 °C; ^1^H NMR (600 MHz, DMSO-d_6_): δ/ppm = 12.94 (s, 1H, NH_amide_), 8.27 (d, 2H, *J* = 7.2 Hz, H_arom_), 7.81 (s, 1H, H_arom_), 7.71 (d, 2H, *J* = 7.8 Hz, H_arom_), 7.66 (d, 1H, *J* = 8.2 Hz, H_arom_), 7.56–7.46 (m, 3H, H_arom_), 3.33 (s, 3H, CH_3_); ^13^C NMR (151 MHz, DMSO-d_6_): δ/ppm = 153.3, 144.1, 137.6, 131.4, 128.9 (2 C), 128.0 (2 C), 127.0, 119.4 (2 C), 115.1, 110.6, 104.1 (2 C), 28.6; Anal. Calcd. for C_18_H_15_N_3_O: C, 69.55; H, 4.38; N, 20.28. Found: C, 69.51; H, 4.32; N, 20.31%.

*N-(5-Cyano-1-methyl-1H-benzo[d]imidazol-2-yl)-2-methoxybenzamide* ***33***. From 0.10 g (0.6 mmol) of 2-methoxybenzoylchloride **20**, 0.10 g (0.6 mmol) of 2-amino-5-cyano-1-methylbenzimidazole **16** and 0.08 g (0.8 mmol) of Et_3_N in dry toluene (15 ml), 0.12 g (70%) of white powder was obtained. m.p. 283–288 °C; ^1^H NMR (400 MHz, DMSO-d_6_): δ/ppm = 8.11 (s, 1H, H_arom_), 7.89 (d, 1H, *J* = 8.4 Hz, H_arom_), 7.80 (td, 2H, *J*_1_ = 8.5 Hz, *J*_2_ = 1.6 Hz, H_arom_), 7.61 (td, 1H, *J*_1_ = 8.7 Hz, *J*_2_ = 1.7 Hz, H_arom_), 7.26 (d, 1H, *J* = 8.4 Hz, H_arom_), 7.14 (t, 1H, *J* = 7.5 Hz, H_arom_), 3.94 (s, 3H, CH_3_), 3.82 (s, 3H, CH_3_); ^13^C NMR (101 MHz, DMSO-d_6_): δ/ppm = 157.8 (2 C), 135.8 (2 C), 134.1, 130.9, 127.5, 121.1, 119.8 (2 C), 112.9 (2 C), 112.6, 105.7 (2 C), 56.6, 30.9; Anal. Calcd. for C_18_H_15_N_3_O: C, 66.66; H, 4.61; N, 18.29. Found: C, 66.70; H, 4.64; N, 18.31%.

*N-(6-Cyano-1-methyl-1H-benzo[d]imidazol-2-yl)-2,4-dimethoxybenzamide* ***34***. From 0.12 g (0.6 mmol) of 2,4-dimethoxybenzoylchloride **21**, 0.10 g (0.6 mmol) of 2-amino-5-cyano-1-methylbenzimidazole **16** and 0.07 g (0.7 mmol) of Et_3_N in dry toluene (15 ml), 0.13 g (65%) of light grey powder was obtained. m.p. 235–240 °C; ^1^H NMR (400 MHz, DMSO-d_6_): δ/ppm = 8.10 (s, 1H, H_arom_), 7.89 (d, 1H, *J* = 8.6 Hz, H_arom_), 7.85 (d, 1H, *J* = 8.4 Hz, H_arom_), 7.76 (dd, 1H, *J*_1_ = 8.4 Hz, *J*_2_ = 1.4 Hz, H_arom_), 6.76 (d, 1H, *J* = 2.1 Hz, H_arom_), 6.73 (dd, 1H, *J*_1_ = 8.7 Hz, *J*_2_ = 2.2 Hz, H_arom_), 3.98 (s, 3H, CH_3_), 3.88 (s, 3H, CH_3_), 3.77 (s, 3H, CH_3_); ^13^C NMR (101 MHz, DMSO-d_6_): δ/ppm = 166.2, 133.3 (2 C), 132.0, 126.9, 126.0, 120.1 (2 C), 112.3, 106.7, 105.2 (2 C), 99.2 (2 C), 83.0, 81.6, 56.8, 56.2, 30.8; Anal. Calcd. for C_18_H_15_N_3_O: C, 64.28; H, 4.79; N, 16.66. Found: C, 64.31; H, 4.74; N, 16.67%.

*N-(6-Cyano-1-methyl-1H-benzo[d]imidazol-2-yl)-3,4,5-trimethoxybenzamide* ***35***. From 0.13 g (0.6 mmol) of 3,4,5-trimethoxybenzoylchloride **22**, 0.10 g (0.6 mmol) of 2-amino-5-cyano-1-methylbenzimidazole **16** and 0.07 g (0.7 mmol) of Et_3_N in dry toluene (15 ml), 0.14 g (68%) of white powder was obtained. m.p. 290–295 °C; ^1^H NMR (400 MHz, DMSO-d_6_): δ/ppm = 12.88 (s, 1H, NH_amide_), 7.82 (s, 1H, H_arom_), 7.71 (d, 1H, *J* = 8.1 Hz, H_arom_), 7.65 (d, 1H, *J* = 8.3 Hz, H_arom_), 7.59 (s, 2H, H_arom_), 3.87 (s, 9H, CH_3_), 3.33 (s, 3H, CH_3_); ^13^C NMR (101 MHz, DMSO-d_6_): δ/ppm = 173.9, 153.8, 152.8 (2 C), 141.0, 134.1, 133.6, 129.6, 127.5, 119.9, 115.5, 111.1, 106.7, 106.3 (2 C), 104.6, 60.6, 56.3 (2 C), 29.1; Anal. Calcd. for C_18_H_15_N_3_O: C, 62.29; H, 4.95; N, 15.29. Found: C, 62.31; H, 5.04; N, 15.26%.

N-(1-Isobutyl-1H-benzo[d]imidazol-2-yl)benzamide ***36***. From 0.07 g (0.5 mmol) of benzoylchloride **23**, 0.10 g (0.5 mmol) of 2-amino-1-isobutylbenzimidazole **14** and 0.06 g (0.6 mmol) of Et_3_N in dry toluene (15 ml), 0.09 g (55%) of light orange powder was obtained. m.p. 145–150 °C; ^1^H NMR (400 MHz, DMSO-d_6_): δ/ppm = 8.18 (dd, 2H, *J*_1_ = 8.4 Hz, *J*_2_ = 1.5 Hz, H_arom_), 7.68 (d, 1H, *J* = 7.3 Hz, H_arom_), 7.64 (d, 1H, *J* = 8.5 Hz, H_arom_), 7.59 (d, 1H, *J* = 7.2 Hz, H_arom_), 7.56–7.52 (m, 2H, H_arom_), 7.38–7.31 (m, 2H, H_arom_), 4.24 (d, 2H, *J* = 7.3 Hz, CH_2_), 2.35–2.25 (m, 1H, CH), 0.96 (d, 6H, *J* = 6.7 Hz, CH_3_); ^13^C NMR (101 MHz, DMSO-d_6_): δ/ppm = 132.3, 130.2 (5 C), 129.3 (2 C), 128.7 (2 C), 124.0 (2 C), 113.2, 111.2, 49.7, 28.2, 20.3 (2 C); Anal. Calcd. for C_18_H_15_N_3_O: C, 73.69; H, 6.53; N, 14.32. Found: C, 73.61; H, 6.54; N, 14.26%.

*N-(1-Isobutyl-1H-benzo[d]imidazol-2-yl)-2-methoxybenzamide* ***37***. From 0.09 g (0.5 mmol) of 2-methoxybenzoylchloride **20**, 0.10 g (0,5 mmol) of 2-amino-1-isobutylbenzimidazole **14** and 0.10 g (0.8 mmol) of Et_3_N in dry toluene (15 ml), 0.05 g (27%) of orange oil was obtained. ^1^H NMR (300 MHz, DMSO-d_6_): δ/ppm = 7.62 (dd, 1H, *J*_1_ = 7.6 Hz, *J*_2_ = 1.7 Hz, H_arom_), 7.52–7.45 (m, 1H, H_arom_), 7.28–7.23 (m, 1H, H_arom_), 7.22–7.18 (m, 1H, H_arom_), 7.11 (d, 1H, *J* = 8.3 Hz, H_arom_), 7.04–6.94 (m, 1H, H_arom_), 3.84 (d, 2H, *J* = 7.6 Hz, CH_2_), 3.81 (s, 3H, CH_3_), 2.20–2.05 (m, 1H, CH), 0.88 (d, 6H, *J* = 6.6 Hz, CH3); ^13^C NMR (151 MHz, DMSO-d_6_): δ/ppm = 167.8, 157.9, 153.6, 138.4, 133.4, 132.7, 130.4, 121.9, 121.0, 119.9, 119.3, 113.6, 112.3, 108.6, 55.6 (2 C), 48.4, 27.6, 19.5 (2 C); Anal. Calcd. for C_18_H_15_N_3_O: C, 70.57; H, 6.55; N, 12.99. Found: C, 70.53; H, 6.54; N, 11.86%.

*N-(1-Isobutyl-1H-benzo[d]imidazol-2-yl)-2,4-dimethoxybenzamide* ***38***. From 0.11 g (0.5 mmol) of 2,4-dimethoxybenzoylchloride **21**, 0.10 g (0,5 mmol) of 2-amino-1-isobutylbenzimidazole **14** and 0.06 g (0.6 mmol) of Et_3_N in dry toluene (15 ml), 0.06 g (34%) of light brown powder was obtained. m.p. 128–133 °C; ^1^H NMR (300 MHz, DMSO-d_6_): δ/ppm = 7.70 (d, 1H, *J* = 8.6 Hz, H_arom_), 7.36 (dd, 1H, *J*_1_ = 6.0 Hz, *J*_2_ = 2.7 Hz, H_arom_), 7.27 (dd, 1H, *J*_1_ = 6.1 Hz, *J*_2_ = 2.6 Hz, H_arom_), 7.14–7.06 (m, 2H, H_arom_), 6.65–6.55 (m, 2H, H_arom_), 3.89 (d, 2H, *J* = 7.6 Hz, CH_2_), 3.82 (d, 6H, *J* = 3.3 Hz, CH_2_), 2.18–2.07 (m, 1H, CH), 0.89 (d, 6H, *J* = 6.6 Hz, CH_3_); ^13^C NMR (101 MHz, DMSO-d_6_): δ/ppm = 173.9, 153.8, 152.8 (2 C), 141.0, 134.1, 133.6, 129.6, 127.5, 119.9, 115.5, 111.1, 106.7, 106.3 (2 C), 104.6, 60.6, 56.3 (2 C), 29.1; Anal. Calcd. for C_18_H_15_N_3_O: C, 67.97; H, 6.56; N, 11.89. Found: C, 67.91; H, 6.54; N, 11.86%.

*N-(1-Isobutyl-1H-benzo[d]imidazol-2-yl)-3,4,5-trimethoxybenzamide* ***39***. From 0.12 g (0.5 mmol) of 3,4,5-trimethoxybenzoylchloride **22**, 0.10 g (0.5 mmol) of 2-amino-1-isobutylbenzimidazole **14** and 0.06 g (0.6 mmol) of Et_3_N in dry toluene (15 ml), 0.11 g (55%) of light yellow powder was obtained. m.p. 154–158 °C; ^1^H NMR (600 MHz, DMSO-d_6_): δ/ppm = 12.65 (s, 1H, NH_amide_), 7.58 (s, 1H, H_arom_), 7.52 (t, 2H, *J* = 7.5 Hz, H_arom_), 7.24 (td, 1H, *J*_1_ = 7.7 Hz, *J*_2_ = 3.8 Hz, H_arom_), 7.21 (td, 1H, *J*_1_ = 7.5 Hz, *J*_2_ = 1.0 Hz, H_arom_), 4.07 (d, 2H, *J* = 7.2 Hz, CH_2_), 3.86 (s, 6H, CH_3_), 3.73 (s, 3H, CH_3_), 2.38–2.32 (m, 1H, CH), 0.97 (d, 6H, *J* = 6.7 Hz, CH_3_); ^13^C NMR (151 MHz, DMSO-d_6_): δ/ppm = 172.6, 152.3, 152.2 (2 C), 140.0, 133.7, 129.8, 128.8, 122.6, 122.4, 111.9, 109.7, 106.0 (2 C), 60.0, 55.6 (2 C), 48.9, 27.8, 20.0 (2 C); Anal. Calcd. for C_18_H_15_N_3_O: C, 65.78; H, 6.57; N, 10.96. Found: C, 65.69; H, 6.54; N, 10.86%.

*N-(6-Cyano-1-isobutyl-1H-benzo[d]imidazol-2-yl)benzamide* ***40***. From 0.06 g (0.5 mmol) of benzoylchloride **23**, 0.10 g (0.5 mmol) of 2-amino-6-cyano-1-isobutylbenzimidazole **17** and 0.06 g (0.6 mmol) of Et_3_N in dry toluene (15 ml), 0.03 g (17%) of white powder was obtained. m.p. 213–217 °C; ^1^H NMR (600 MHz, DMSO-d_6_): δ/ppm = 8.23 (d, 2H, *J* = 7.3 Hz, H_arom_), 7.86 (s, 1H, H_arom_), 7.74 (d, 1H, *J* = 8.3 Hz, H_arom_), 7.70 (dd, 1H, *J*_1_ = 8.3 Hz, *J*_2_ = 1.5 Hz, H_arom_), 7.56–7.53 (m, 1H, H_arom_), 7.51–7.48 (m, 2H, H_arom_), 4.11 (d, 2H, *J* = 7.4 Hz, CH_2_), 2.32–2.25 (m, 1H, CH), 0.95 (d, 6H, *J* = 6.7 Hz, CH_3_); ^13^C NMR (151 MHz, DMSO-d_6_): δ/ppm = 172.6, 152.3, 152.2 (2 C), 140.0, 133.7, 129.8, 128.8, 122.6, 122.4, 111.9, 109.7, 106.0 (2 C), 60.0, 55.6 (2 C), 47.9, 27.8, 19.0 (2 C); Anal. Calcd. for C_18_H_15_N_3_O: C, 71.68; H, 5.70; N, 17.60. Found: C, 71.62; H, 5.74; N, 17.56%.

*N-(6-Cyano-1-isobutyl-1H-benzo[d]imidazol-2-yl)-2-methoxybenzamide* ***41***. From 0.08 g (0.5 mmol) of 2-methoxybenzoylchloride **20**, 0.10 g (0.5 mmol) of 2-amino-6-cyano-1-isobutylbenzimidazole **17** and 0.09 g (0.9 mmol) of Et_3_N in dry toluene (15 ml), 0.03 g (16%) of white powder was obtained. m.p. 196‒199 °C; ^1^H NMR (600 MHz, DMSO-d_6_): δ/ppm = 8.10 (s, 1H, H_arom_), 7.95 (d, 1H, *J* = 8.4 Hz, H_arom_), 7.79 (d, 1H, *J* = 8.5 Hz, H_arom_), 7.72 (dd, 1H, *J*_1_ = 7.6 Hz, *J*_2_ = 1.6 Hz, H_arom_), 7.59 (t, 1H, *J* = 7.5 Hz, H_arom_), 7.24 (d, 1H, *J* = 8.4 Hz, H_arom_), 7.12 (t, 1H, *J* = 7.4 Hz, H_arom_), 4.19 (d, 2H, *J* = 7.5 Hz, CH_2_), 3.91 (s, 3H, CH_3_), 2.25–2.18 (m, 1H, CH), 0.90 (d, 6H, *J* = 6.6 Hz, CH_3_); ^13^C NMR (151 MHz, DMSO-d_6_): δ/ppm = 157.2 (2 C), 133.3, 130.2 (2 C), 127.0, 120.6 (2 C), 119.2 (2 C), 112.5, 112.4, 105.2 (2 C), 56.0, 50.0, 27.8 (2 C), 19.5 (2 C); Anal. Calcd. for C_18_H_15_N_3_O: C, 68.95; H, 5.79; N, 16.08. Found: C, 68.89; H, 5.74; N, 16.09%.

*N-(6-Cyano-1-isobutyl-1H-benzo[d]imidazol-2-yl)-2,4-dimethoxybenzamide* ***42***. From 0.09 g (0,5 mmol) of 2,4-dimethoxybenzoylchloride **21**, 0,10 g (0,5 mmol) of 2-amino-6-cyano-1-isobutylbenzimidazole **17** and 0.06 g (0.6 mmol) of Et_3_N in dry toluene (15 ml), 0.02 g (8%) of yellow powder was obtained. m.p. 138–142 °C; ^1^H NMR (600 MHz, DMSO-d_6_): δ/ppm = 10.50 (s, 1H, H_arom_) 8.07 (bs, 1H, H_arom_), 7.82 (d, 2H, *J* = 8.0 Hz, H_arom_), 7.68 (d, 1H, *J* = 8.2 Hz, H_arom_), 6.72 (s, 1H, H_arom_), 6.68 (d, 1H, *J* = 8.0 Hz, H_arom_), 4.05 (d, 2H, *J* = 7.3 Hz, CH_2_), 3.94 (s, 3H, CH_3_), 3.86 (s, 3H, CH_3_), 2.20–2.13 (m, 1H, CH), 0.86 (d, 6H, *J* = 6.5 Hz, CH_3_); ^13^C NMR (151 MHz, DMSO-d_6_): δ/ppm = 159.2, 134.4, 132.5 (2 C), 125.9, 119.7 (2 C), 118.8, 115.3, 105.4, 104.0 (2 C), 98.7 (2 C), 56.1, 55.6 (2 C), 28.0, 19.7 (2 C); Anal. Calcd. for C_18_H_15_N_3_O: C, 66.65; H, 5.86; N, 14.81. Found: C, 66.70; H, 5.94; N, 14.79%.

*N-(6-Cyano-1-isobutyl-1H-benzo[d]imidazol-2-yl)-3,4,5-trimethoxybenzamide* ***43***. From 0.11 g (0.5 mmol) of 3,4,5-trimethoxybenzoylchloride **22**, 0.10 g (0.5 mmol) of 2-amino-6-cyano-1-isobutylbenzimidazole **17** and 0.06 g (0.6 mmol) of Et_3_N in dry toluene (15 ml), 0.12 g (62%) of white powder was obtained. m.p. 179–184 °C; ^1^H NMR (600 MHz, DMSO-d_6_): δ/ppm = 12.88 (s, 1H, NH_amide_), 7.83 (s, 1H, H_arom_), 7.73 (d, 1H, *J* = 8.3 Hz, H_arom_), 7.70 (dd, 1H, *J*_1_ = 8.3 Hz, *J*_2_ = 1.1 Hz, H_arom_), 7.57 (s, 2H, H_arom_), 4.09 (d, 2H, *J* = 7.1 Hz, CH_2_), 3.86 (s, 9H, CH_3_), 2.36–2.32 (m, 1H, CH), 0.97 (d, 6H, *J* = 6.4 Hz, CH_3_); ^13^C NMR (151 MHz, DMSO-d_6_): δ/ppm = 173.2, 153.2, 152.3, 133.4, 133.1, 129.1, 127.0, 119.4, 115.1, 110.8, 106.1 (2 C), 104.0 (2 C), 60.1, 55.6 (2 C), 49.2, 27.7, 19.9 (2 C); Anal. Calcd. for C_18_H_15_N_3_O: C, 64.69; H, 5.92; N, 13.72. Found: C, 64.95; H, 5.94; N, 13.69%.

*N-(1-Phenyl-1H-benzo[d]imidazol-2-yl)benzamide* ***44***. From 0.07 g (0.5 mmol) of benzoylchloride **23**, 0.10 g (0.5 mmol) of 2-amino-1-phenylbenzimidazole **15** and 0.06 g (0.6 mmol) of Et_3_N in dry toluene (15 ml), 0.06 g (38%) of yellow powder was obtained. m.p. 220–223 °C; ^1^H NMR (600 MHz, DMSO-d_6_): δ/ppm = 13.01 (s, 1H, NH_amide_), 8.04 (d, 2H, *J* = 5.5 Hz, H_arom_), 7.72–7.66 (m, 4H, H_arom_), 7.63 (d, 1H, *J* = 7.4 Hz, H_arom_), 7.57 (s, 1H, H_arom_), 7.47 (s, 1H, H_arom_), 7.42–7.38 (m, 2H, H_arom_), 7.30–7.28 (m, 1H, H_arom_), 7.25–7.22 (m, 1H, H_arom_), 7.17 (d, 1H, *J* = 7.1 Hz, H_arom_); ^13^C NMR (151 MHz, DMSO-d_6_): δ/ppm = 173.9, 152.5, 137.9, 134.2, 131.0, 129.6, 129.3 (2 C), 129.2, 128.7, 128.4 (2 C), 127.9 (2 C), 127.3, 123.2, 123.0 (2 C), 112.3, 109.6; Anal. Calcd. for C_18_H_15_N_3_O: C, 76.66; H, 4.83; N, 13.41. Found: C, 76.99; H, 4.94; N, 13.39%.

*2-Methoxy-N-(1-phenyl-1H-benzo[d]imidazol-2-yl)benzamide* ***45***. From 0.08 g (0.5 mmol) of 2-methoxybenzoylchloride **20**, 0.10 g (0.5 mmol) of 2-amino-1-phenylbenzimidazole **15** and 0.10 g (1.0 mmol) of Et_3_N in dry toluene (15 ml), 0.09 g (52%) of orange powder was obtained. m.p. 200–203 °C; ^1^H NMR (400 MHz, DMSO-d_6_): δ/ppm = 7.99 (dd, 1H, *J*_1_ = 7.8 Hz, *J*_2_ = 1.5 Hz, H_arom_), 7.87 (d, 1H, *J* = 8.0 Hz, H_arom_), 7.85–7.77 (m, 5H, H_arom_), 7.68 (td, 1H, *J*_1_ = 8.7 Hz, *J*_2_ = 1.8 Hz, H_arom_), 7.54 (td, 1H, *J*_1_ = 8.1 Hz, *J*_2_ = 0.9 Hz, H_arom_), 7.47 (td, 1H, *J*_1_ = 8.2 Hz, *J*_2_ = 1.0 Hz, H_arom_), 7.30 (d, 1H, *J* = 8.0 Hz, H_arom_), 7.24 (d, 1H, *J* = 8.3 Hz, H_arom_), 7.20 (t, 1H, *J* = 7.5 Hz, H_arom_), 3.60 (s, 3H, CH_3_); ^13^C NMR (101 MHz, DMSO-d_6_): δ/ppm = 158.0 (5 C), 136.1, 131.7, 131.5, 131.3 (2 C), 128.1 (2 C), 125.8, 122.1, 115.4, 113.4, 111.5, 56.8; Anal. Calcd. for C_18_H_15_N_3_O: C, 73.45; H, 4.99; N, 12.24. Found: C, 73.49; H, 5.04; N, 12.19%.

*2,4-Dimethoxy-N-(1-phenyl-1H-benzo[d]imidazol-2-yl)benzamide* ***46***. From 0.10 g (0.5 mmol) of 2,4-dimethoxybenzoylchloride **21**, 0.10 g (0.5 mmol) of 2-amino-1-phenylbenzimidazole **15** and 0.06 g (0.6 mmol) of Et_3_N in dry toluene (15 ml), 0.10 g (57%) of light yellow powder was obtained. m.p. 218–222 °C; ^1^H NMR (400 MHz, DMSO-d_6_): δ/ppm = 8.03 (d, 1H, *J* = 8.8 Hz, H_arom_), 7.86–7.81 (m, 6H, H_arom_), 7.53 (t, 1H, *J* = 7.4 Hz, H_arom_), 7.46 (t, 1H, *J* = 7.3 Hz, H_arom_), 7.29 (d, 1H, *J* = 8.0 Hz, H_arom_), 6.81 (dd, 1H, *J*_1_ = 8.9 Hz, *J*_2_ = 2.2 Hz, H_arom_), 6.73 (d, 1H, *J* = 2.2 Hz, H_arom_), 3.88 (s, 3H, CH_3_), 3.55 (s, 3H, CH_3_); ^13^C NMR (101 MHz, DMSO-d_6_): δ/ppm = 159.9, 134.0, 131.4 (2 C), 128.2 (2 C), 125.7, 115.4, 111.5, 108.1, 99.5, 57.0, 56.5; Anal. Calcd. for C_18_H_15_N_3_O: C, 70.76; H, 5.13; N, 11.25. Found: C, 70.79; H, 5.14; N, 11.19%.

*3,4,5-Trimethoxy-N-(1-pheny-1H-benzo[d]imidazol-2-yl)benzamide* ***47***. From 0.11 g (0.5 mmol) of 3,4,5-trimethoxybenzoylchloride **22**, 0.10 g (0.5 mmol) of 2-amino-1-phenylbenzimidazole **15** and 0.06 g (0.6 mmol) of Et_3_N in dry toluene (15 ml), 0.13 g (69%) of light brown powder was obtained. m.p. 139–143 °C; ^1^H NMR (600 MHz, DMSO-d_6_): δ/ppm = 12.86 (s, 1H, H_arom_), 7.73 (bs, 2H, H_arom_), 7.66–7.63 (m, 3H, H_arom_), 7.54 (t, 1H, *J* = 7.3 Hz, H_arom_), 7.39 (bs, 2H, H_arom_), 7.31–7.28 (m, 1H, H_arom_), 7.27–7.23 (m, 2H, H_arom_), 3.76 (s, 6H, CH_3_), 3.70 (s, 3H, CH_3_); ^13^C NMR (151 MHz, DMSO-d6): δ/ppm = 173.9, 152.5, 137.9, 134.2, 131.0, 129.6, 129.3 (2 C), 129.2, 128.7, 128.4 (2 C), 127.9 (2 C), 127.3, 123.2, 123.0 (2 C), 112.3, 109.6; Anal. Calcd. for C_18_H_15_N_3_O: C, 68.47; H, 5.25; N, 10.42. Found: C, 68.49; H, 5.34; N, 10.49%.

*N-(6-Cyano-1-phenyl-1H-benzo[d]imidazol-2-yl)benzamide* ***48***. From 0.06 g (0.4 mmol) of benzoylchloride **23**, 0.10 g (0.4 mmol) of 2-amino-6-cyano-1-phenylbenzimidazole **18** and 0.05 g (0.5 mmol) of Et_3_N in dry toluene (15 ml), 0.09 g (61%) of light yellow powder was obtained. m.p. 242–245 °C; ^1^H NMR (600 MHz, DMSO-d_6_): δ/ppm = 13.15 (s, 1H, NH_amide_) 7.99 (s, 2H, H_arom_), 7.67–7.65 (m, 6H, H_arom_), 7.58 (s, 1H, H_arom_), 7.50 (d, 1H, *J* = 6.5 Hz, H_arom_), 7.42 (t, 2H, *J* = 7.3 Hz, H_arom_), 7.31 (s, 1H, H_arom_); ^13^C NMR (151 MHz, DMSO-d_6_): δ/ppm = 129.5, 128.9, 128.1, 119.4, 104.8; Anal. Calcd. for C_18_H_15_N_3_O: C, 74.54; H, 4.17; N, 16.56. Found: C, 74.49; H, 4.14; N, 16.49%.

*N-(6-Cyano-1-phenyl-1H-benzo[d]imidazol-2-yl)-2-methoxybenzamide* ***49***. From 0.07 g (0.4 mmol) of 2-methoxybenzoylchloride **20**, 0.10 g (0.4 mmol) of 2-amino-6-cyano-1-phenylbenzimidazole **18** and 0.08 g (0.8 mmol) of Et_3_N in dry toluene (15 ml), 0.07 g (46%) of white powder was obtained. m.p. 226–230 °C; ^1^H NMR (400 MHz, DMSO-d_6_): δ/ppm = 8.24 (s, 1H, H_arom_), 7.74–7.63 (m, 7H, H_arom_), 7.56 (dd, 1H, *J*_1_ = 11.4 Hz, *J*_2_ = 4.2 Hz, H_arom_), 7.38 (d, 1H, *J* = 8.4 Hz, H_arom_), 7.16 (d, 1H, *J* = 8.4 Hz, H_arom_), 7.07 (t, 1H, *J* = 7.5 Hz, H_arom_), 3.70 (s, 3H, CH_3_); ^13^C NMR (101 MHz, DMSO-d_6_): δ/ppm = 157.6, 137.0, 134.4, 133.6 (2 C), 131.2 (2 C), 130.6 (2 C), 130.2, 127.5, 127.4 (2 C), 121.4, 119.9 (2 C), 112.9 (2 C), 112.0, 105.6 (2 C), 56.5; Anal. Calcd. for C_18_H_15_N_3_O: C, 71.73; H, 4.38; N, 15.21. Found: C, 71.70; H, 4.34; N, 15.18%.

*N-(6-Cyano-1-phenyl-1H-benzo[d]imidazol-2-yl)-2,4-dimethoxybenzamide* ***50***. From 0.09 g (0.4 mmol) of 2,4-dimethoxybenzoylchloride **21**, 0.10 g (0.4 mmol) of 2-amino-6-cyano-1-phenylbenzimidazole **18** and 0.05 g (0.5 mmol) of Et_3_N in dry toluene (15 ml), 0.01 g (8%) of white powder was obtained. m.p. 182‒185 °C; ^1^H NMR (400 MHz, DMSO-d_6_): δ/ppm = 10.36 (s, 1H, NH_amide_), 8.24 (s, 1H, H_arom_), 7.76 (d, 1H, *J* = 9.3 Hz, H_arom_), 7.68–7.61 (m, 6H, H_arom_), 7.33 (d, 1H, *J* = 8.4 Hz, H_arom_), 6.68–6.63 (m, 2H, H_arom_), 3.83 (s, 3H, CH_3_), 3.70 (s, 3H, CH_3_); ^13^C NMR (101 MHz, DMSO-d_6_): δ/ppm = 164.5, 163.1, 159.3, 147.7, 141.3, 137.8, 134.1, 133.4, 130.6 (2 C), 129.9, 127.4 (2 C), 126.9, 123.8, 120.2, 113.4, 111.6, 106.9, 105.0, 99.1, 56.6, 56.2; Anal. Calcd. for C_18_H_15_N_3_O: C, 69.34; H, 4.55; N, 14.06. Found: C, 69.36; H, 4.34; N, 14.18%.

*N-(6-Cyano-1-phenyl-1H-benzo[d]imidazol-2-yl)-3,4,5-trimethoxybenzamide* ***51***. From 0.10 g (0.4 mmol) of 3,4,5-trimethoxybenzoylchloride **22**, 0.10 g (0.4 mmol) of 2-amino-6-cyano-1-phenylbenzimidazole **18** and 0.05 g (0.5 mmol) of Et_3_N in dry toluene (15 ml), 0.07 g (39%) of white powder was obtained. m.p. 169–173 °C; ^1^H NMR (400 MHz, DMSO-d_6_): δ/ppm = 13.07 (s, 1H, NH_amide_), 7.91 (s, 1H, H_arom_), 7.76 (d, 2H, *J* = 7.7 Hz, H_arom_), 7.69–7.66 (m, 3H, H_arom_), 7.59 (d, 1H, *J* = 7.3 Hz, H_arom_), 7.42 (s, 1H, H_arom_), 7.39 (s, 1H, H_arom_), 7.37 (d, 1H, *J* = 8.4 Hz, H_arom_), 3.88 (s, 3H, CH_3_), 3.75 (s, 6H, CH_3_); ^13^C NMR (101 MHz, DMSO-d_6_): δ/ppm = 173.8, 156.4, 153.1, 152.7 (2 C), 140.9, 133.5, 133.4, 129.7, 129.2, 128.0, 127.8, 115.8, 111.1, 107.0 (2 C), 106.6 (2 C), 105.3 (2 C), 100.5, 60.6, 56.4, 56.0; Anal. Calcd. for C_18_H_15_N_3_O: C, 67.28; H, 4.71; N, 13.08. Found: C, 67.36; H, 4.64; N, 12.98%.

### 2.2. Biology

#### 2.2.1. Antioxidant activity

##### 2.2.1.1. Determination of the reducing activity of the stable radical 1,1-diphenyl-picrylhydrazyl (DPPH)

The reducing activity of investigated systems was achieved by the DPPH method according to previously described procedures with modifications to assure the use in a 96-well microplate. Briefly, equal volumes of various concentrations of tested molecules (dissolved in DMSO) were added to the solution of DPPH (final concentration 50 µM in absolute ethanol). Ethanol and DMSO were used as control solutions in line with earlier reports.^27^

##### 2.2.1.2. Determination of Ferric Reducing/Antioxidant Power (FRAP assay)

The FRAP method was carried out according to previously described procedures with some modifications to be compatible with an assay on a 96-well microplate. All results were expressed as Fe^2+^ equivalents (Fe^2+^ µmol). All tests were done in triplicate and the results were averaged and presented in Table 1^27^.

**Table 1.** IC_50_ values for 1,1-diphenyl-picrylhydrazyl (DPPH) free radical scavenging and ferricreducing/antioxidant power (FRAP) activities.

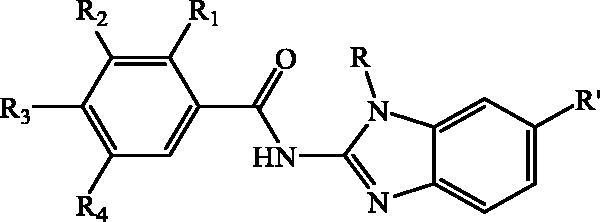								
Cpd	R_1_	R_2_	R_3_	R_4_	R'	R	DPPH mM	FRAP mmolFe^2+^/ mmolC
**24**	H	H	H	H	H	H	360.55 ± 6.36	–
**25**	OCH_3_	H	H	H	H	H	75.72 ± 6.08	–
**26**	OCH_3_	H	OCH_3_	H	H	H	41.69 ± 1.51	166.61 ± 9.51
**27**	H	OCH_3_	OCH_3_	OCH_3_	H	H	6.82 ± 0.41	5.59 ± 0.62
**28**	H	H	H	H	H	CH_3_	3.78 ± 0.03	358.81 ± 4.83
**29**	OCH_3_	H	H	H	H	CH_3_	6.05 ± 1.03	110.16 ± 1.44
**30**	OCH_3_	H	OCH_3_	H	H	CH_3_	49.62 ± 18.38	–
**31**	H	OCH_3_	OCH_3_	OCH_3_	H	CH_3_	6.036 ± 0.29	124.28 ± 1.23
**32**	H	H	H	H	CN	CH_3_	429.0 ± 16.33	160.27 ± 16.35
**33**	OCH_3_	H	H	H	CN	CH_3_	4.82 ± 0.08	429.60 ± 3.85
**34**	OCH_3_	H	OCH_3_	H	CN	CH_3_	5.68 ± 0.31	618.10 ± 66.10
**35**	H	OCH_3_	OCH_3_	OCH_3_	CN	CH_3_	–	338.50 ± 21.56
**36**	H	H	H	H	H	CH_2_CH(CH_3_)_2_	4.51 ± 0.02	179.70 ± 23.42
**37**	OCH_3_	H	H	H	H	CH_2_CH(CH_3_)_2_	10.55 ± 0.03	–
**38**	OCH_3_	H	OCH_3_	H	H	CH_2_CH(CH_3_)_2_	26.58 ± 0.02	5.80 ± 0.15
**39**	H	OCH_3_	OCH_3_	OCH_3_	H	CH_2_CH(CH_3_)_2_	4.92 ± 0.13	217.20 ± 3.69
**40**	H	H	H	H	CN	CH_2_CH(CH_3_)_2_	11.47 ± 1.5	203.50 ± 10.53
**41**	OCH_3_	H	H	H	CN	CH_2_CH(CH_3_)_2_	4.58 ± 0.15	154.30 ± 5.67
**42**	OCH_3_	H	OCH_3_	H	CN	CH_2_CH(CH_3_)_2_	5.35 ± 0.07	–
**43**	H	OCH_3_	OCH_3_	OCH_3_	CN	CH_2_CH(CH_3_)_2_	2054 ± 77.78	243.30 ± 3.39
**44**	H	H	H	H	H	Phenyl	1933.75 ± 34.29	184.70 ± 3.22
**45**	OCH_3_	H	H	H	H	Phenyl	8.71 ± 0.34	–
**46**	OCH_3_	H	OCH_3_	H	H	Phenyl	8.48 ± 0.71	–
**47**	H	OCH_3_	OCH_3_	OCH_3_	H	Phenyl	1211.70 ± 115.54	236.90 ± 1.50
**48**	H	H	H	H	CN	Phenyl	26090 ± 173.52	138.98 ± 32.68
**49**	OCH_3_	H	H	H	CN	Phenyl	29.885 ± 1.30	221.31 ± 27.39
**50**	OCH_3_	H	OCH_3_	H	CN	Phenyl	520.35 ± 30.17	506.70 ± 7.46
**51**	H	OCH_3_	OCH_3_	OCH_3_	CN	Phenyl	8745.50 ± 234.12	132.56 ± 18.06
BHT	–	–	–	–	–	–	0.025 ± 4.2	679.15

#### 2.2.2. Antiproliferative activity in vitro

The experiments were performed on four human cell lines, including HCT 116 (colon carcinoma), H 460 (lung carcinoma), MCF-7 (breast carcinoma) and HEK 293 (human embryonic kidney cells), in line with previously published experimental procedures^10^^,^^25^. Briefly, the cells were grown in DMEM medium with the addition of 10% foetal bovine serum (FBS), 2 mM l-glutamine, 100 U/mL penicillin and 100 µg/mL streptomycin, and cultured as monolayers at 37 °C in a humidified atmosphere with 5% CO_2_. Cells were seeded at 2 × 10^3^ cells/well in a standard 96-well microtiter plates and left to attach for 24 h. Next day, a test compound was added in five serial 10-fold dilutions. The rate of cell growth was evaluated after 72 h of incubation with MTT reagent. The obtained results are expressed as IC_50_ values, calculated from the concentration-response curves using linear regression analysis by fitting the test concentrations that give PG values above and below the reference value (i.e. 50%). Each test was performed in quadruplicate in at least two individual experiments.

#### 2.2.3. Antioxidative activity assay in cells

For the antioxidative activity assay, 2.5 × 10^4^ cells were seeded into 96-well microtiter plates and left to attach for 24 h. Next day, cells were washed with PBS and incubated in FBS-free DMEM medium with 25 µM DCFH-DA fluorescence dye^27^. After 45 min of incubation, medium was discarded and cells were washed with PBS. After the washing step, cells were incubated with 100 µM *tert*-Butyl hydroperoxide (TBHP) alone or in combination with antioxidative agents (50 mM N-Acetyl-l-cysteine – NAC or 10 µM tested compounds) in PBS, for 1 h at 37 °C. DCFH-DA fluorescence was recorded on a microplate fluorimeter reader (Tecan) with excitation beam of 485 nm, while the emitted fluorescence was collected at 535 nm. All tests were presented as means of two independent measurements, done in triplicates. One-way ANOVA with Tukey’s *post hoc* test was used for statistical analysis, *p < .05, **p < .01, ***p < .001.

#### 2.2.4. Western blot analysis

Cell line HCT116 was seeded in 60 mm cell culture dishes (TPP, Switzerland) in a concentration of 9 × 10^5^ cells/dish. Next day, the cells were treated with 4 mM hydrogen peroxide as a positive control for ROS induction and with compounds **34** and **40** (50 µM and 5 µM, respectively) for 1 h. Nuclear extracts from the cells were prepared using nuclear extraction buffer kit (Abcam, Cambridge, UK, ab113474) according to the manufacturer instructions. BCA assay (Sigma Aldrich, Taufkirchen, Germany) was performed for the determination of the protein concentration in nuclear and cytoplasm fractions. Denaturation of 1 μg of total proteins in Laemmli SDS loading buffer was performed at 95 °C for 5 min. After loading, 10% SDS poly-acrylamide gel electrophoresis was carried out and then proteins were transferred to nitrocellulose membranes. Afterwards, the membranes were blocked in blocking buffer (ImmobilonBlock – CH (Chemiluminescent Blocker), MerckKGaA, Darmstadt, Germany) and incubated 5 h at room temperature with primary antibody dissolved in ImmobilonBlock – CH (Chemiluminescent Blocker). After the membranes were washed three times for five minutes each with Tris-buffered saline with 0.5% Tween 20 (TBST), they were incubated in the corresponding secondary antibody for 1 h at room temperature or overnight at 4 °C. After washing with TBST, the membranes were processed for enhanced chemiluminescence (ECL) Clarity Max Western ECL Substrate (Bio-Rad Laboratories, Hercules, CA, USA) and captured using Odyssey Fc (LICOR, Bad Homburg, Germany). The primary antibody used was anti-Nrf2, (dilution 1:500, Anti-Nrf2 antibody: ab133347, Abcam), Histon H3 (dilution 1:2000, Anti-H3 antibody: Ab1791, Abcam), β-actin (dilution 1:500, sc-47778, Santa Cruz Biotechnology) and corresponding secondary HRP-conjugated IgG antibody (dilution 1:2000, mouse IgGκ, Santa Cruz Biotechnology).

### 2.3. QSAR modelling

3D-QSAR models were generated using the data on antioxidative activities of presented compounds expressed as pIC_50_ (negative logarithm of IC_50_ value in mol dm^−3^ i.e. concentration that causes 50% antioxidative activity). A model for each test used in the presented manuscript, was generated: model **1** (DPPH) and model **2** (FRAP). In both cases five outliers were excluded in order to obtain predictive models: **30**, **35**, **43**, **47** and **49** for model **1**, **26**, **35**, **42**, **44** and **47** for model **2**. Molecular descriptors for each compound were generated with program Volsurf+^32^^,^^33^. Starting from the SMILES codes, 3-D structures were generated using VolSurf+ 3-D structure generator.

Considering high rigidity of the dataset compounds, VolSurf+ 3-D structure generator was considered to be powerful enough to produce the most relevant conformers and no additional conformational search was applied. Using following probes: H_2_O (the water molecule), O (sp^2^ carbonyl oxygen atom), N1 (neutral NH group (*e.g.* amide)), and DRY (the hydrophobic probe), Molecular Interaction Fields (MIFs) were calculated. From the MIFs, 128 descriptors with clear chemical and physical meaning were calculated for each compound. The detailed description of all 128 VolSurf + descriptors is given in the VolSurf + manual^32^^,^^33^. Partial Least Square (PLS) analysis was applied with goal of finding the relationship between the 3-D structure-based molecular descriptors and antioxidative activities. Models were built using raw data (without autoscaling pre-treatment of descriptors) and the quantitative influence of each descriptor on the anti-proliferative activity is given as product of the descriptor’s value and it’s PLS coefficient of the 3D-QSAR model. External validation of both models was performed using randomly chosen compounds for the test set: **28**, **31**, **39** and **48**, while the training set consisted of the remaining compounds of the dataset used to generate the model. Principal Component Analysis (PCA) was performed on the molecular descriptors of all compounds with measured antioxidative activity. PCA loadings were used for identification of the descriptors with the highest contribution to the overall variance in the X-space.

### 2.4. Statistical analysis

The GraphPad Prism 8 program was used for statistical analysis of the obtained experimental results. Data are expressed as means ± SD. A one-way ANOVA, followed by *post hoc* Tukey tests, was used to compare the treated groups to the control. Significant differences set up were at *p* < .05 and *p* < .01.

## 3. Results and discussion

### 3.1. Chemistry

**Scheme 1.**
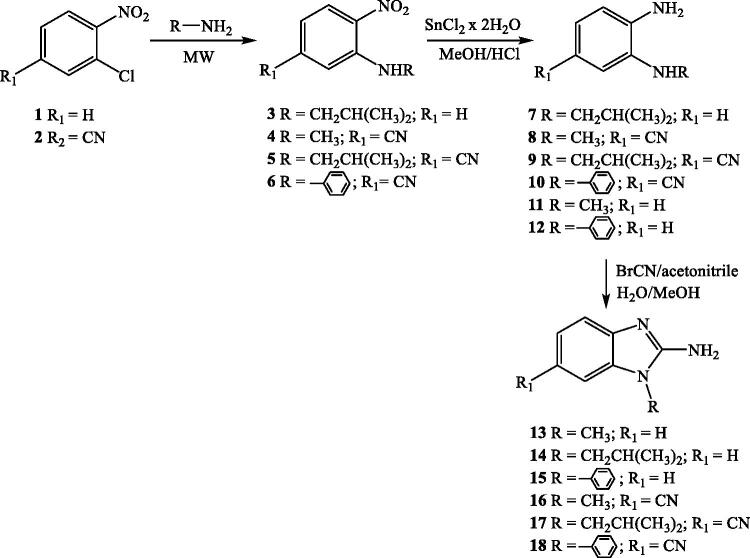
Synthesis of *N*-substituted 2-aminobenzimidazoles **13–18**.

**Scheme 2.**
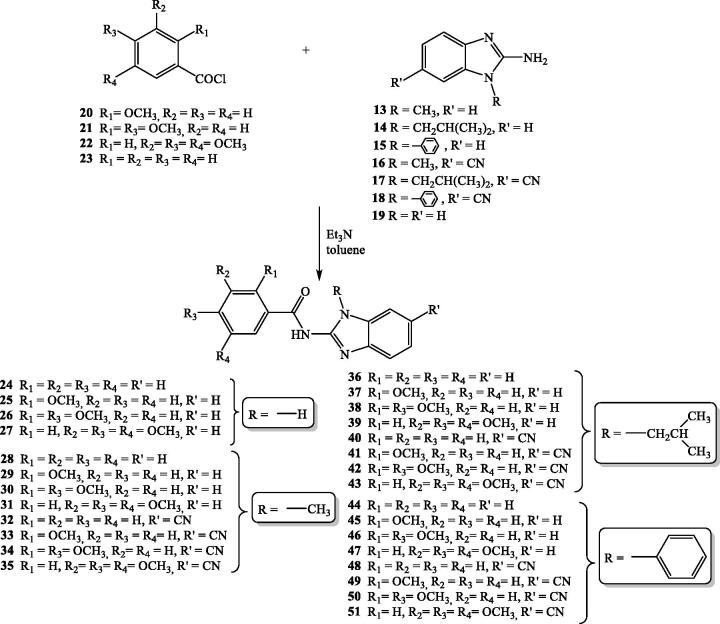
Synthesis of *N*-substituted benzimidazole-2-carboxamides **27–54**.

All designed *N*-substituted benzimidazole-2-amide derivatives **27–54** were synthesised following two synthetic procedures illustrated in reaction Schemes 1 and 2. The main precursors for the synthesis of targeted carboxamides, *N*-substituted 2-aminobenzimidazoles **13–18** were prepared according to the experimental procedure presented in Scheme 1 starting from the 2-chloronitrobenzene **1** or 2-chloro-4-cyanonitrobenzene **2**. Within the microwave-assisted uncatalyzed amination in acetonitrile with an excess of corresponding amine, 2-*N*-substituted nitrobenzenes **3**–**6** were prepared in moderate yields.

Reduction with SnCl_2 _×2H_2_O using MeOH and HCl_conc._ yielded 2-*N*-substituted 1,2-phenyalenediamines **7–10** while compounds **11–12** were commercially available. The key synthetic step was cyclocondensation of compounds **7–12** with cyanogenbromide in MeOH and water which gave corresponding *N*-substituted 2-aminobenzimidazoles **13–18** bearing either methyl, isobutyl or phenyl substituent at N atom of benzimidazole nuclei. 2-aminobenzimidazoles were prepared in moderate to high reaction yields.

Benzimidazole-2-carboxamides **24–51** were synthesised according to the reaction Scheme 2, following a classical method of organic synthesis for preparation of amides. Starting from the commercially available unsubstituted, mono-, di- and trimethoxy substituted benzoyl chlorides **20**–**23**, in the reaction with prepared *N*-substituted 2-aminobenzimidazoles **13–18** and commercially available 2-aminobenzimidazole **19**, in absolute toluene using trimethylamine as a weak base, targeted *N*-substituted benzimidazole-2-carboxamides **24–51** were prepared in moderate reaction yields.

The structures of all newly prepared *N*-substituted benzimidazole-2-carboxamides were characterised by means of ^1^H and ^13^C NMR spectroscopy and elemental analysis. The structure characterisation was performed based on the chemical shifts in both spectra and values of H-H coupling constants in the ^1^H spectra.

The amination reaction of reactants **1–2** resulted in the observation of signals related to amino substituents in the aliphatic and aromatic part of both ^1^H and ^13^C NMR spectra (signals in the range 8.19–9.90 ppm). Cylocondensation and formation of 2-aminobenzimidazoles **13–18** resulted in the downfield shift of the amino group.

Finally, the formation of the amide bond was confirmed within the presence of the signal related to amino group in the region 10.36–13.15 ppm. In addition, elemental analyses results also support that the compounds are in the proposed structures.

### 3.2. Biological activity

#### 3.2.1. Antioxidant activity

To determine the antioxidant activity of newly prepared N-substituted benzimidazole-2-carboxamides **24–51**, the reducing activity of the stable radical 1,1-diphenyl-picrylhydrazyl (DPPH) and ferric reducing/antioxidant power (FRAP) parameters were evaluated, respectively. Results were compared to a standard compound butylated hydroxytoluene (BHT) (Table 1).

The results for the DPPH and FRAP activities revealed that a number of tested compounds showed modest antioxidant activity. The exceptions are compound **35** which did not show any DPPH activity and compounds **24–25**, **30**, **37**, **42** and **45–46** which were not active in the FRAP assay. All tested compounds were in both assays significantly less active in comparison to standard BHT. Regarding the results obtained from the DPPH method, the most active compounds (with the IC_50_ ≤ 10 mM) were **27**–**29**, **31**, **33**–**34**, **36**–**37**, **39**–**42** and **45**–**46**. The most active one was unsubstituted derivative **28** bearing methyl group at the N atom of benzimidazole core (IC_50_ = 3.78 mM). Methoxy and trimethoxy substituted compounds **36, 41**, **33** and **39** showed also moderate activity in comparison to other tested derivatives with IC_50_ values 4.51 − 4.92 mM.

According to the results from the FRAP assay and the reducing power of the tested compounds, we can conclude that the most active derivatives were compounds **34** (dimethoxy substituted with methyl group at N atom and cyano group at benzimidazole nuclei) and **50** (dimethoxy substituted with phenyl ring at N atom and cyano group at benzimidazole nuclei). Somewhat lower activity was also obtained by methoxy substituted compound **33** with methyl group attached at N atom and cyano group at benzimidazole nuclei, unsubstituted compound **28** bearing methyl group at the N atom of benzimidazole core and trimethoxy substituted compound **35** bearing methyl group at N atom and cyano group at benzimidazole core showed moderate antioxidant activity. Finally, *N*-methyl substituted carboxamides bearing unsubstituted **28**, methoxy-**33** and dimethoxy substituted phenyl ring **34** as well as *N*-isobutyl substituted carboxamides bearing trimethoxy substituted **39** and unsubstituted phenyl ring **40** showed good antioxidant activity in both assays.

**Figure 2.**
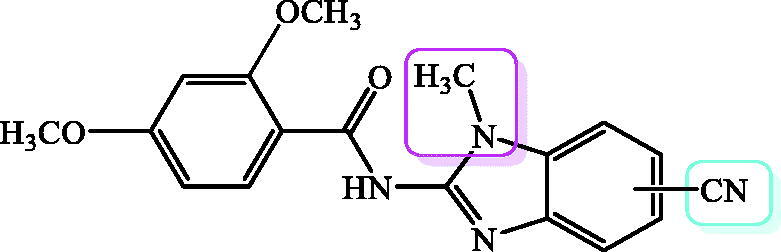
The most active potential antioxidant **34** chosen for further structure optimisation.

In conclusion, the results demonstrate that the number of methoxy groups as well as the type of the substituents placed either on the N atom or on the 5(6) position of benzimidazole core influenced the antioxidant activity. Isobutyl side chain placed at the N atom has the most significant influence on the antioxidant activity. The most promising antioxidant activity in both assays were displayed by dimethoxy substituted derivative **34** bearing methyl group at the N atom and CN group on the benzimidazole core (IC_50_ = 5.68 mM, 618.10 mmolFe^2+^/mmolC) which was chosen for further structure optimisation in order to improve antioxidant activity (Figure 2).

#### 3.2.2. Antiproliferative activity in vitro

**Figure 3.**
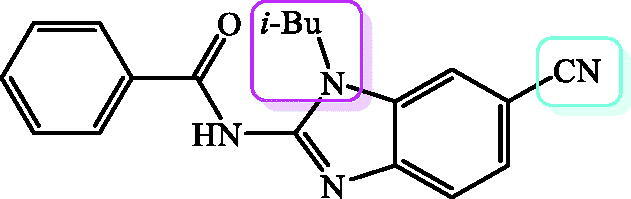
The most active derivative **40** chosen for further structure optimisation.

We further chose a number of *N*-substituted benzimidazole-2-carboxamides and tested their antiproliferative/cytotoxic activity *in vitro* on human cancer cells HCT116, MCF-7 and H 460, along with a non-tumour cell line – human embryonic kidney cells HEK 293. *Etoposide* was used as a standard antiproliferative agent. The obtained results are presented in Table 2. Obtained results pointed out that all tested compounds showed moderate to very strong antiproliferative activity. The most active ones were trimethoxy substituted derivative **43** (Figure 3) bearing isobutyl side chain at N atom and cyano group at the benzimidazole core with activity against HCT116 and H 460 cells in nanomolar range of inhibitory concentrations (IC_50_ 0.6 and 0.4 µM, respectively) and **40** showing similar activity.

**Table 2.** Antiproliferative activity of tested compounds

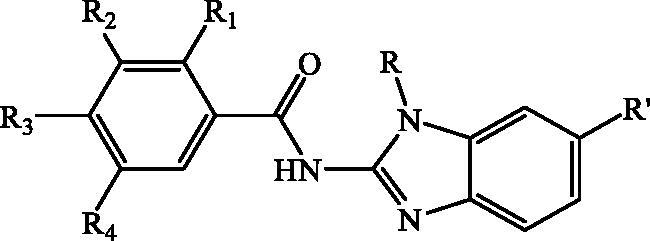										
							IC_50_^a^ (µM)			
Cpd	R_1_	R_2_	R_3_	R_4_	R'	R	HCT116	MCF-7	H 460	HEK 293
**27**	H	OCH_3_	OCH_3_	OCH_3_	H	H	23.1 ± 15.0	32.8 ± 15.6	17.6 ± 6.7	23.6 ± 4.4
**28**	H	H	H	H	H	CH_3_	18.7 ± 5.1	11.9 ± 7.5	10.7 ± 2.3	29.5 ± 12.5
**31**	H	OCH_3_	OCH_3_	OCH_3_	H	CH_3_	18.1 ± 1.4	23.8 ± 12.4	17.7 ± 0.8	21.7 ± 9.4
**33**	OCH_3_	H	H	H	CN	CH_3_	30.6 ± 2.0	12.5 ± 5.9	20.2 ± 3.3	22.7 ± 8.7
**34**	OCH_3_	H	OCH_3_	H	CN	CH_3_	47.6 ± 30.0	46.9 ± 35.4	21.9 ± 2.9	93.3 ± 2.3
**35**	H	OCH_3_	OCH_3_	OCH_3_	CN	CH_3_	5.2 ± 4.2	5.5 ± 3.2	3.9 ± 0.9	12.3 ± 1.5
**36**	H	H	H	H	H	CH_2_CH(CH_3_)_2_	3.8 ± 0.9	2.7 ± 1.9	5.1 ± 2.6	5.9 ± 2.4
**39**	H	OCH_3_	OCH_3_	OCH_3_	H	CH_2_CH(CH_3_)_2_	3.1 ± 0.7	4.6 ± 1.4	1.7 ± 0.1	2.0 ± 0.9
**40**	H	H	H	H	CN	CH_2_CH(CH_3_)_2_	0.6 ± 0.2	2.2 ± 1.9	2.5 ± 0.4	9.1 ± 1
**43**	H	OCH_3_	OCH_3_	OCH_3_	CN	CH_2_CH(CH_3_)_2_	0.6 ± 0.1	3.1 ± 1.7	0.4 ± 0.1	4.7 ± 1
**47**	H	OCH_3_	OCH_3_	OCH_3_	H	Phenyl	1.6 ± 0.9	2.9 ± 1.3	0.8 ± 1.5	5.3 ± 2.4
**51**	H	OCH_3_	OCH_3_	OCH_3_	CN	Phenyl	30.1 ± 13.2	29.4 ± 19.4	4.53 ± 7.3	61.1 ± 35.7
Etoposide	–	–	–	–	–	–	5 ± 2	1 ± 0.7	0.1 ± 0.04	–

^a^ IC_50_; the concentration that causes 50% growth inhibition.

Importantly, compound **40** possess more prominent effect towards tumour cancer cells, while being significantly less cytotoxic towards non-tumour cell line HEK 293. Trimethoxy substituted compound **47** bearing phenyl ring at the benzimidazole core showed selective activity against H 460 as well as trimethoxy substituted derivative **51** bearing phenyl ring at N atom and cyano group at the benzimidazole core.

The number of methoxy groups enhanced antiproliferative activity of compounds **33**–**35,** bearing methyl side chain at N atom and cyano group. In conclusion, the results showed that the cyano group placed at 5(6)-position of the benzimidazole core improved the antiproliferative activity when compared with compounds without substituent placed at 5(6)-position of benzimidazole core (**31** and **35**, **36** and **40**, **39** and **43**).

#### 3.2.3. Antioxidant ability in cells

We further selected two compounds **34** and **40**, showing quite pronounced antioxidant capacity in DPPH and FRAP assays, in order to test their antioxidant activity in tumour cells. We choose these compounds to test if their antiproliferative/cytotoxic activity can be related to their antioxidative ability, or some other mechanism(s). Specifically, **34** demonstrated very modest antiproliferative activities, while **40** was exceptionally cytotoxic towards HCT 116 cells. In addition, both compounds were significantly less toxic towards non-tumour cells.

For this reason, we treated HCT 116 cells with tert-Butyl hydroperoxide (TBHP), a substance commonly used for inducing oxidative stress in cells and tissues, alone or in a combination with a known antioxidative agent N-Acetyl-l-cysteine (NAC) or tested compounds^26^.

**Figure 4.**
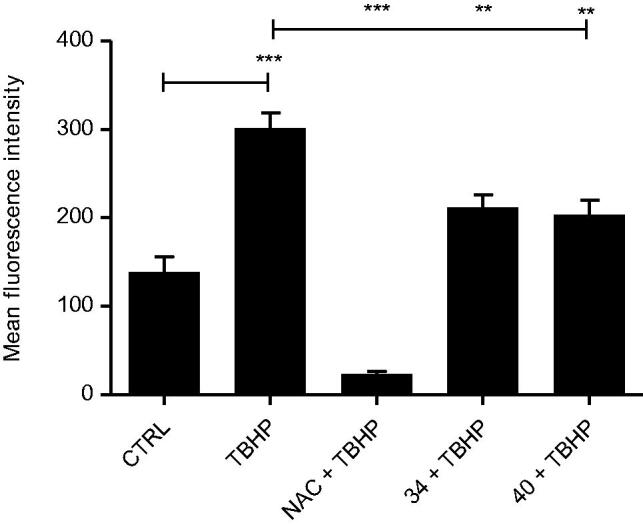
Antioxidative activity of selected compounds in cells. HCT 116 cells were treated with the combination of tert-Butyl hydroperoxide (TBHP, 200 µM) and N-Acetyl-l-cysteine (NAC, 10 mM) or tested compounds **34** and **40** (10 µM) for 1 h. The level of reactive oxygen species (ROS) was measured with fluorescent dye 2′,7′-dichlorodihydrofluorescein diacetate (DCFH-DA) using fluorimeter. Treatment with 200 µM TBHP alone was used as a control for ROS induction. Data presented here are the results of two independent measurements, done in triplicates. One-way ANOVA with Tukey’s *post hoc* test was used for statistical analysis, *p < .05, **p < .01, ***p < .001.

We measured the formation of oxidative stress by-products using 2′,7′-dichlorodihydrofluorescein diacetate (DCFH-DA). The results showed that none of the compounds influenced the basal level of ROS in the cells (Figure S3, Supporting Information). Contrary to this, when oxidative stress was induced, both compounds reduced the ROS levels (Figure 4). However, this reduction was very moderate and not comparable to NAC, which generally confirmed their relatively modest antioxidant capacity obtained in DPPH/FRAP assays.

**Figure 5.**
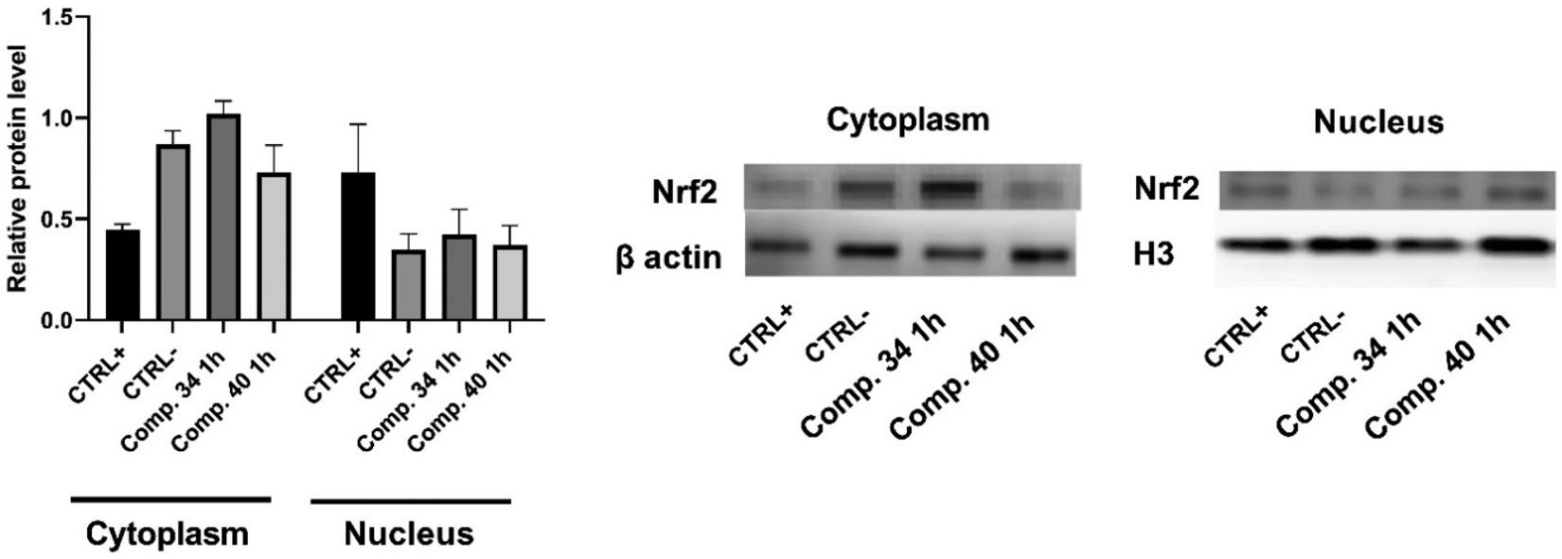
Nrf2 protein levels in the cytoplasmic and the nuclear fraction of HCT116 cells, assessed by Western blot analysis. HCT116 cells were treated with the 4 mM hydrogen peroxide as a positive control for ROS induction and with compounds **34** and **40** (50 µM and 5 µM, respectively) for 1 h. Representative western blots of Nrf2 in cytoplasm and the nuclear fraction are presented. Histone H3 and β-actin were employed as loading controls for nuclear and cytoplasmic fraction; respectively. Results are presented as mean ± SD; number of samples was *n* = 3.

In addition, we evaluated the potential ability of **34** and **40** to translocate Nrf2 to the nucleus in order to assess their capacity to activate the Nrf2 signalling, by Western blot analysis. Nrf2 (NF-E2-related factor 2) is a transcription factor that induces gene expression of antioxidant enzymes and many other cytoprotective enzymes through binding to antioxidant response elements (ARE). Under physiological conditions, the Nrf2 is inactivated by binding to Kelch-like ECH-associated protein1 (Keap1). However, upon a stressful condition, especially under oxidative stress, Nrf2 separates from Keap1 and transfers to the nucleus where it activates ARE and increases transcription of Nrf2-regulated genes, thereby reducing the injury to stress, i.e. downregulates the level of intracellular ROS. Thus, Keap1/Nrf2/ARE signalling pathway is an important target for reducing oxidative stress^34^^,^^35^. As can be seen in Figure 5, in the control/non-treated cells Nrf2 is dominantly expressed in the cytoplasm, while its expression in the nucleus is low. On the other hand, when treated with hydrogen peroxide, its expression is significantly higher in the nucleus. This confirmed its translocation from the cytoplasm to the nucleus, as a mechanism of the antioxidant defense of the cells. Contrary to this, the nuclear expression of Nrf2 in the cells treated with **34** and **40** is similar to the Nrf2 expression in non-treated cells, pointing that **34** and **40** did not promote the translocation of Nrf2 to the nucleus.

This confirms that both compounds did not induce oxidative stress in HCT 116 cells. Also, they did not activate the Nrf2 signalling pathway as a potential mechanism of their activity.

In conclusion, although both compounds demonstrated mild antioxidative activity, the pronounced cytotoxic activity observed upon treatment with **40**, could not be explained by the activation of either oxidative stress, or the Nrf2 signalling pathway.

### 3.3. 3D-Qsar analysis

**Figure 6.**
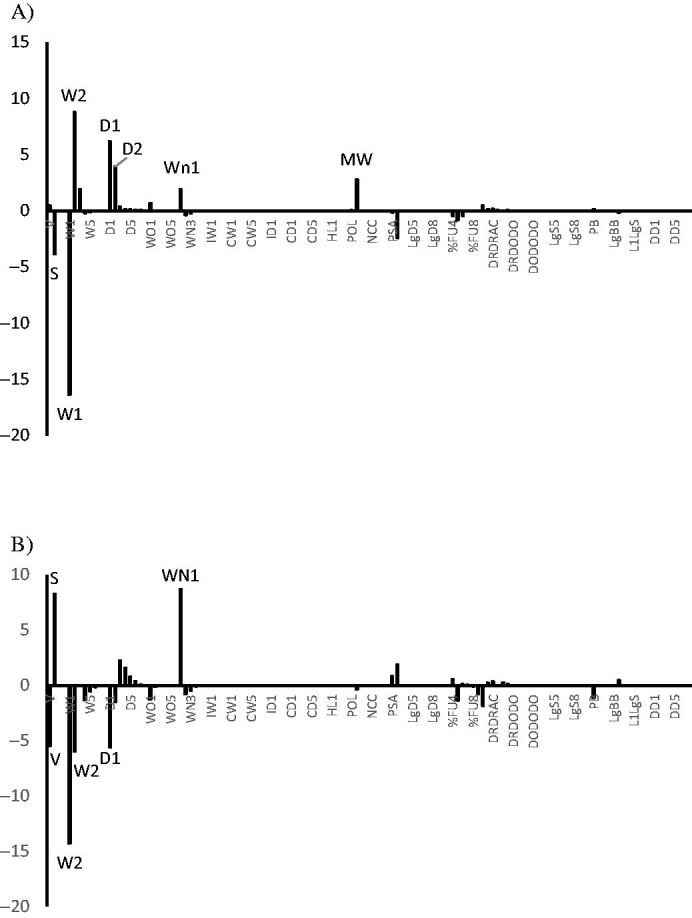
Products of the descriptor’s average value calculated for the dataset used to derive model and the associated PLS coefficient of 3D-QSAR model for: (A) model **1** and (B) model **2**.

In order to identify molecular properties with the highest influence on antioxidative activity of studied compounds, 3D-QSAR analyses were performed. 3D-QSAR models (Table 3 and Figures S1 and S2 in Supporting Information) were generated using antioxidative activities obtained by both tests, DPPH (model **1**) and FRAP (model **2**). Since both models were generated using raw data, to estimate the real impact of each molecular descriptor on the antioxidative activity, it is necessary to inspect the product of the descriptor’s value and its PLS coefficient (Figure 6).

**Table 3.** Statistical properties of 3D-QSAR models.

Model	nO^a^	LV^b^	*R* ^2^	*SDEC* ^c^	*Q* ^2d^	*SDEP* ^e^	*SDEP-ext* ^f^
**1**	23	5	0.83	0.46	0.58	0.73	0.92
**2**	23	6	0.84	0.54	0.51	0.95	0.74

^a^ Number of objects used to build the model.

^b^ Number of latent variables.

^c^ *SDEC* – standard deviation of error of calculation.

^d^ *Q*^2^ is the cross-validated predictive performance.

^e^ *SDEP* – standard deviation of error of prediction obtained by cross-validation.

^f^ *SDEP-ext* – standard deviation of error of prediction obtained by external validation.

Such analysis pointed to different molecular properties as the most important ones for the antioxidative activity measured by different test. In case of DPPH test, possibility of a compound to accept hydrogen bonds (WN1), hydrophobic regions (D1 and D2) related to the polarizability and dispersion forces and molecular mass (MW) were identified as positively correlated with antioxidative activity, while surface (S) was identified as negatively correlated. Hydrophilic regions calculated at different energy levels (W1 and W2) had opposite influence on antioxidative activity measured by DPPH test. In case of FRAP test, possibility of a compound to accept hydrogen bonds (WN1) and surface (S) were positive correlated, while volume (V), hydrophilic (W1) and hydrophobic regions (D1 and D2) related to the polarizability and dispersion forces were negative correlated with antioxidative activity.

The same descriptors that were identified by the PLS analyses were also identified by the PCA as the ones with the largest variation among the compounds (Figure S3 in Supporting Information). Possibility of a compound to accept H-bond was the only descriptor that showed the same, positive, impact on antioxidative activity for both tests^36^^,^^37^.

## 4. Conclusions

Within this manuscript, we described the design, synthesis, QSAR analysis and biological activity of novel *N*-substituted benzimidazole derived carboxamides prepared as potential antioxidants with antiproliferative activity. All designed derivatives were synthesised using classical and microwave-assisted synthesis. The targeted carboxamides were designed to study the impact of the number of methoxy groups, the type of the substituent placed at the N atom and at the 5(6)-position of the benzimidazole cores on biological activity. Their antioxidative activity was studied by DPPH and FRAPS assays, while antiproliferative activity was determined on three human cancer cells and embryonic kidney cells. In addition, their pro- and antioxidant activity was measured in tumour cells.

The results obtained from the determination of antioxidative activity revealed that the most promising derivatives were unsubstituted derivative **28** (IC_50_ 3.78 mM, 538.81 mmolFe^2+^/mmolC) and dimethoxy derivative **34** (IC_50_ 5.68 mM, 618.10 mmolFe^2+^/mmolC) both bearing methyl group at the N atom of benzimidazole core, which was chosen for further optimisation. All other tested compounds showed moderate to weak activity. On the other hand, the antioxidant activity in the cells was rather modest.

The 3D-QSAR analysis identified possibility of a compound to accept hydrogen bond as positively correlated property with the antioxidative activity measured by both tests, DPPH and FRAP.

Importantly, several compounds demonstrated prominent activity against cancer cells, being significantly less cytotoxic towards non-tumour cell line HEK 293. The most active and selective compounds, trimethoxy substituted derivative **43** and unsubstituted derivative **40** both bearing isobutyl side chain at N atom and cyano group on benzimidazole core, showed the most pronounced activity against HCT116 (IC_50_ ≈ 0.6 µM; both) and H 460 cells (IC_50_ ≈ 0.4 µM, IC_50_ ≈ 2.5 µM; respectively). The analysis of the Nrf2 expression point to the fact that the pronounced cytotoxic activity of selected compounds could not be explained by the activation of either oxidative stress, or the Nrf2 signalling pathway.

## Supplementary Material

Supplemental Material
