# Supplementary material for: Design, synthesis, biological evaluation and QSAR analysis of novel N-substituted benzimidazole derived carboxamides
Source: J Enzyme Inhib Med Chem. 2022 May 6;37(1):1327–39. doi: 10.1080/14756366.2022.2070910 (PMC9090388; doi:10.1080/14756366.2022.2070910)
Supplement: Supplemental Material [file IENZ_A_2070910_SM2546.pdf]

Synthesis, QSAR analysis and biological activity of novel *N*-substituted benzimidazole derived carboxamides as potential antioxidants with antiproliferative activity

Anja Beč[1], Marija Mioč[2], Branimir Bertoša[3], Marija Kos[1], Patricia Debogović[1], Marijeta Kralj[2], Kristina Starčević[4]* and Marijana Hranjec[1]*

Content:

1. **Figure S1:** Predicted *vs* experimental antioxidative activity expressed as pIC_{50} – negative logarithmic value of concentration that causes 50% of antioxidative activity measured: A) using DPPH test (model **1**) and B) using FRAP test (model **2**).
2. **Figure S2:** PCA loadings plot. Molecular descriptors with the highest contributions to the first two principal components are labelled. The first two principal components explained 91 % of variance of the descriptors (X-matrix).
3. **Figure S3.** Impact of compounds on cellular ROS production was measured with fluorescent dye DCF-DA in HCT116 cell line, after the treatment with 10μM compounds for 1h.
4. **Figures S3-S85:** NMR spectra of synthesized compounds

A)

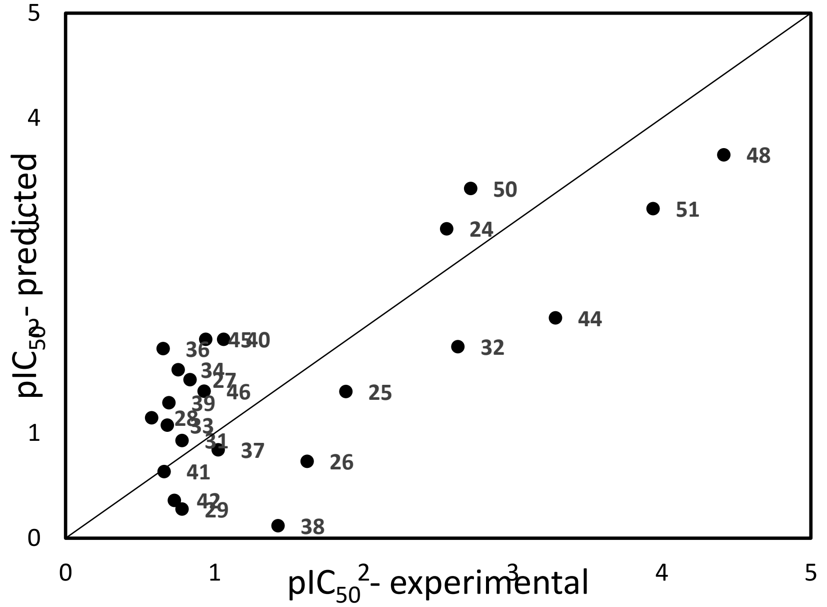

B)

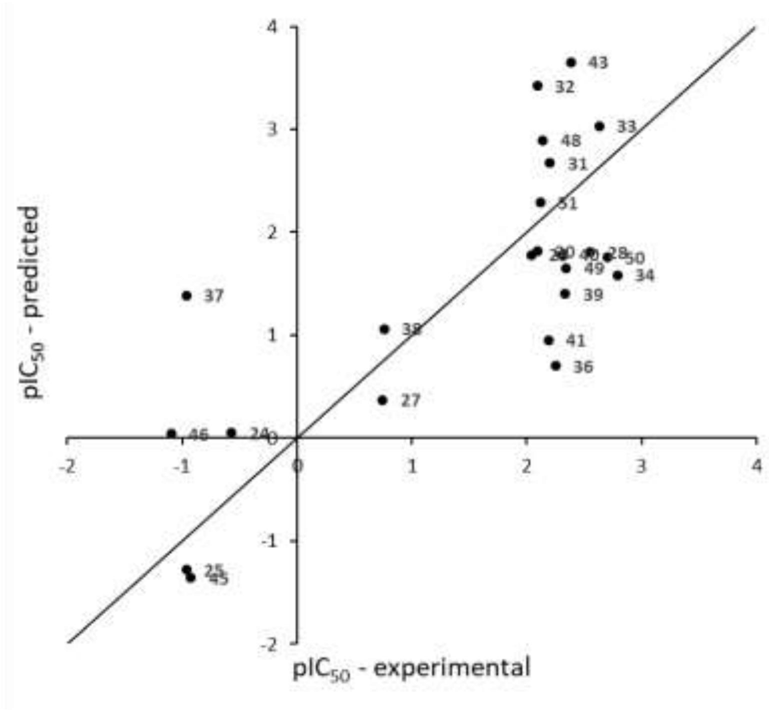

Figure S1. Predicted *vs* experimental antioxidative activity expressed as pIC_{50} – negative logarithmic value of concentration that causes 50% of antioxidative activity measured: A) using DPPH test (model **1**) and B) using FRAP test (model **2**).

A)

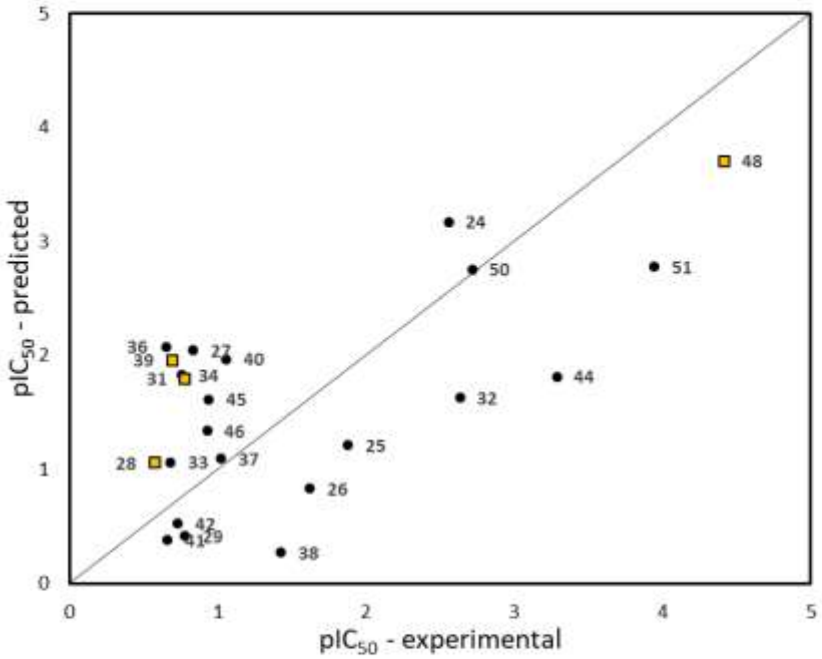

B)

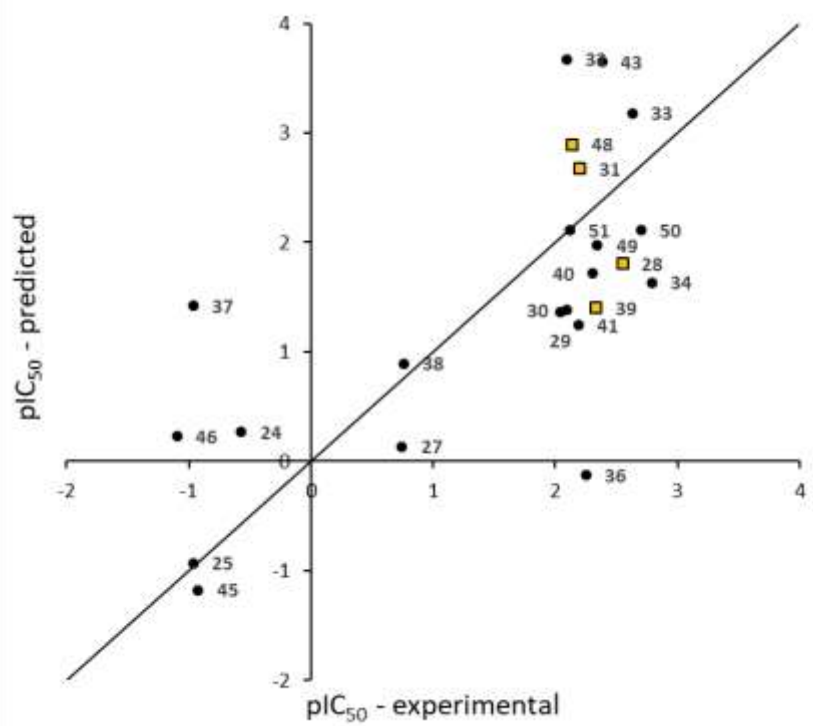

Figure S2. External predictions of the model obtained using: A) DPPH test and B) using FRAP test. Test set compounds: **28**, **31**, **39**, and **48** are marked as yellow squares. Training set consisted of the remaining compounds of the dataset used to generate the model.

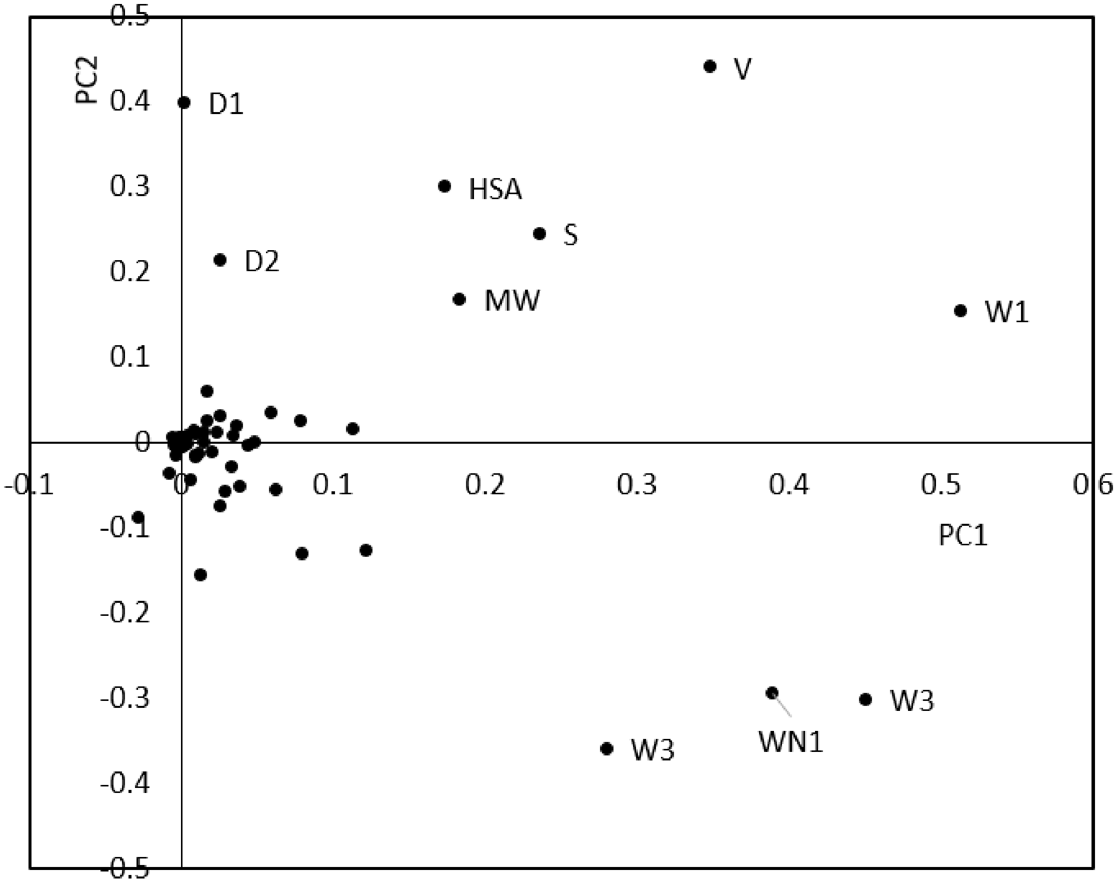

Figure S3. PCA loadings plot. Molecular descriptors with the highest contributions to the first two principal components are labelled. The first two principal components explained 91 % of variance of the descriptors (X-matrix).

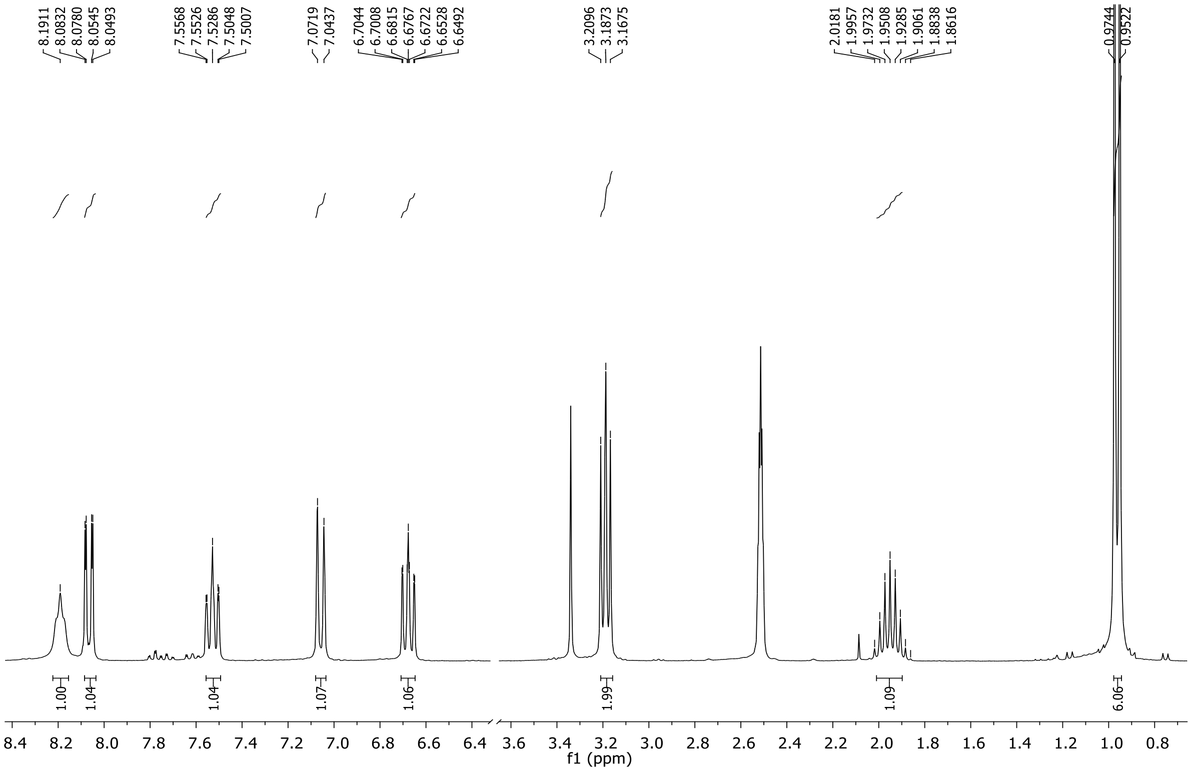

Figure S4. ^{1}H NMR spectrum (DMSO-d_6, 300 MHz) of *N-isobutyl-2-nitroaniline* ***3***

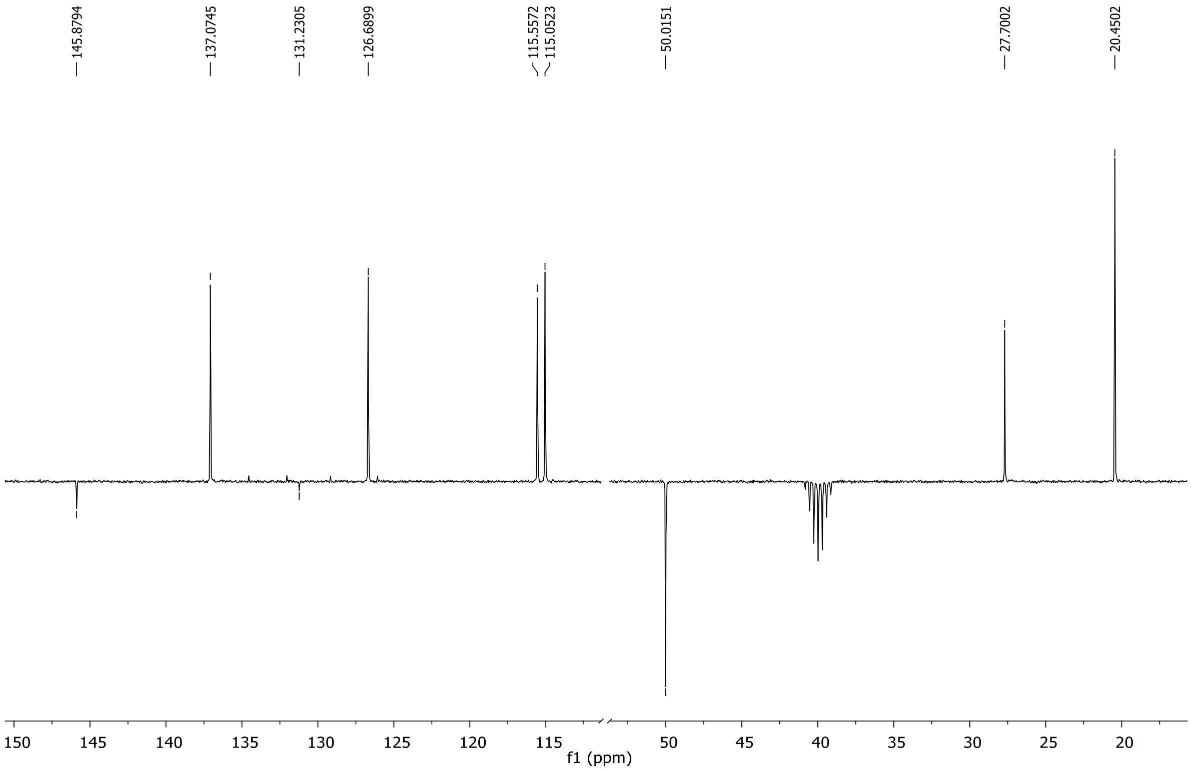

Figure S5. ^{13}C NMR spectrum (DMSO-d_6, 75 MHz) of *N-isobutyl-2-nitroaniline* ***3***

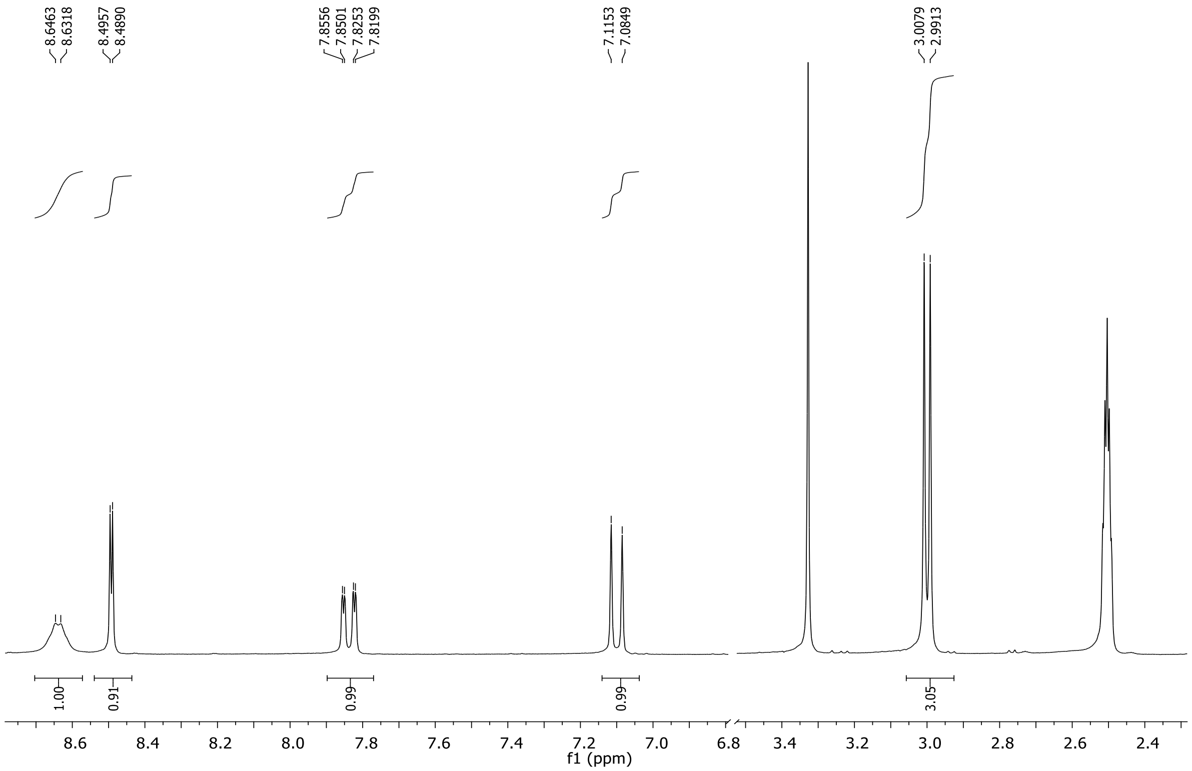

Figure S6. ^{1}H NMR spectrum (DMSO-d_6, 300 MHz) of *3-N-(methylamino)-4-nitrobenzonitrile* ***4***

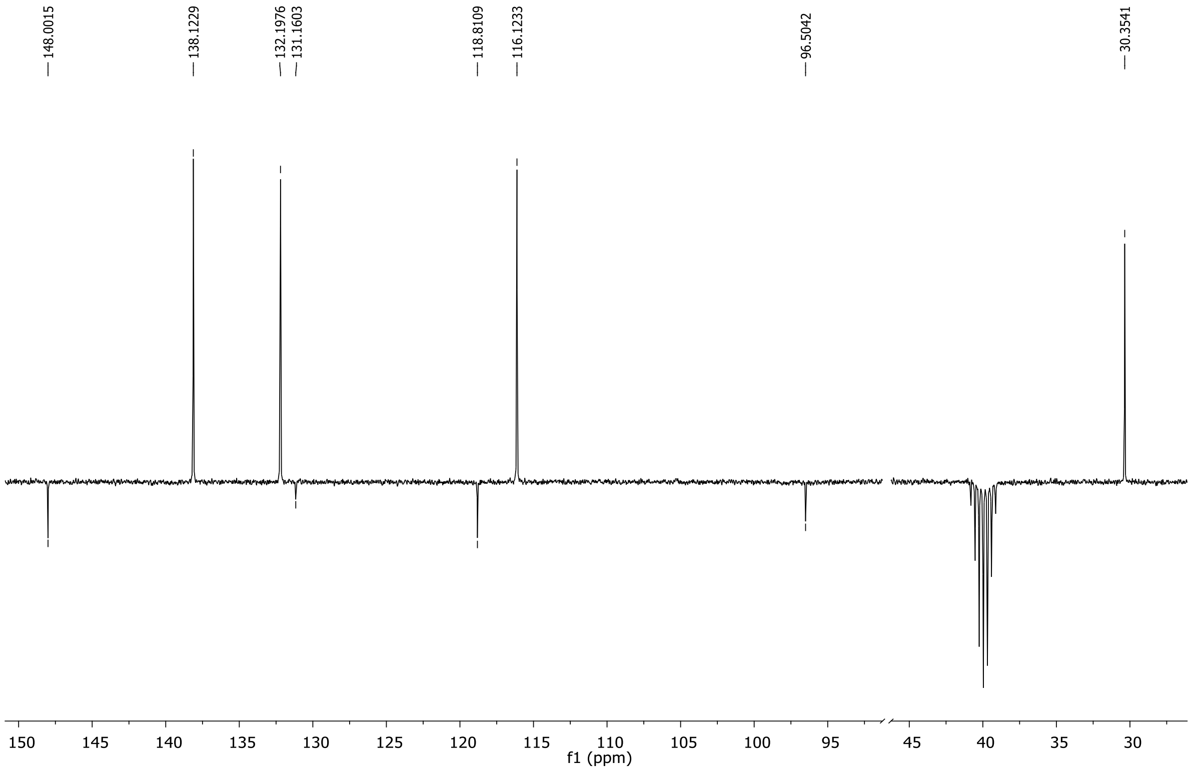

Figure S7. ^{13}C NMR spectrum (DMSO-d_6, 75 MHz) of *3-N-(methylamino)-4-nitrobenzonitrile* ***4***

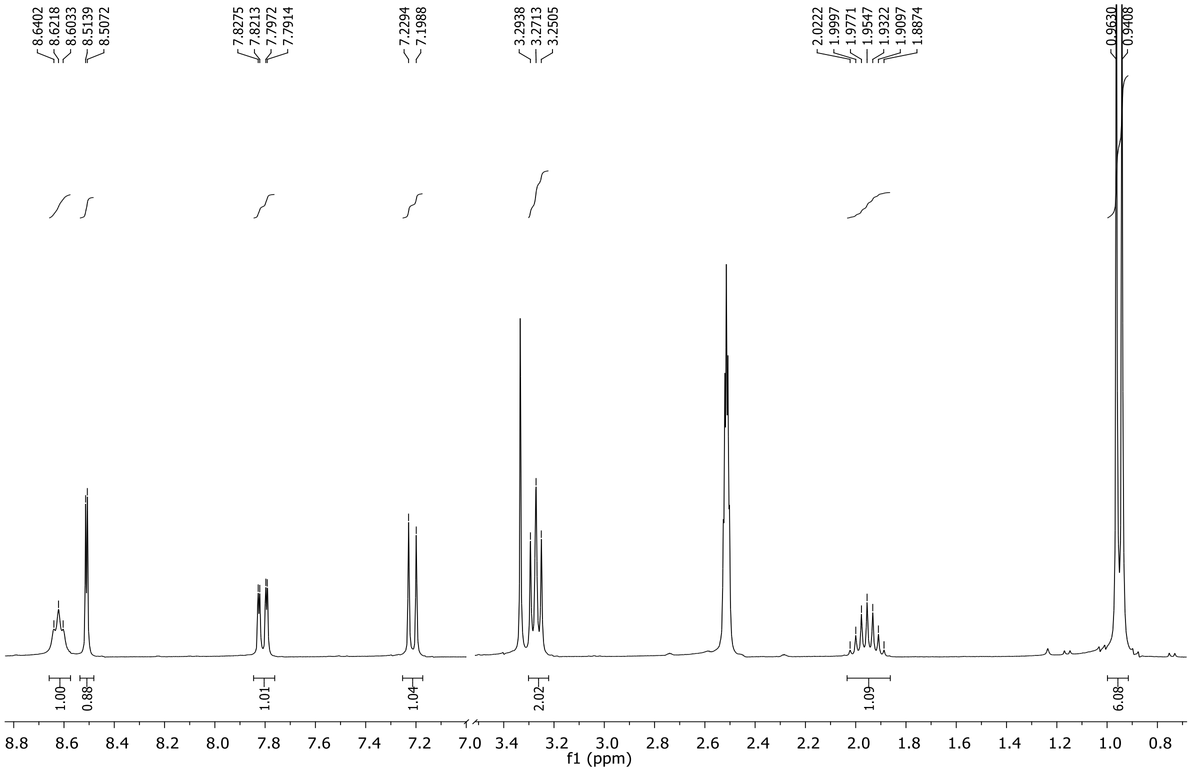

Figure S8. ^{1}H NMR spectrum (DMSO-d_6, 300 MHz) of *3-N-(isobutylamino)-4-nitrobenzonitrile **5***

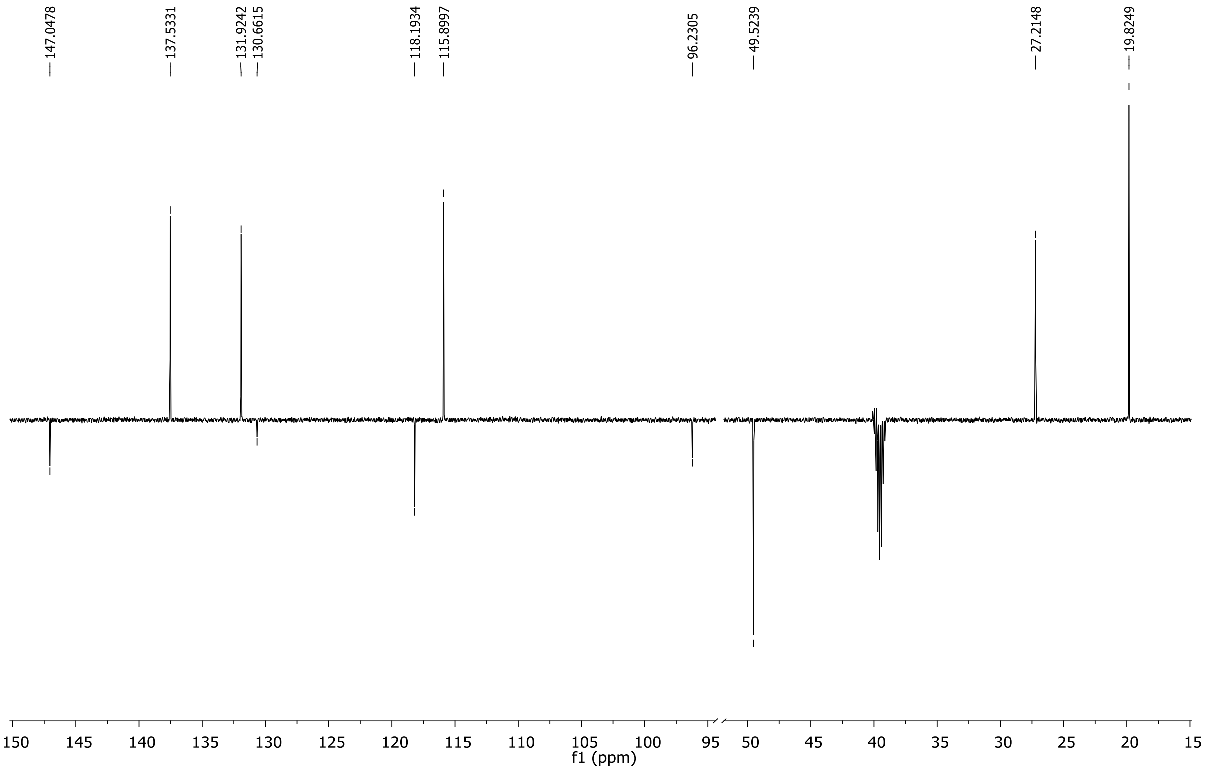

Figure S9. ^{13}C NMR spectrum (DMSO-d_6, 151 MHz) of *3-N-(isobutylamino)-4-nitrobenzonitrile **5***

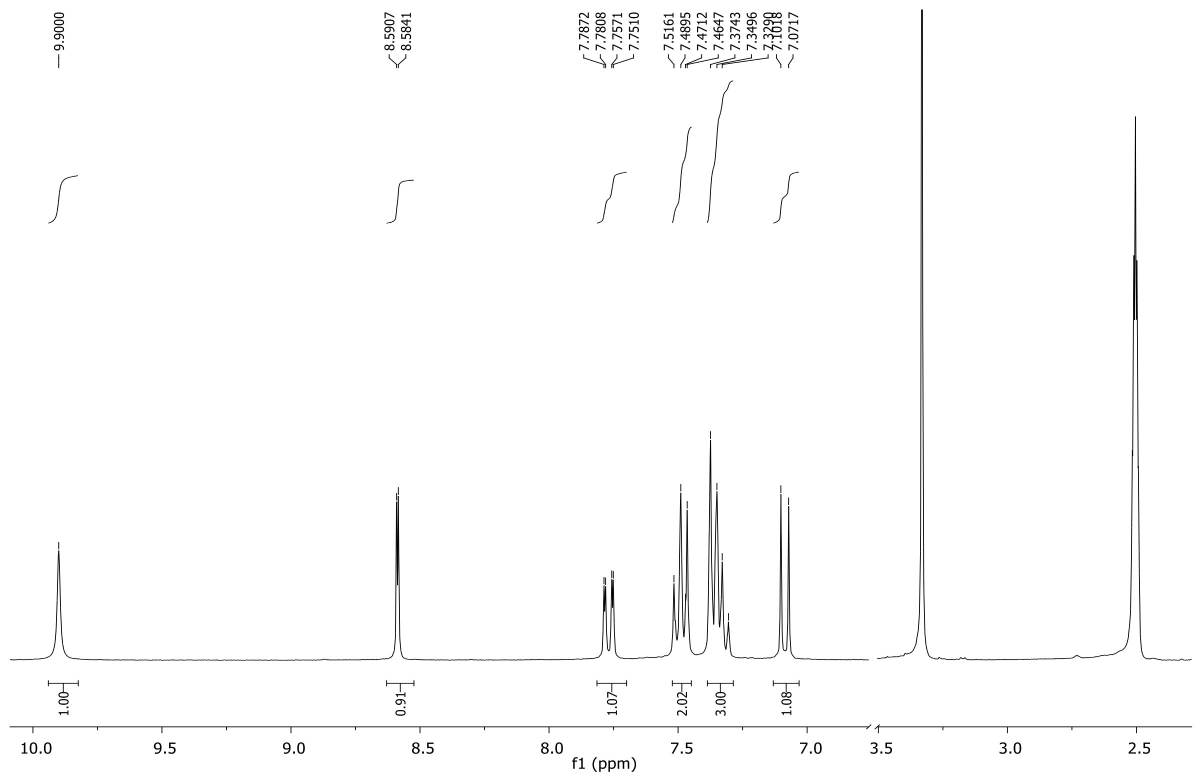

Figure S10. ^{1}H NMR spectrum (DMSO-d_6, 300 MHz) of *4-nitro-3-N-(phenylamino)benzonitrile* ***6***

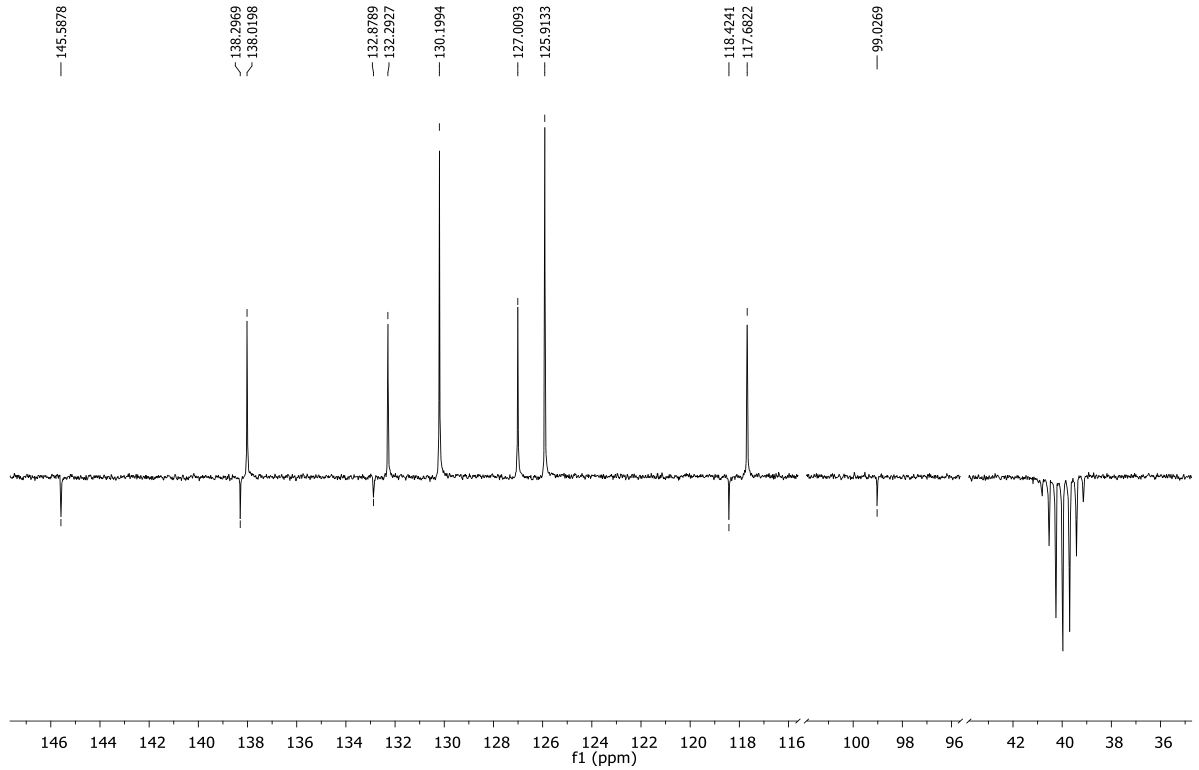

Figure S11. ^{13}C NMR spectrum (DMSO-d_6, 75 MHz) of *4-nitro-3-N-(phenylamino)benzonitrile* ***6***

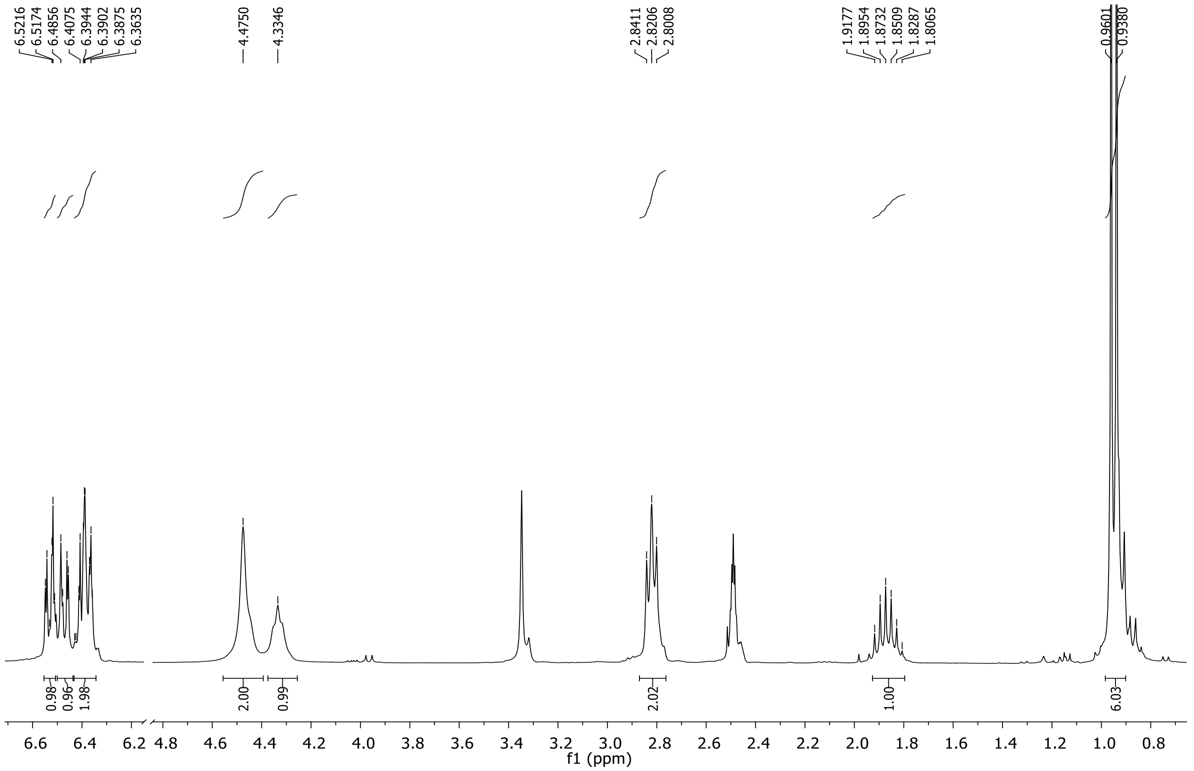

Figure S12. ^{1}H NMR spectrum (DMSO-d_6, 300 MHz) of *N-isobutylbenzene-1,2-diamine* ***7***

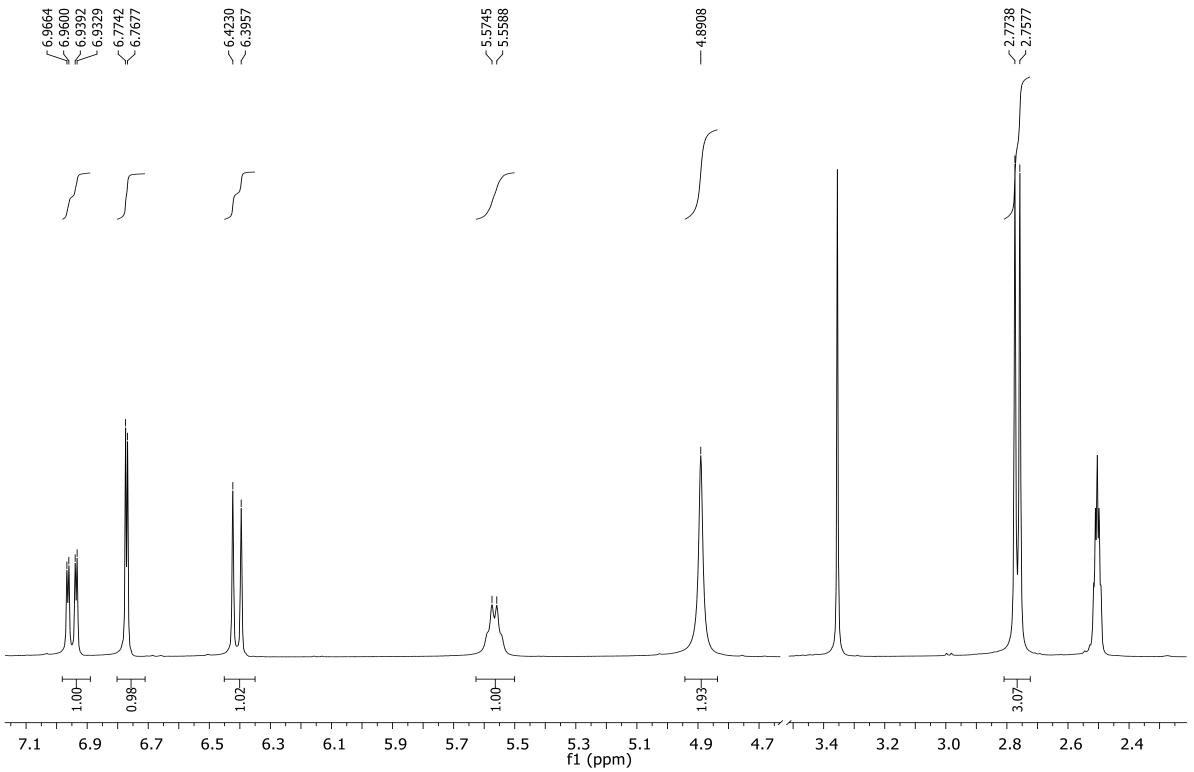

Figure S13. ^{1}H NMR spectrum (DMSO-d_6, 300 MHz) of *4-amino-3-(methylamino)benzonitrile* ***8***

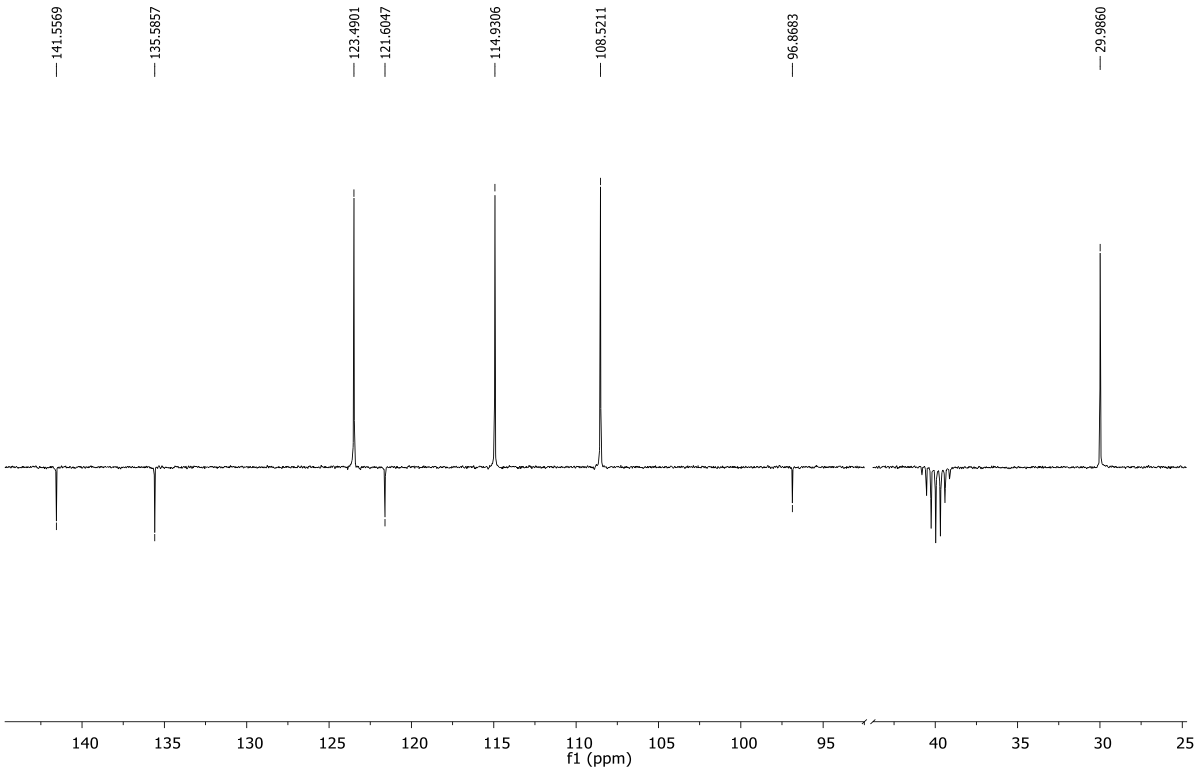

Figure S14. ^{13}C NMR spectrum (DMSO-d_6, 75 MHz) of *4-amino-3-(methylamino)benzonitrile* ***8***

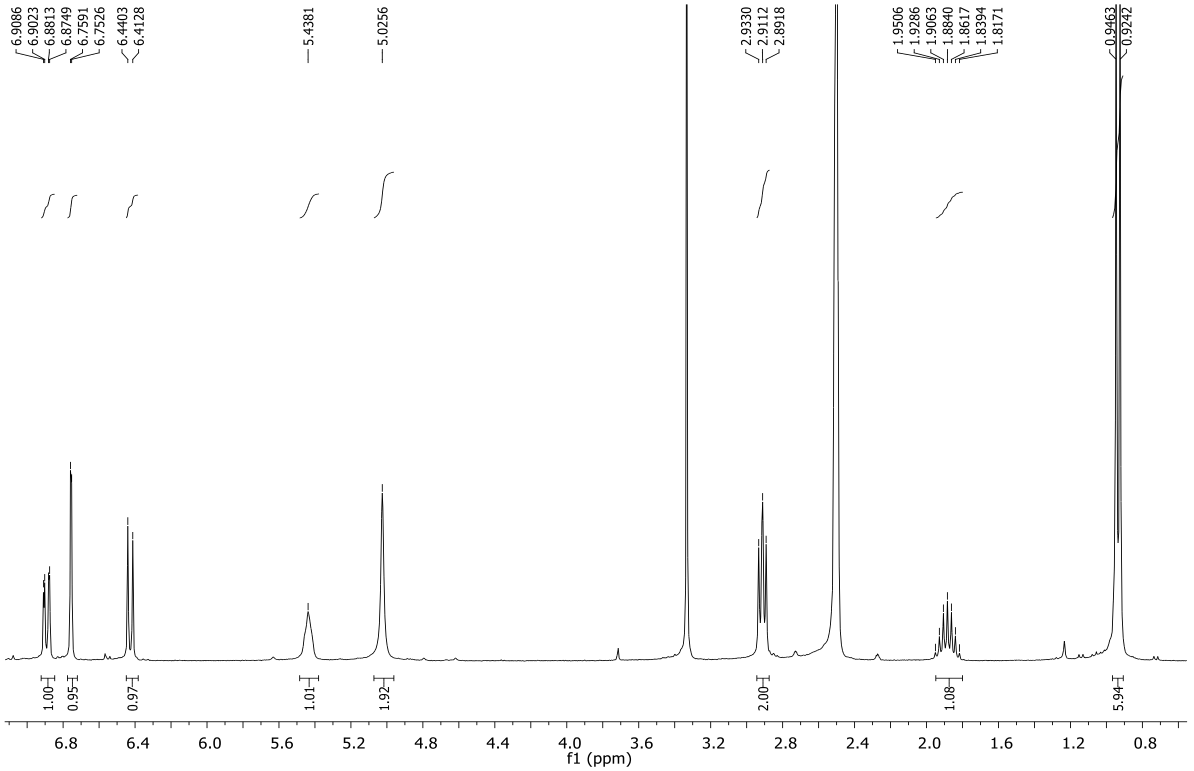

Figure S15. ^{1}H NMR spectrum (DMSO-d_6, 300 MHz) of *4-amino-3-(isobutylamino)benzonitrile* ***9***

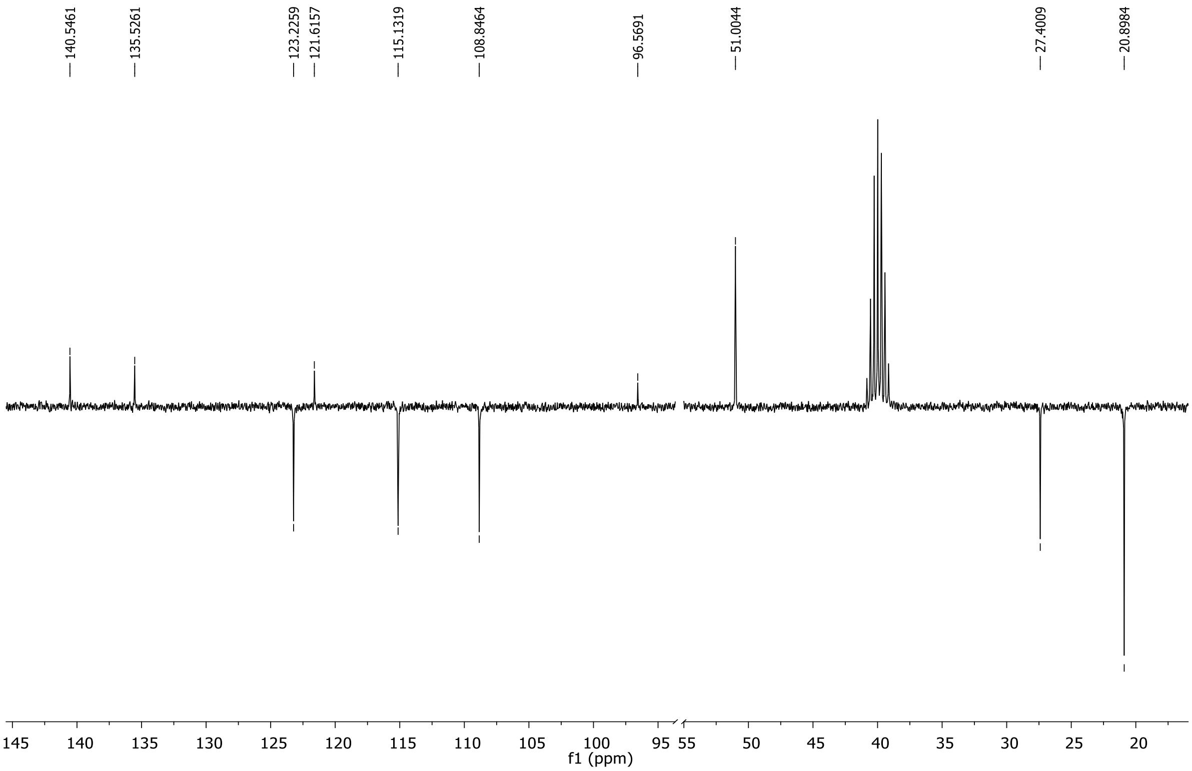

Figure S16. ^{13}C NMR spectrum (DMSO-d_6, 75 MHz) of *4-amino-3-(isobutylamino)benzonitrile* ***9***

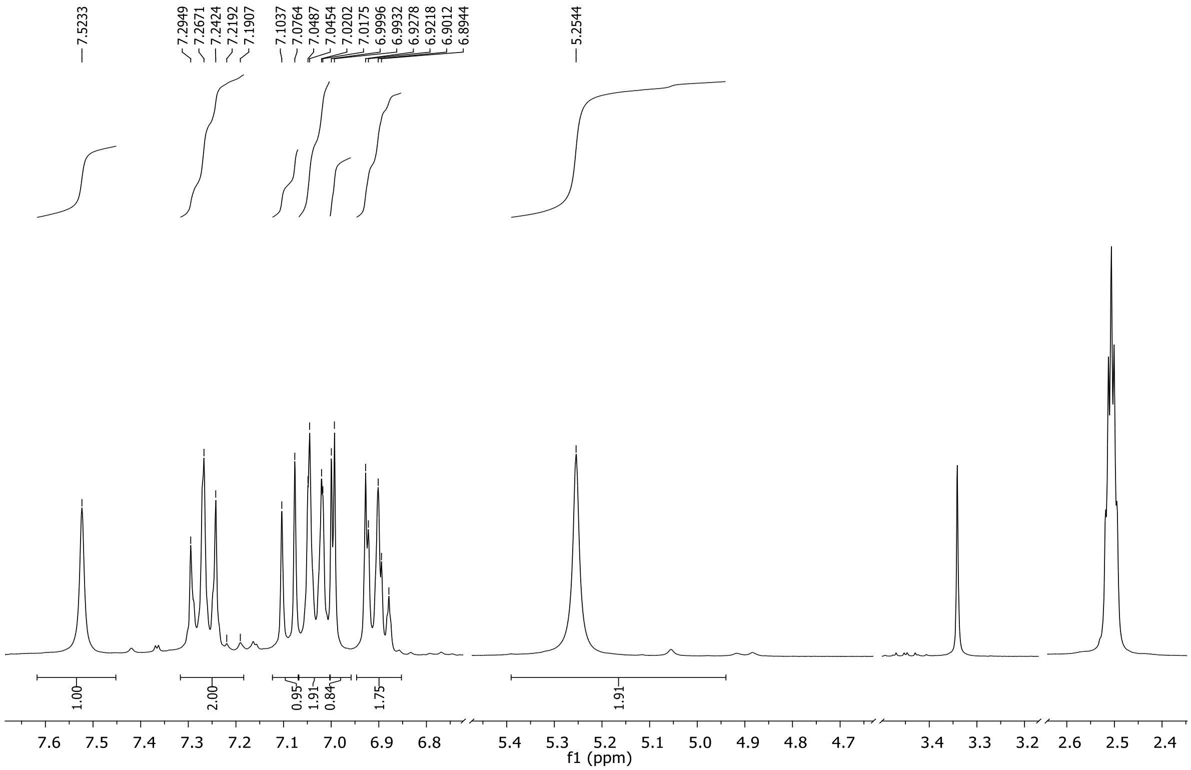

Figure S17. ^{1}H NMR spectrum (DMSO-d_6, 300 MHz) of *4-amino-3-(phenylamino)benzonitrile* ***10***

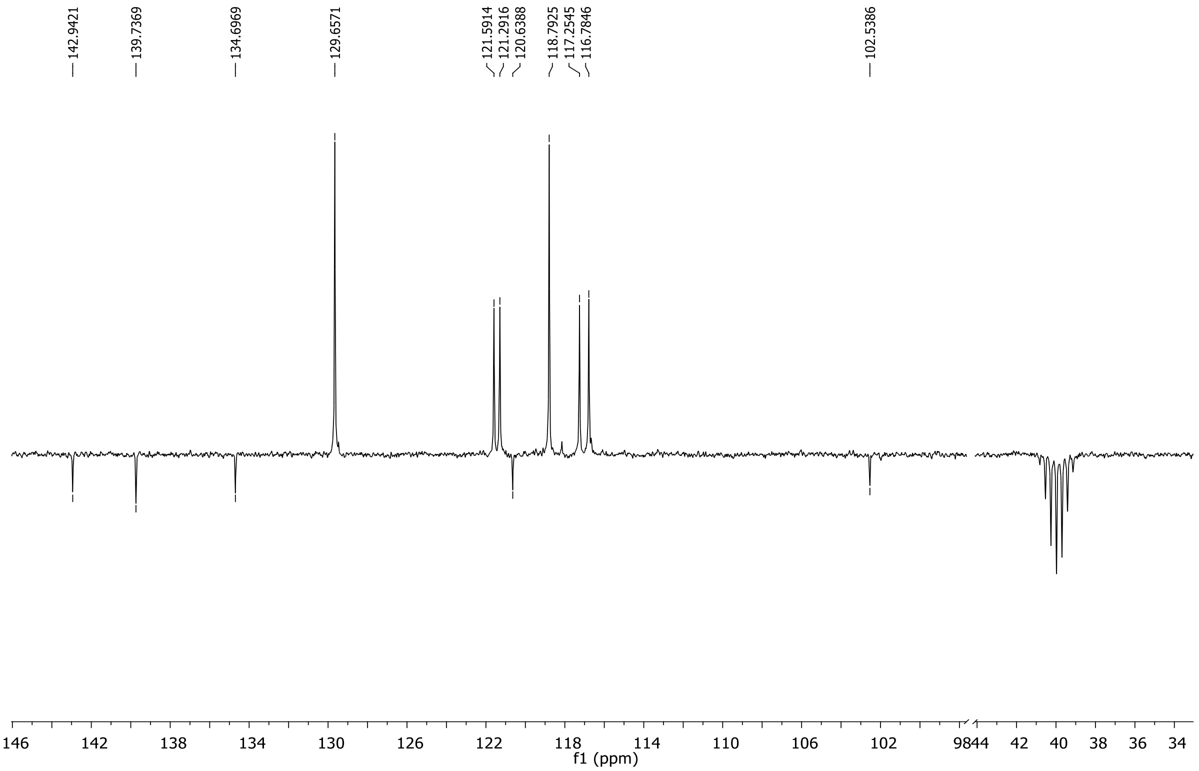

Figure S18. ^{13}C NMR spectrum (DMSO-d_6, 75 MHz) of *4-amino-3-(phenylamino)benzonitrile* ***10***

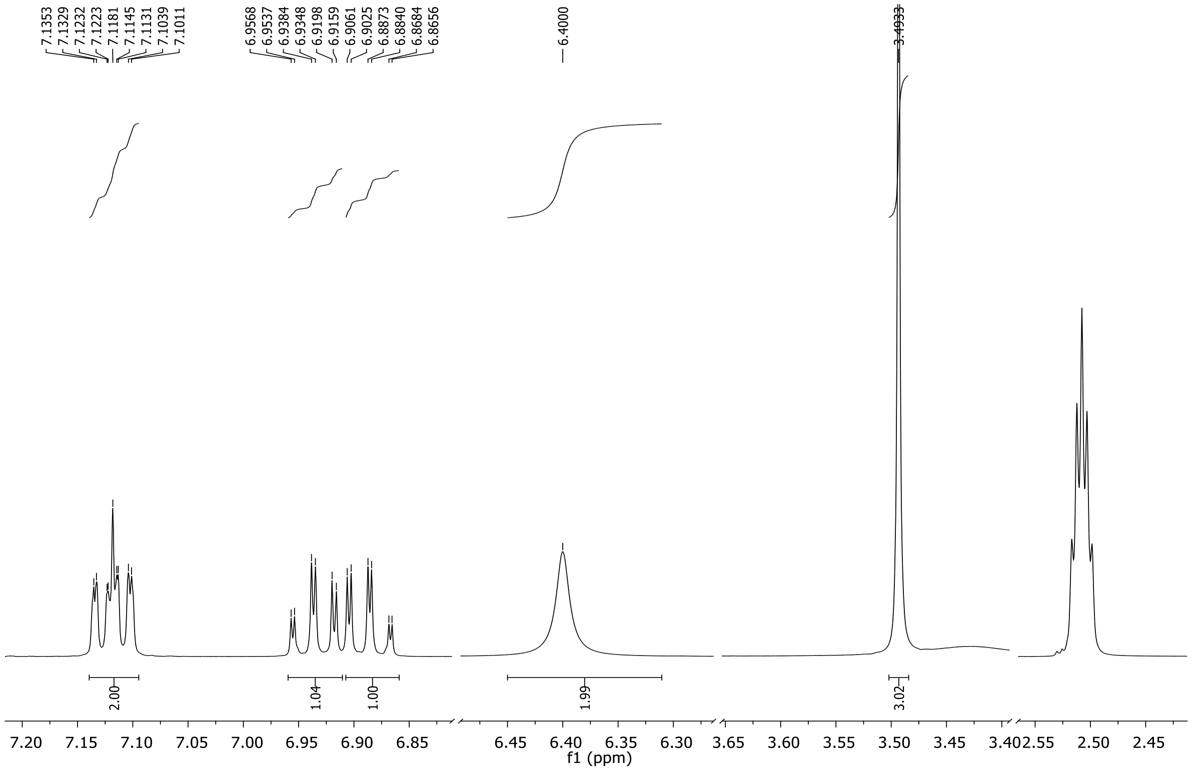

Figure S19. ^{1}H NMR spectrum (DMSO-d_6, 400 MHz) of *2-amino-1-methylbenzimidazole* ***13***

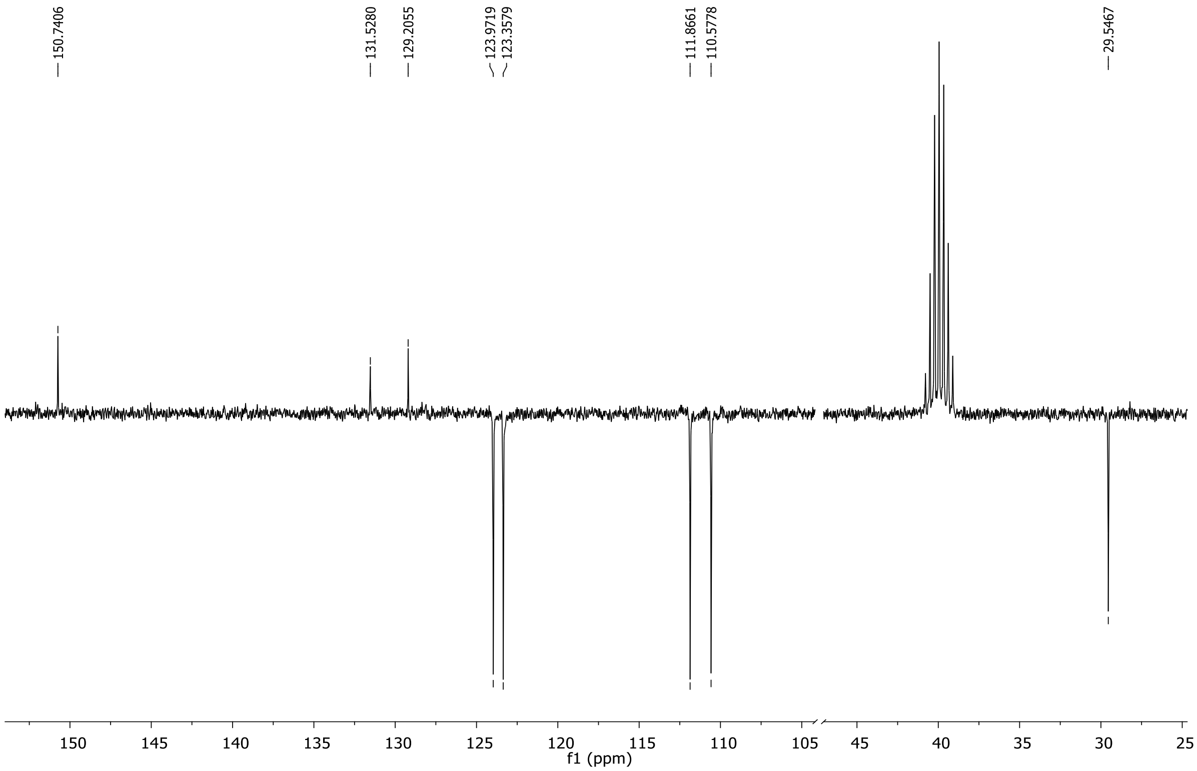

Figure S20. ^{13}C NMR spectrum (DMSO-d_6, 75 MHz) of *2-amino-1-methylbenzimidazole* ***13***

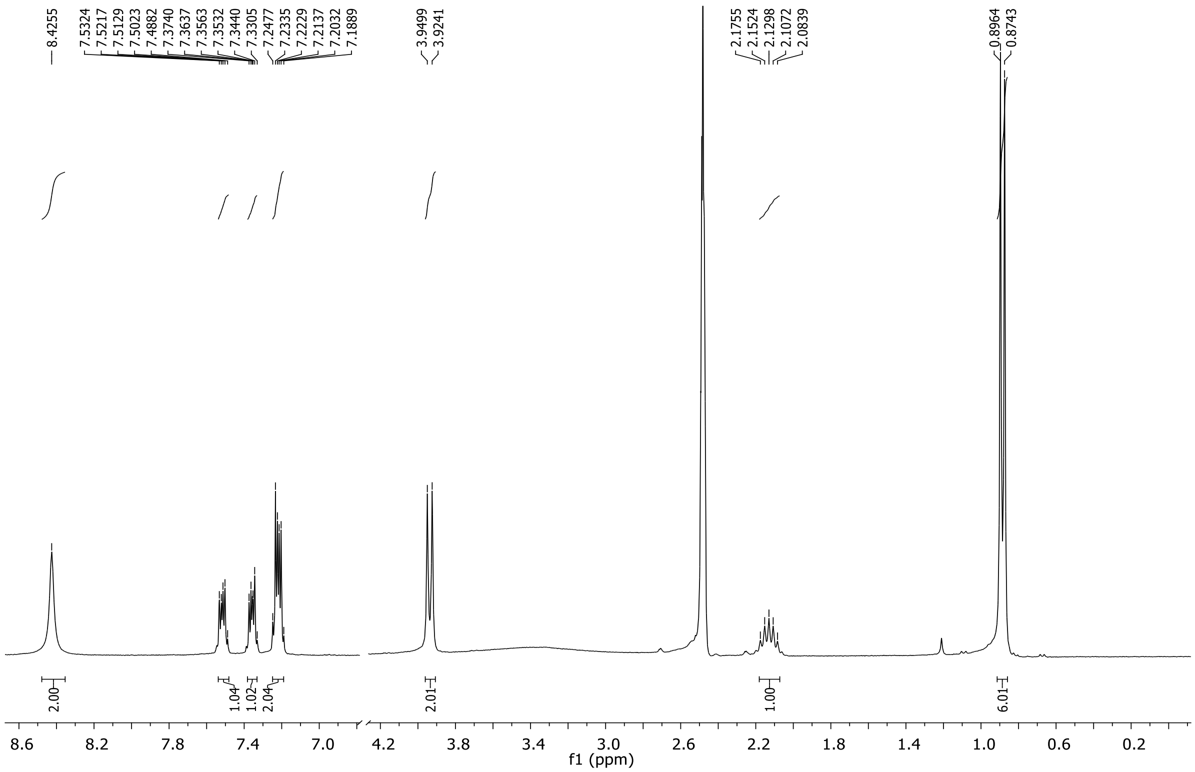

Figure S21. ^{1}H NMR spectrum (DMSO-d_6, 300 MHz) of *2-amino-1-isobutylbenzimidazole* ***14***

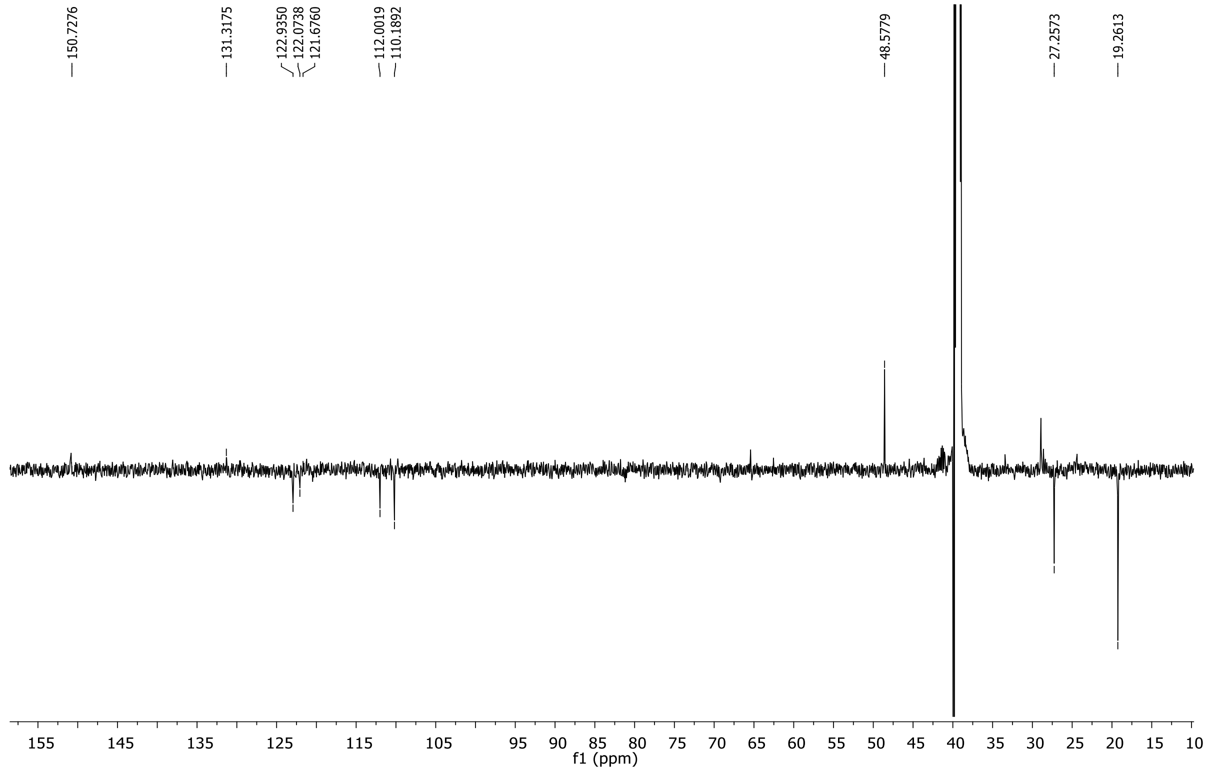

Figure S22. ^{13}C NMR spectrum (DMSO-d_6, 151 MHz) of *2-amino-1-isobutylbenzimidazole* ***14***

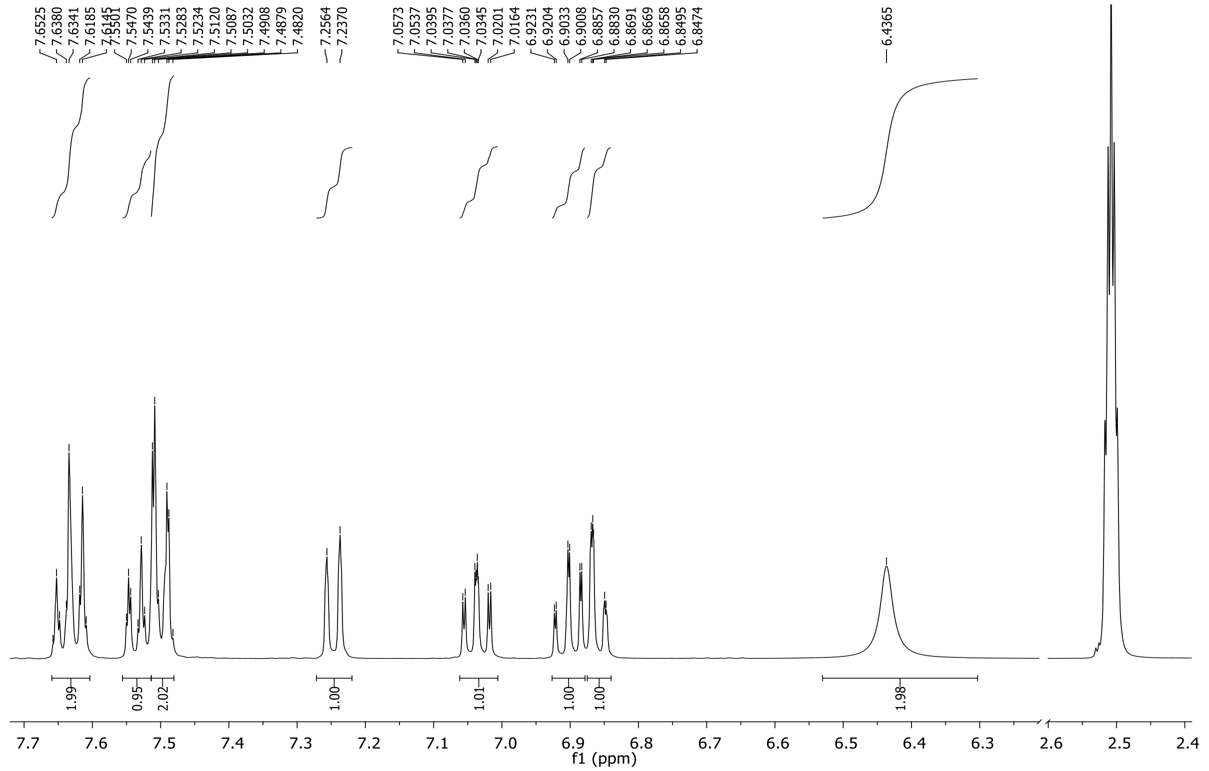

Figure S23. ^{1}H NMR spectrum (DMSO-d_6, 400 MHz) of *2-amino-1-phenylbenzimidazole* ***15***

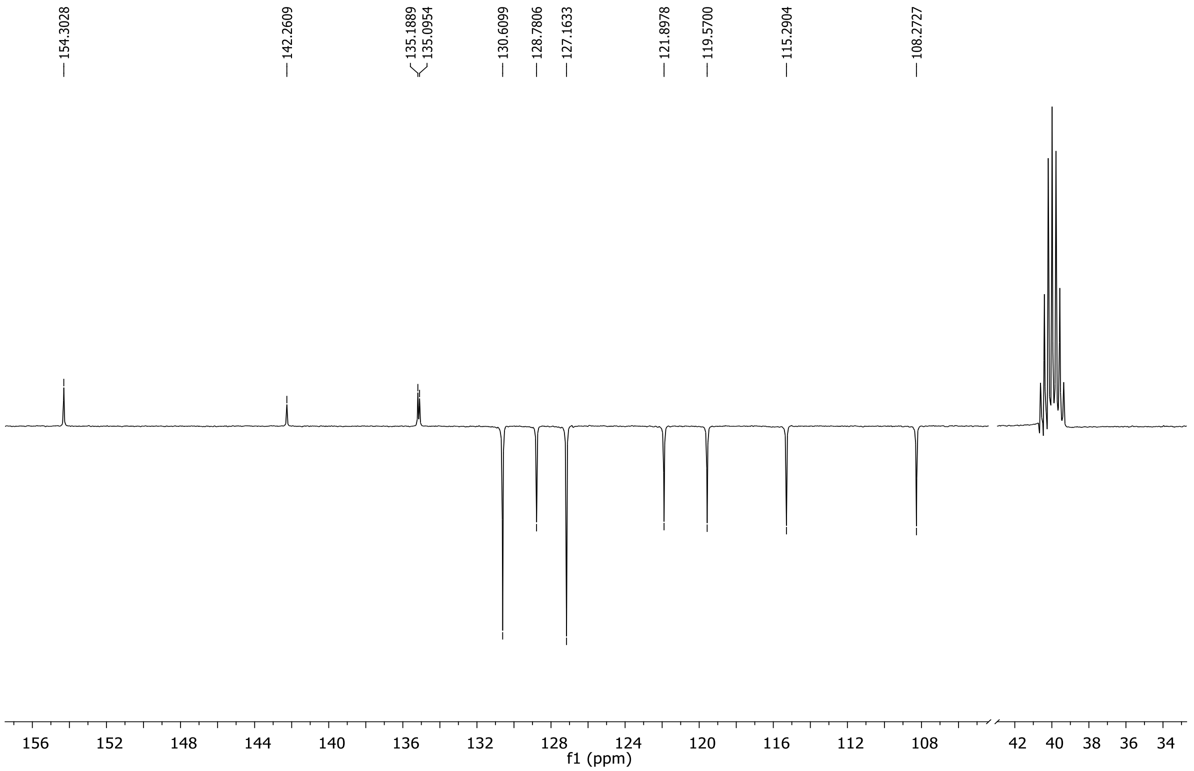

Figure S24. ^{13}C NMR spectrum (DMSO-d_6, 101 MHz) of *2-amino-1-phenylbenzimidazole* ***15***

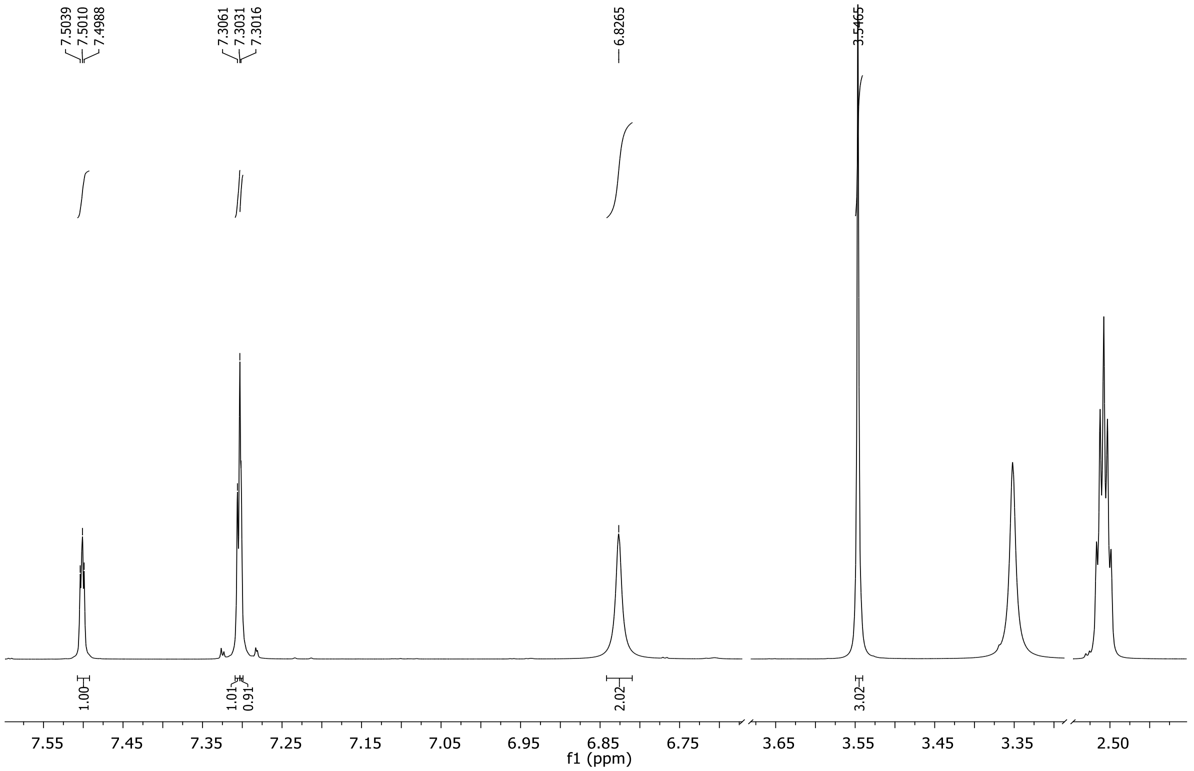

Figure S25. ^{1}H NMR spectrum (DMSO-d_6, 400 MHz) of *2-amino-6-cyano-1-methylbenzimidazole* ***16***

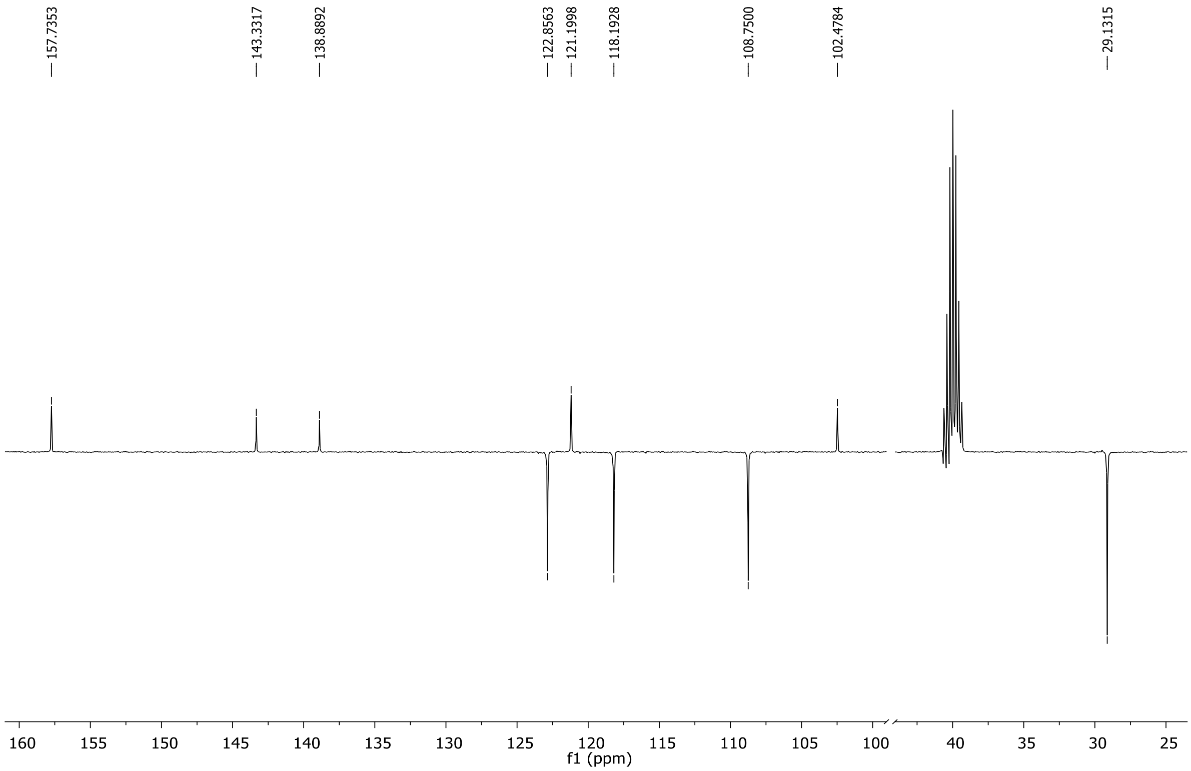

Figure S26. ^{13}C NMR spectrum (DMSO-d_6, 101 MHz) of *2-amino-6-cyano-1-methylbenzimidazole* ***16***

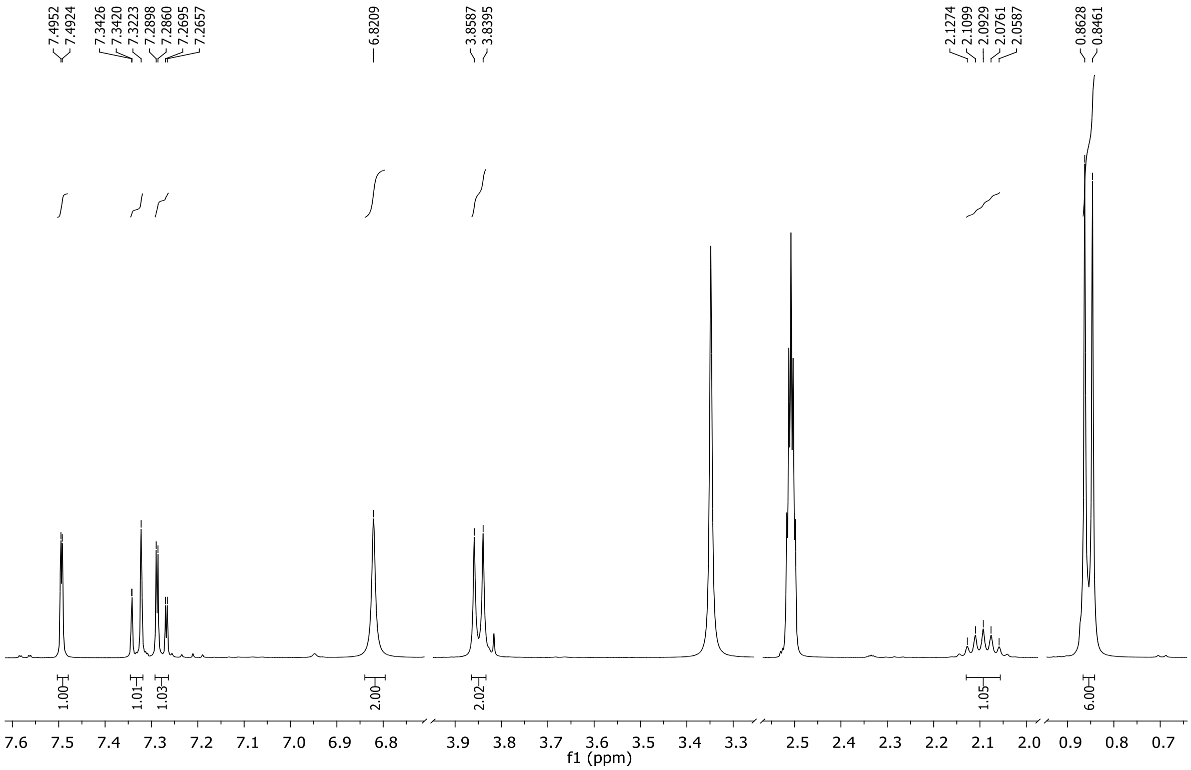

Figure S27. ^{1}H NMR spectrum (DMSO-d_6, 400 MHz) of *2-amino-6-cyano-1-isobutylbenzimidazole* ***17***

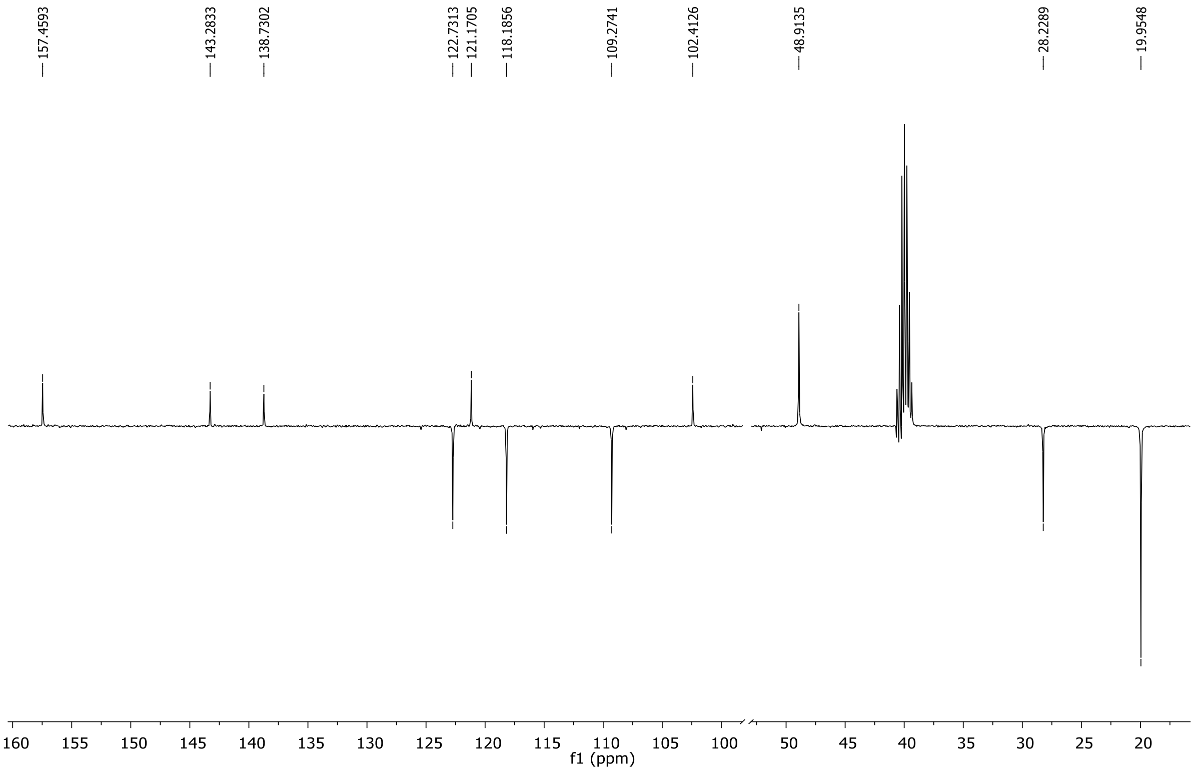

Figure S28. ^{13}C NMR spectrum (DMSO-d_6, 101 MHz) of *2-amino-6-cyano-1-isobutylbenzimidazole **17***

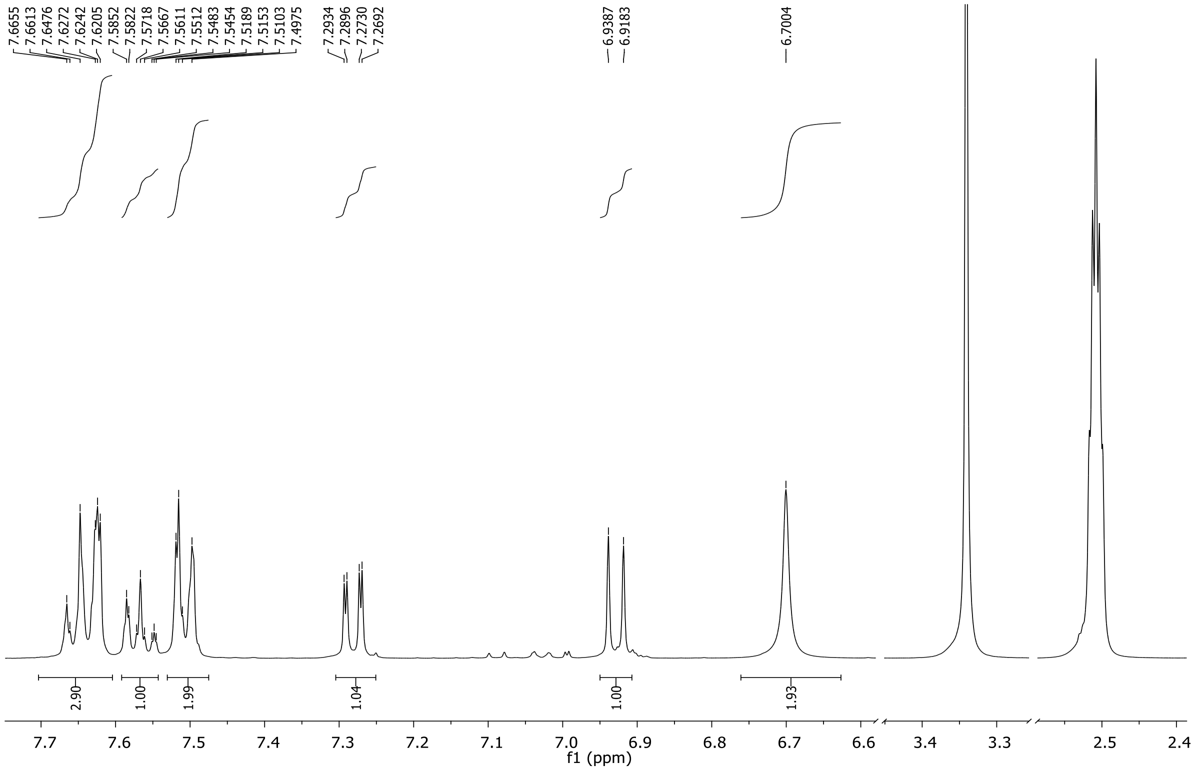

Figure S29. ^{1}H NMR spectrum (DMSO-d_6, 400 MHz) of *2-amino-6-cyano-1-phenylbenzimidazole **18***

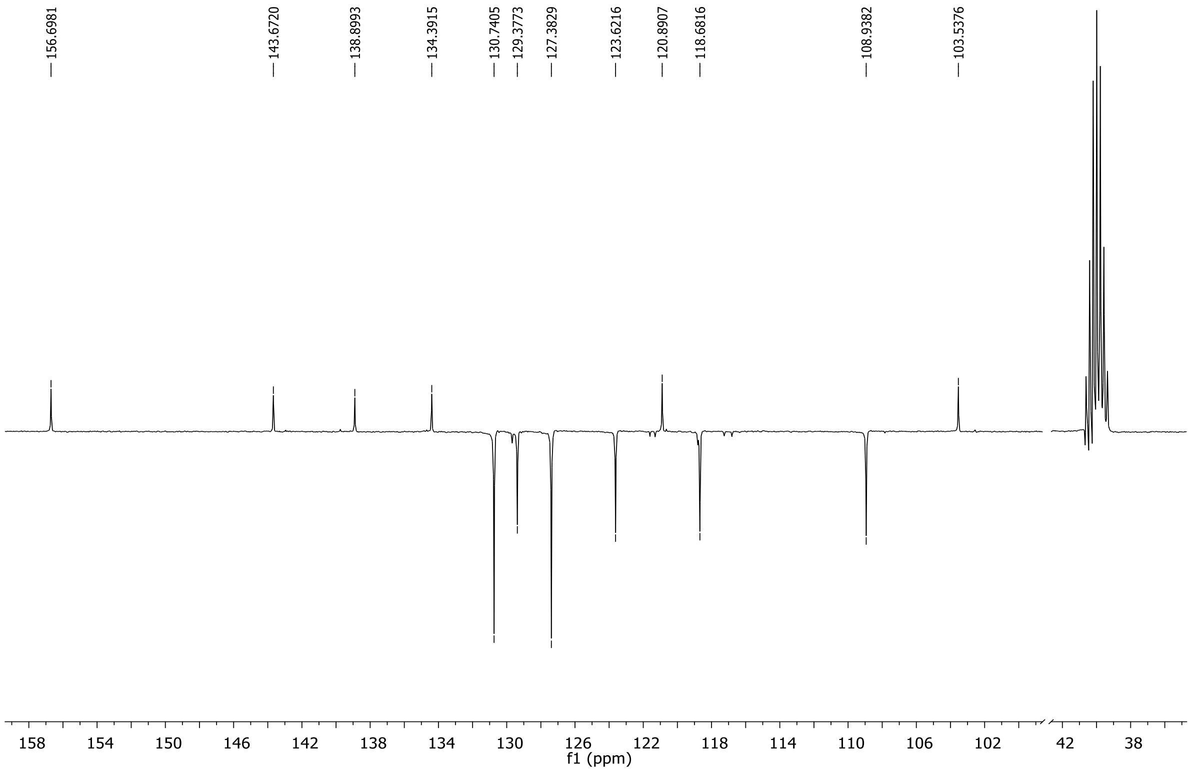

Figure S30. ^{13}C NMR spectrum (DMSO-d_6, 101 MHz) of *2-amino-6-cyano-1-phenylbenzimidazole* ***18***

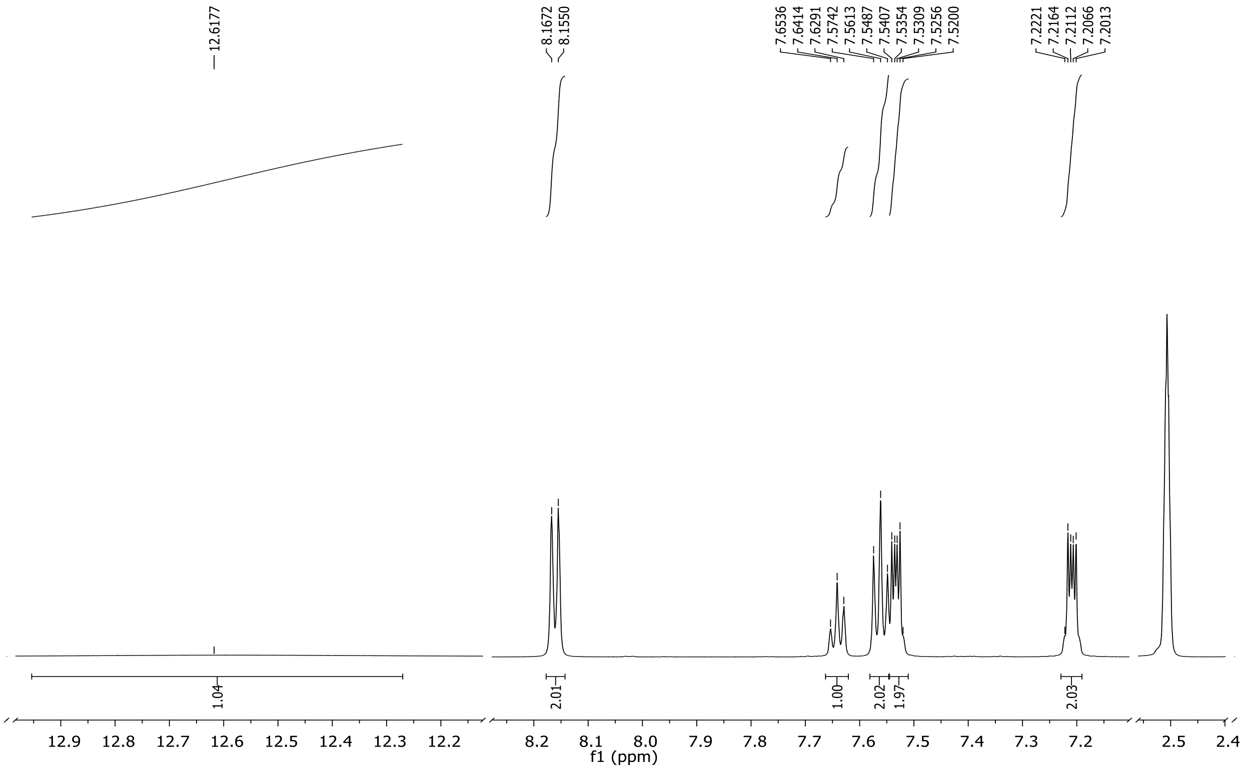

Figure S31. ^{1}H NMR spectrum (DMSO-d_6, 600 MHz) of *N-(1H-benzo[d]imidazol-2-yl)benzamide* ***24***

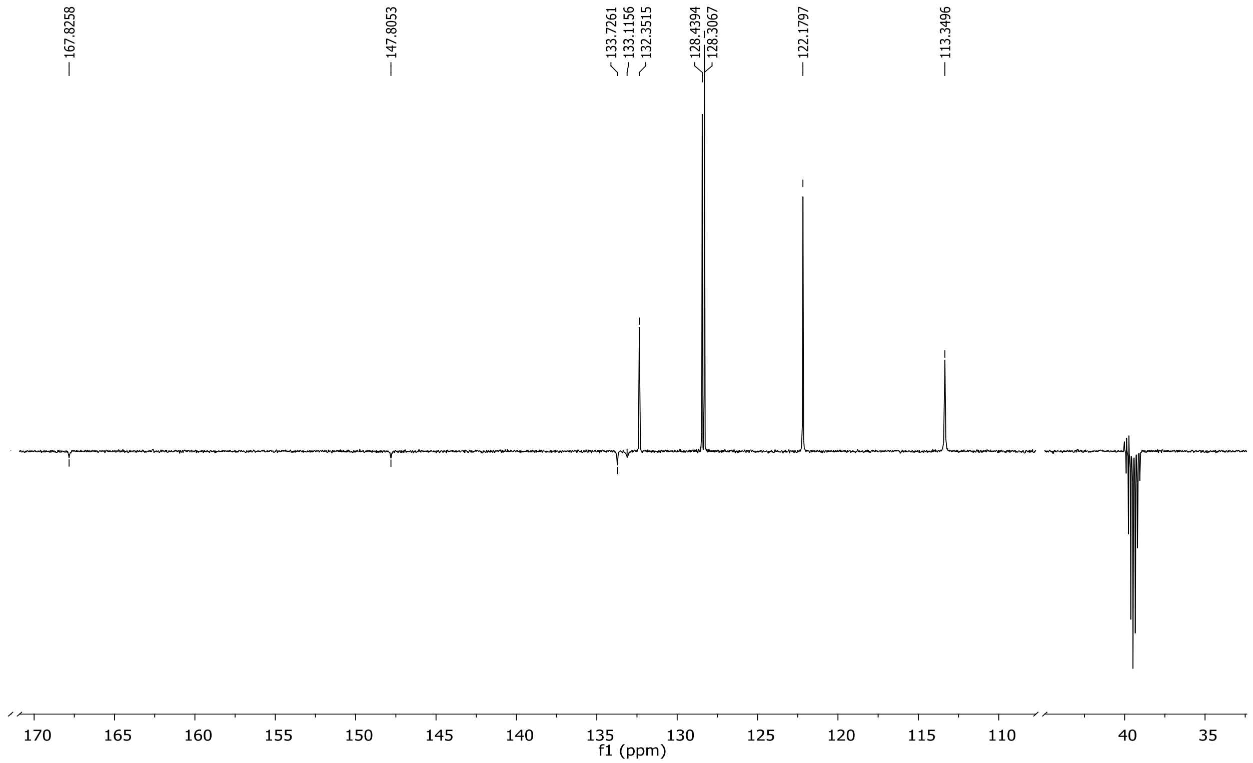

Figure S32. ^{13}C NMR spectrum (DMSO-d_6, 151 MHz) of *N-(1H-benzo[d]imidazol-2-yl)benzamide **24***

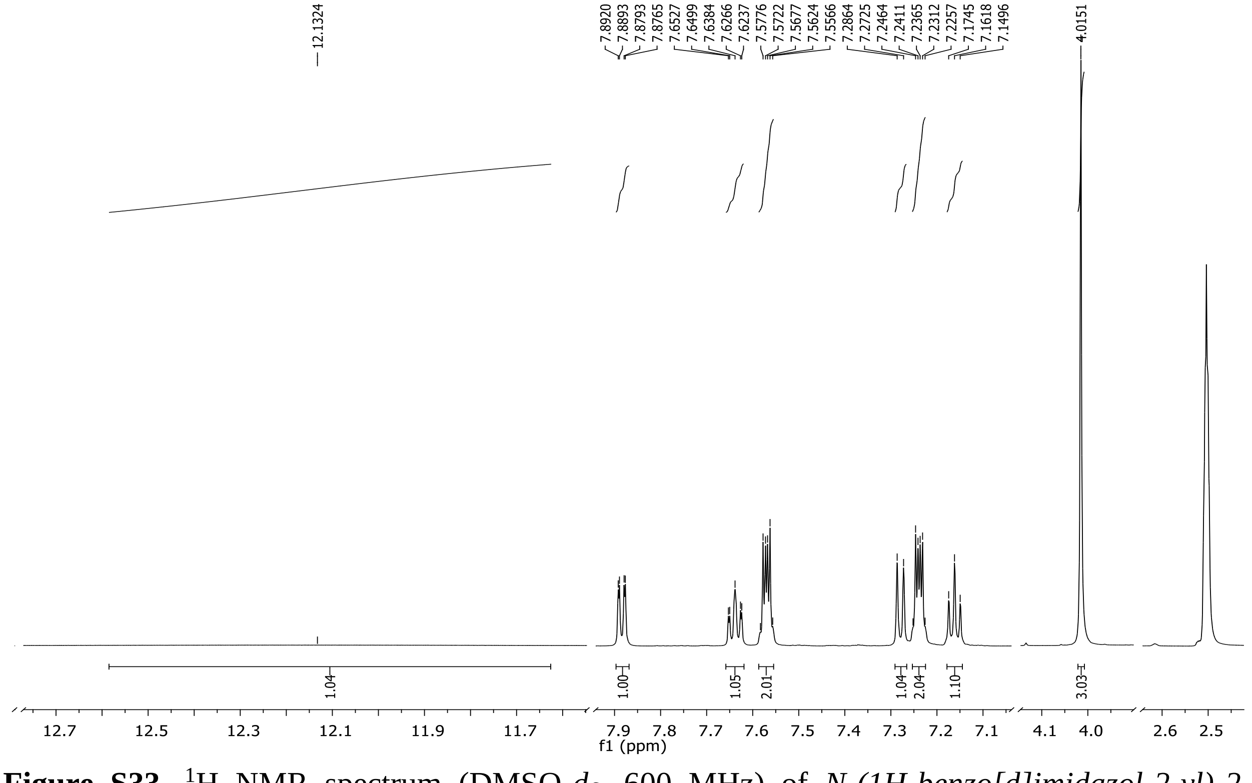

Figure S33. ^{1}H NMR spectrum (DMSO-d_6, 600 MHz) of *N-(1H-benzo[d]imidazol-2-yl)-2-methoxybenzamide **25***

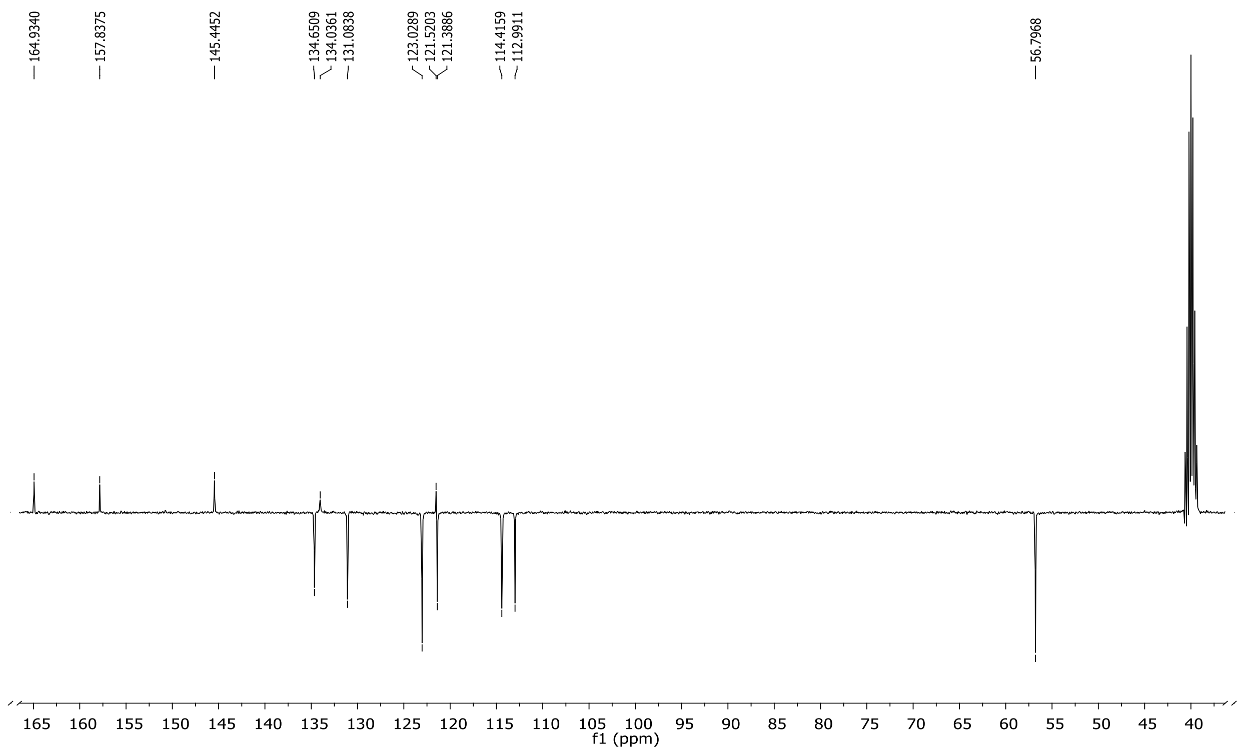

Figure S34. ^{13}C NMR spectrum (DMSO-d_6, 101 MHz) of *N-(1H-benzo[d]imidazol-2-yl)-2-methoxybenzamide **25***

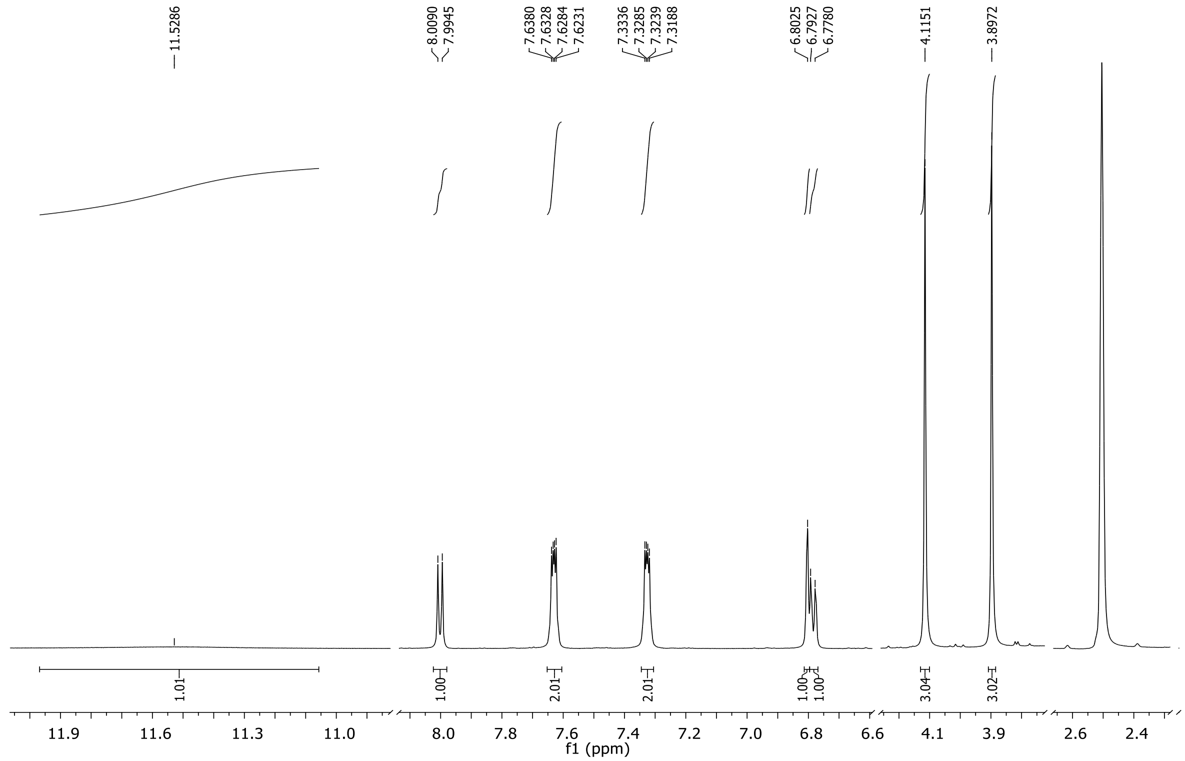

Figure S35. ^{1}H NMR spectrum (DMSO-d_6, 600 MHz) of *N-(1H-benzo[d]imidazol-2-yl)-2,4-dimethoxybenzamide **26***

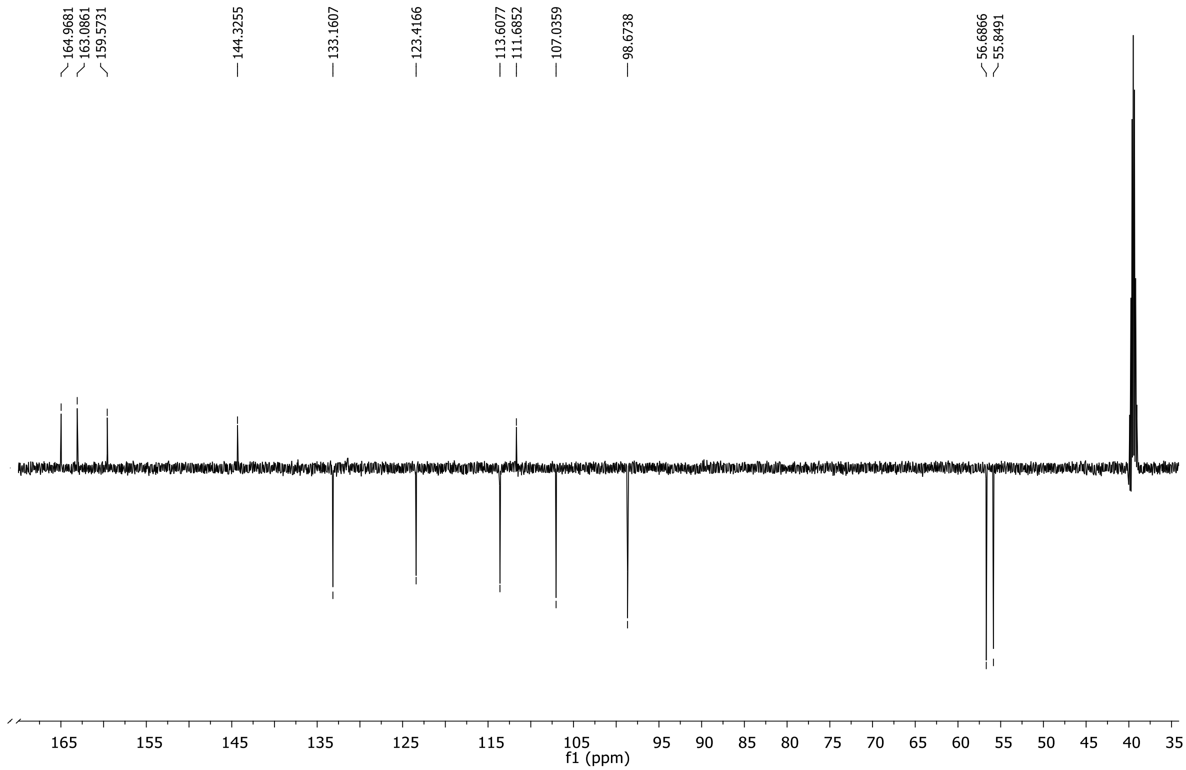

Figure S36. ^{13}C NMR spectrum (DMSO-d_6, 151 MHz) of *N-(1H-benzo[d]imidazol-2-yl)-2,4-dimethoxybenzamide* ***26***

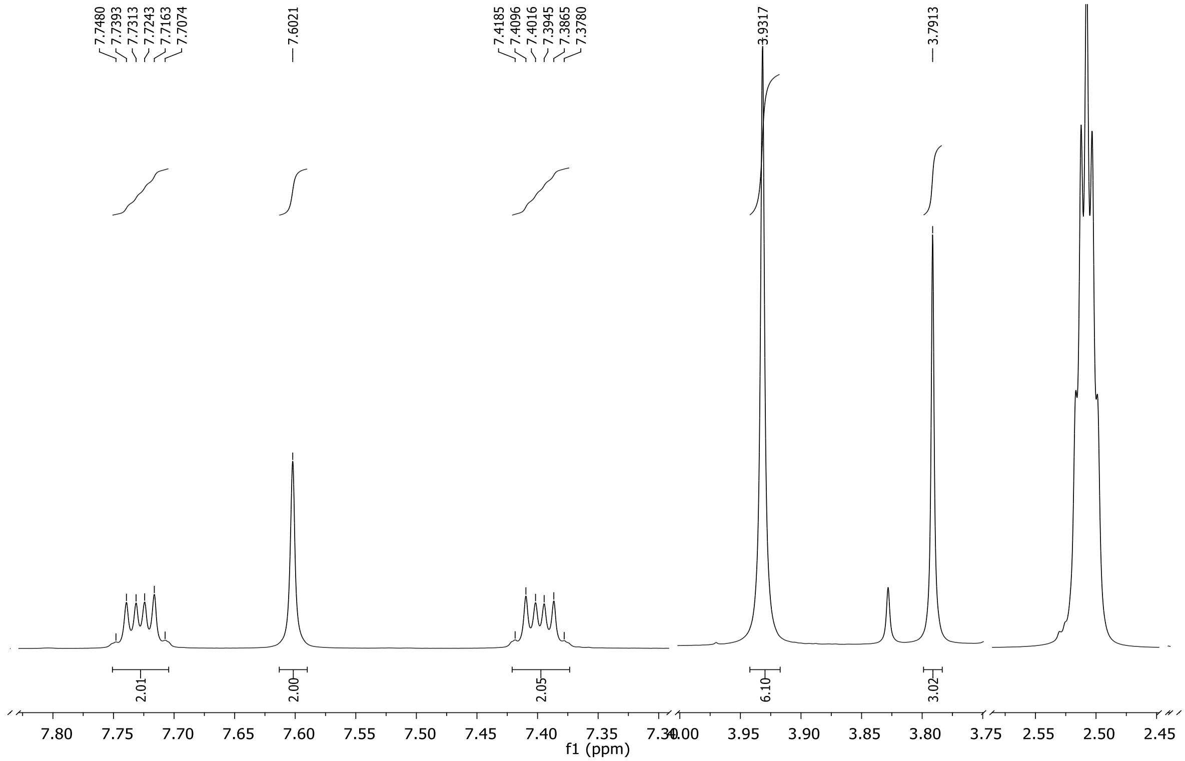

Figure S37. ^{1}H NMR spectrum (DMSO-d_6, 400 MHz) of *N-(1H-benzo[d]imidazol-2-yl)-3,4,5-trimethoxybenzamide* ***27***

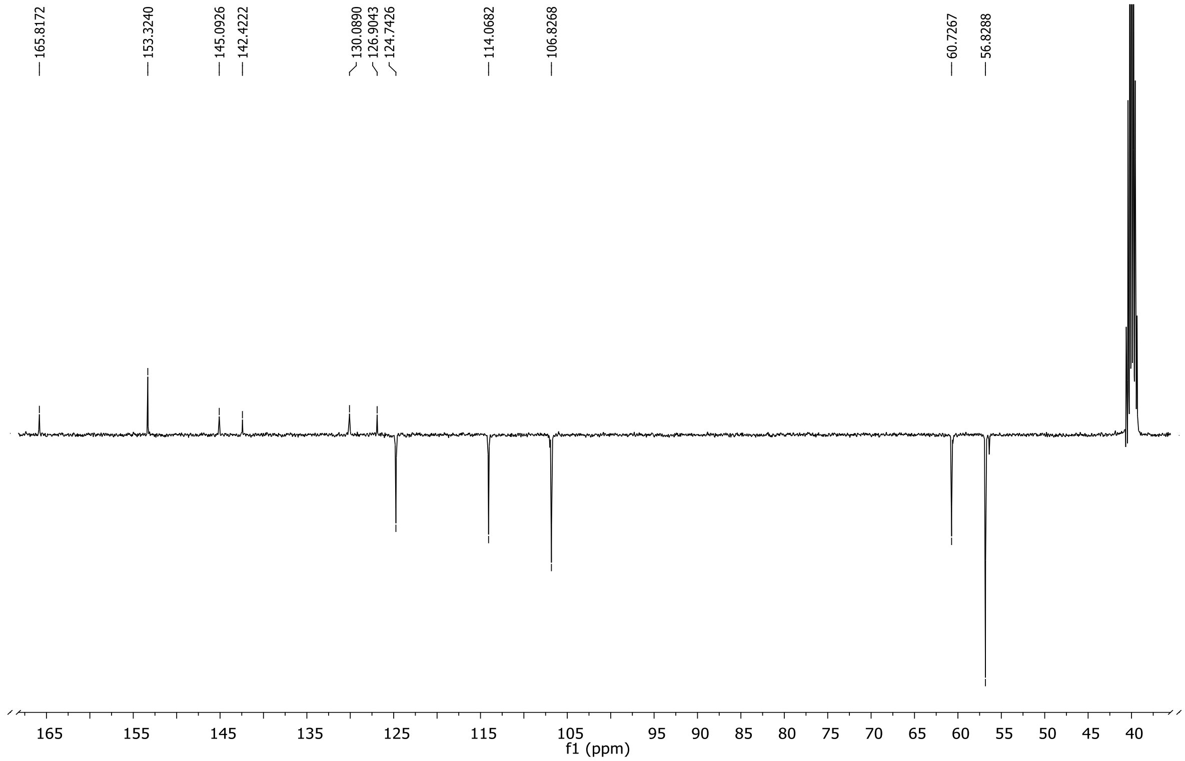

Figure S38. ^{13}C NMR spectrum (DMSO-d_6, 101 MHz) of *N-(1H-benzo[d]imidazol-2-yl)-3,4,5-trimethoxybenzamide* ***27***

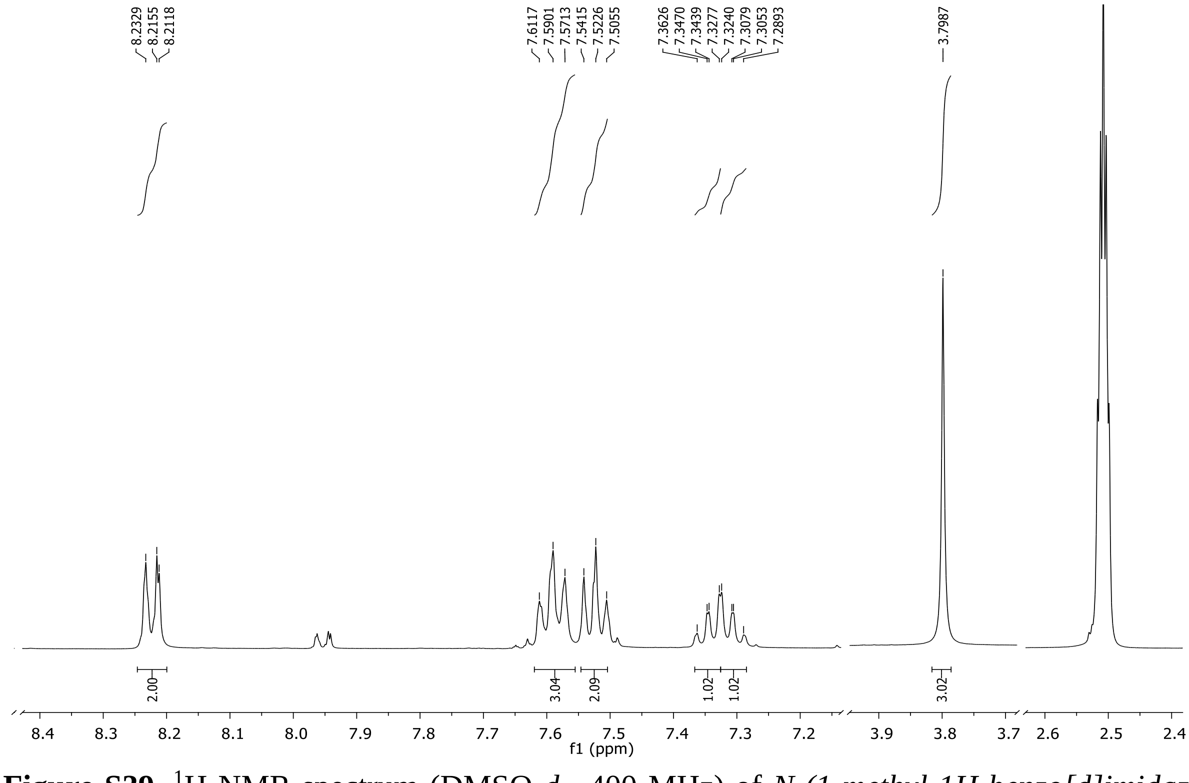

Figure S39. ^{1}H NMR spectrum (DMSO-d_6, 400 MHz) of *N-(1-methyl-1H-benzo[d]imidazol-2-yl)benzamide* ***28***

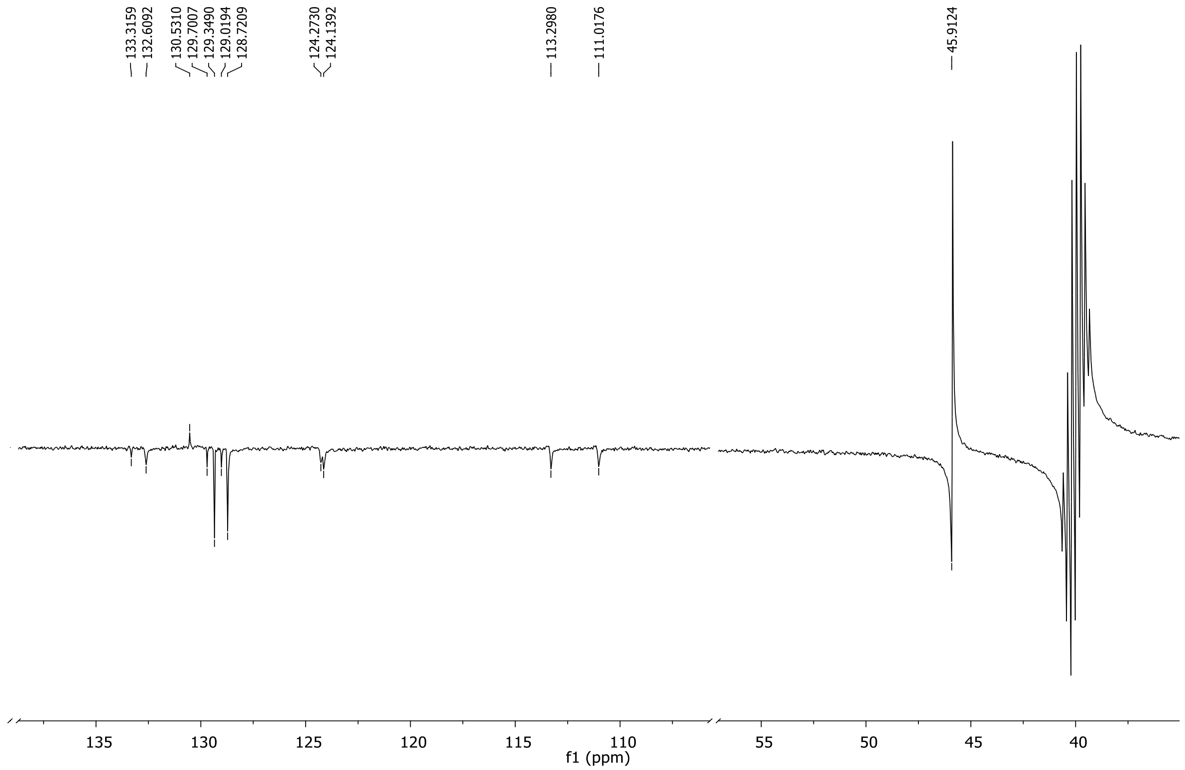

Figure S40. ^{13}C NMR spectrum (DMSO-d_6, 101 MHz) of *N-(1-methyl-1H-benzo[d]imidazol-2-yl)benzamide* ***28***

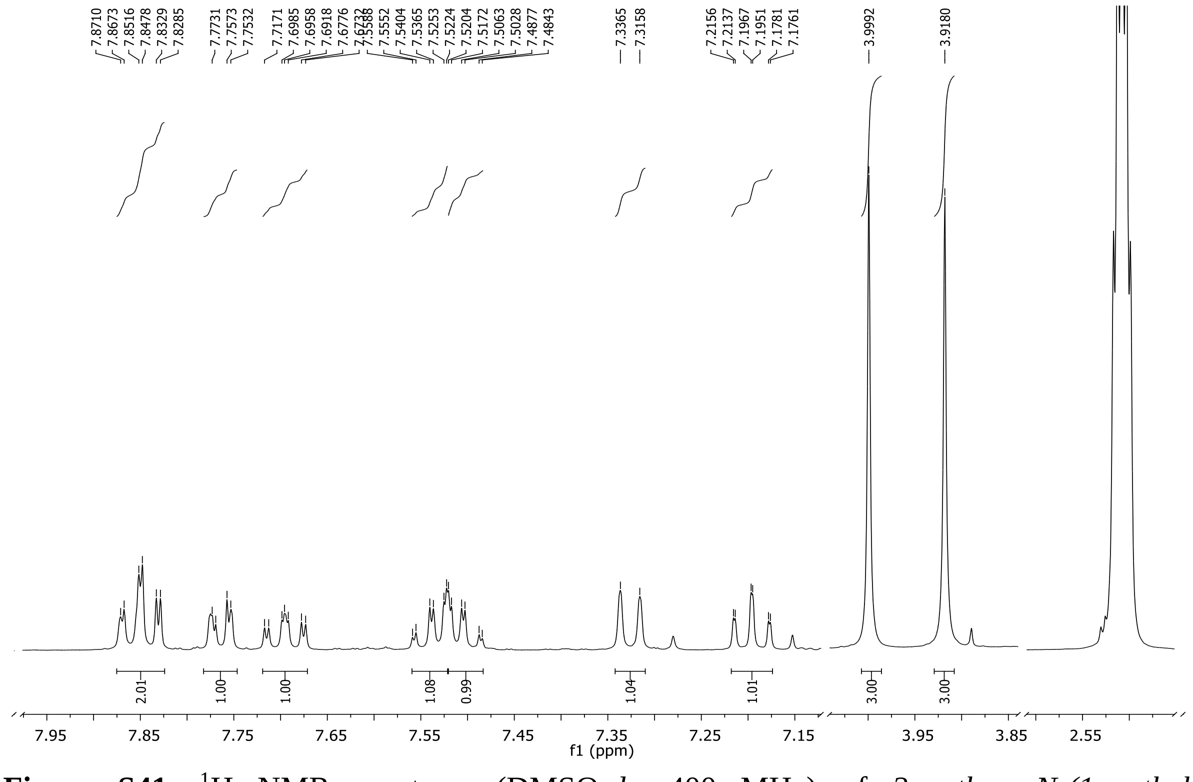

Figure S41. ^{1}H NMR spectrum (DMSO-d_6, 400 MHz) of *2-methoxy-N-(1-methyl-1H-benzo[d]imidazol-2-yl)benzamide* ***29***

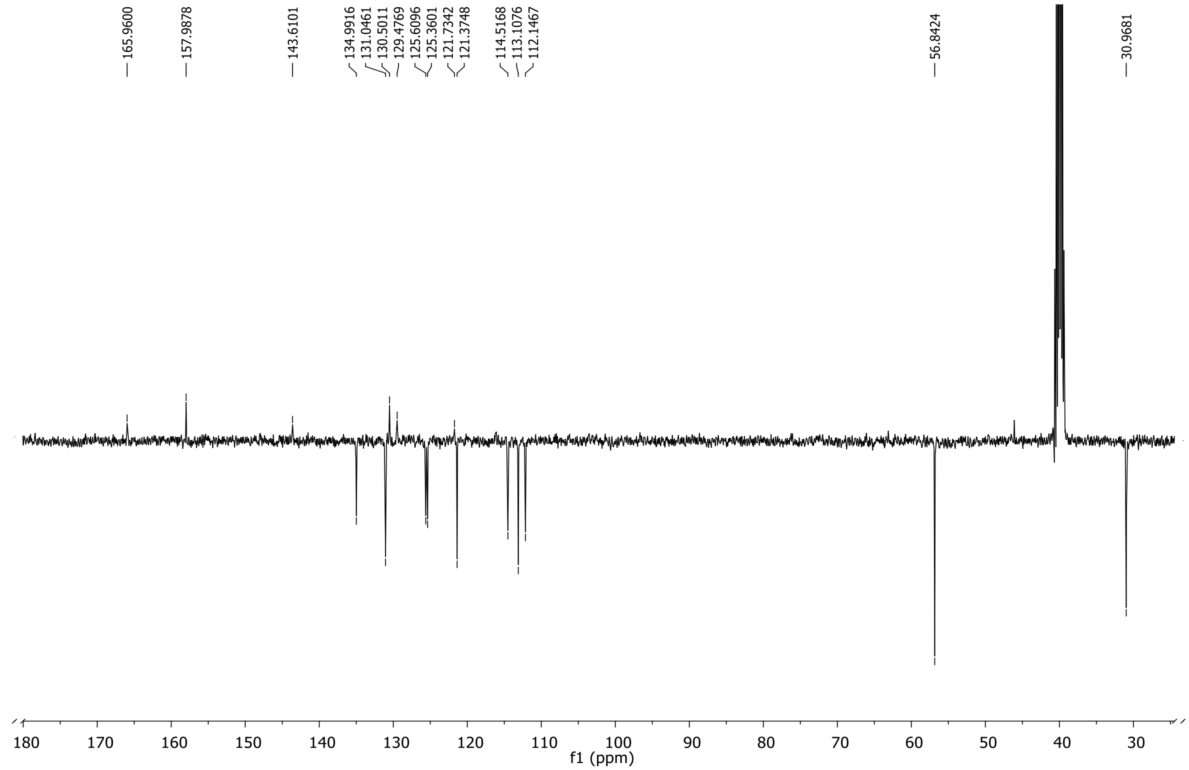

Figure S42. ^{13}C NMR spectrum (DMSO-d_6, 101 MHz) of *2-methoxy-N-(1-methyl-1H-benzo[d]imidazol-2-yl)benzamide* ***29***

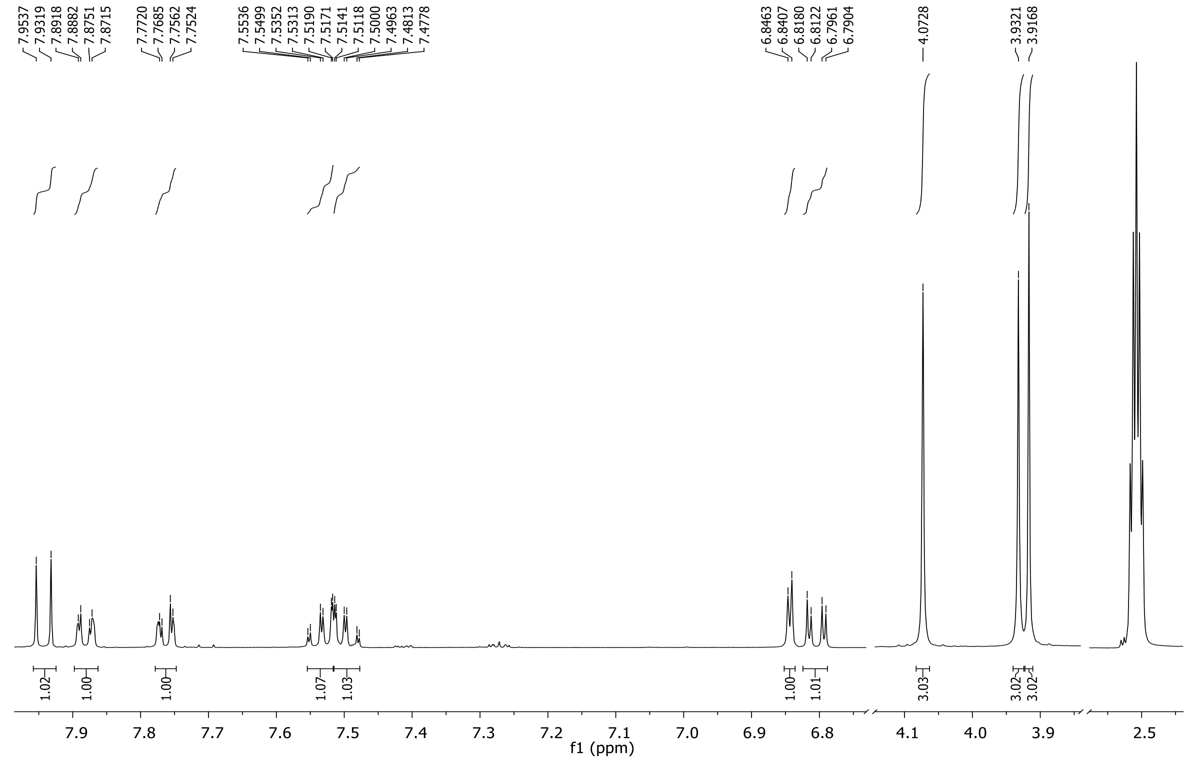

Figure S43. ^{1}H NMR spectrum (DMSO-d_6, 400 MHz) of *2,4-dimethoxy-N-(1-methyl-1H-benzo[d]imidazol-2-il)benzamide* ***30***

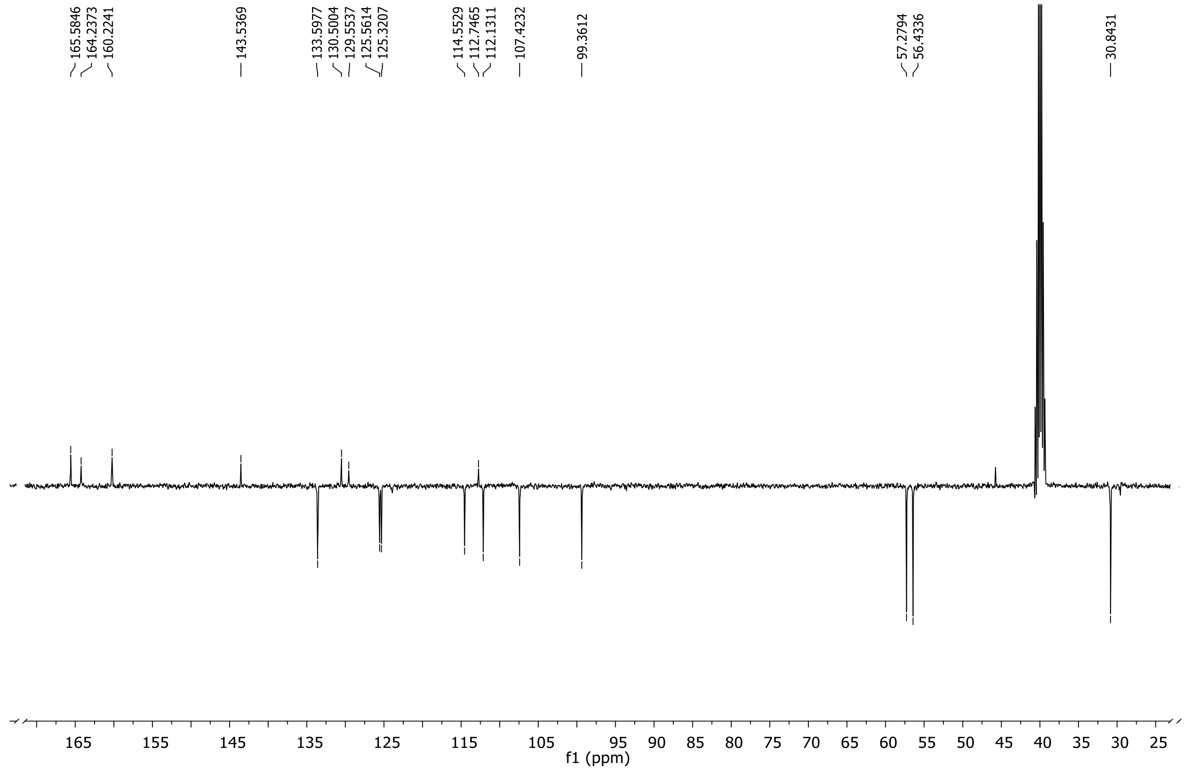

Figure S44. ^{13}C NMR spectrum (DMSO-d_6, 101 MHz) of *2,4-dimethoxy-N-(1-methyl-1H-benzo[d]imidazol-2-il)benzamide **30***

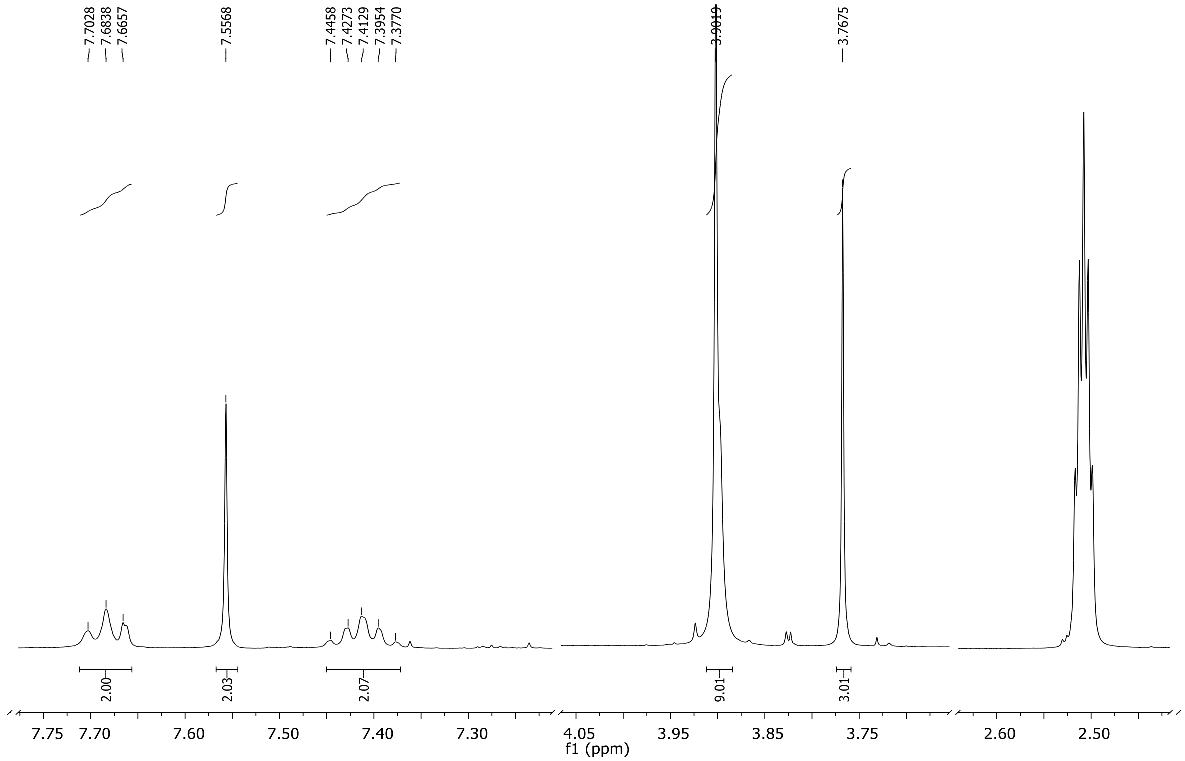

Figure S45. ^{1}H NMR spectrum (DMSO-d_6, 400 MHz) of *3,4,5-trimethoxy-N-(1-methyl-1H-benzo[d]imidazol-2-yl)benzamide **31***

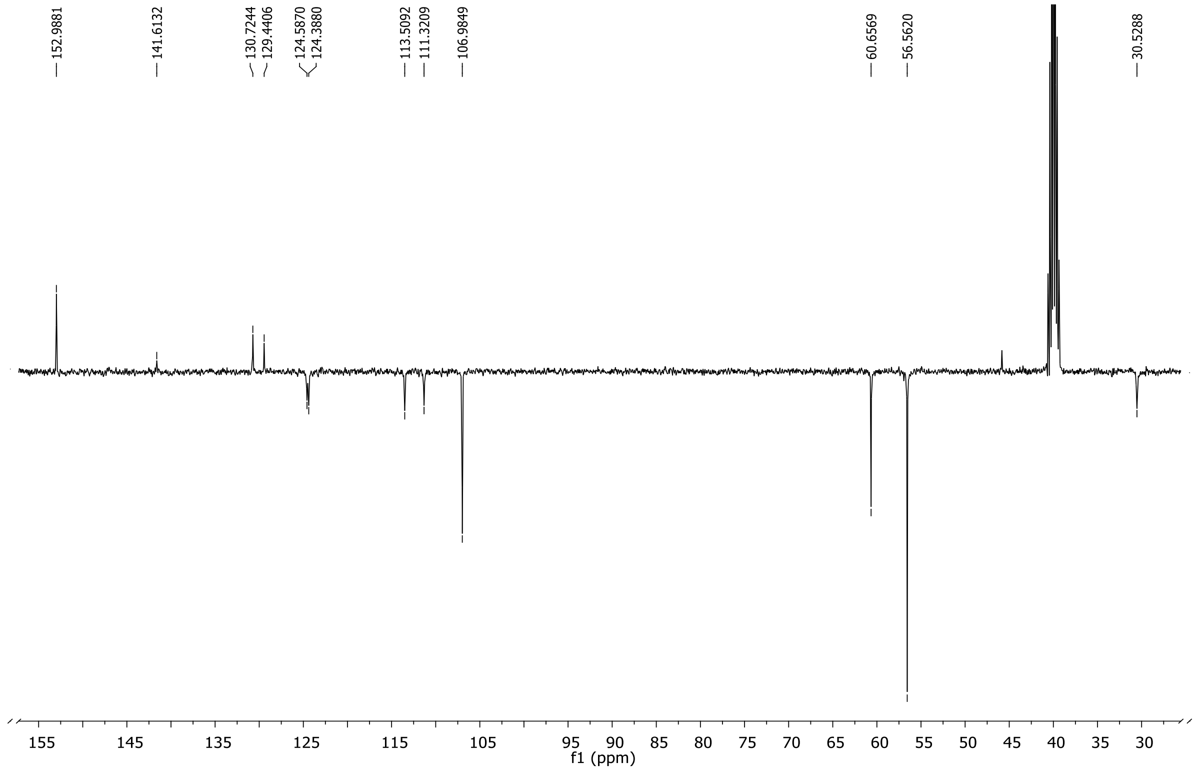

Figure S46. ^{13}C NMR spectrum (DMSO-d_6, 101 MHz) of *3,4,5-trimethoxy-N-(1-methyl-1H-benzo[d]imidazol-2-yl)benzamide* ***31***

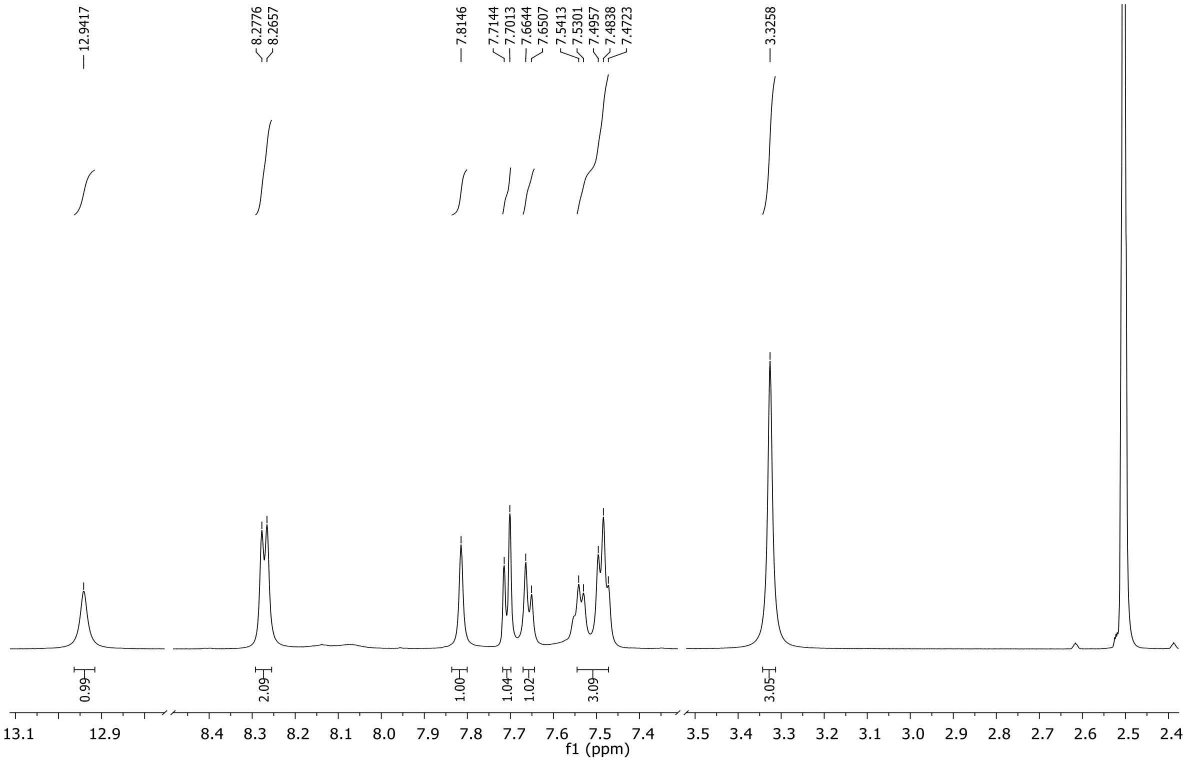

Figure S47. ^{1}H NMR spectrum (DMSO-d_6, 600 MHz) of *N-(6-cyano-1-methyl-1H-benzo[d]imidazol-2-yl)benzamide* ***32***

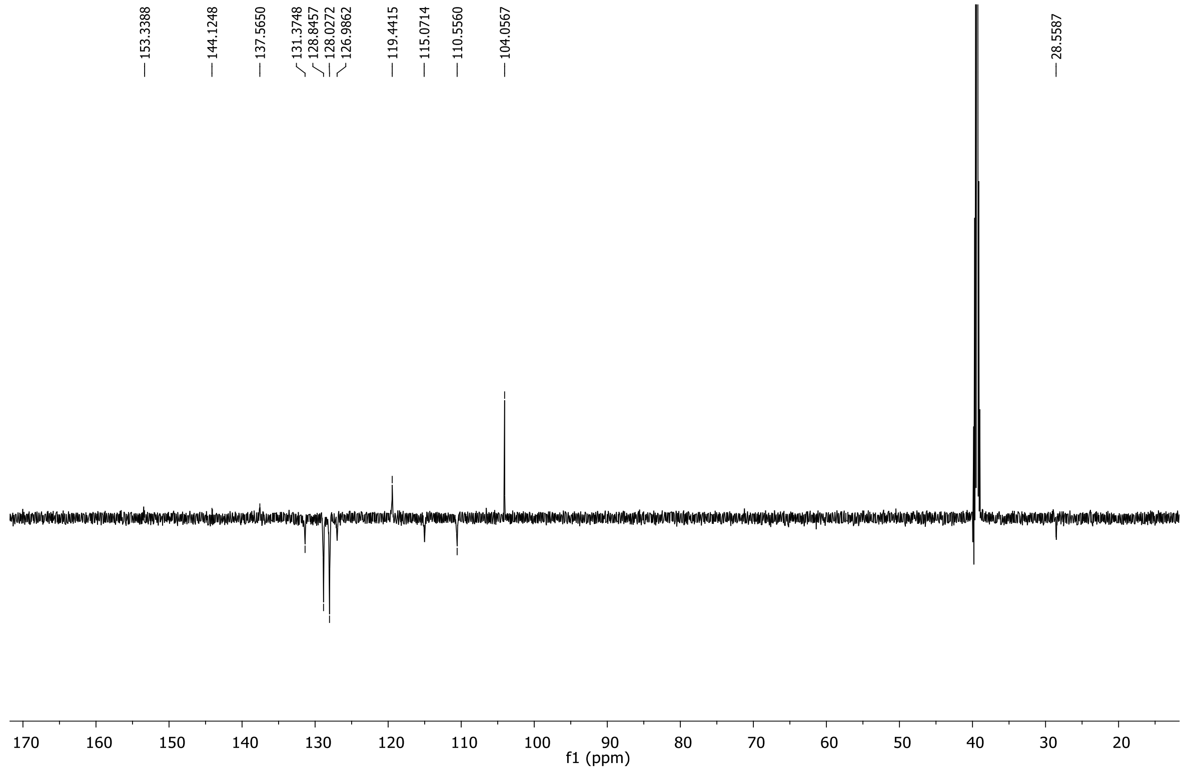

Figure S48. ^{13}C NMR spectrum (DMSO-d_6, 151 MHz) of *N-(6-cyano-1-methyl-1H-benzo[d]imidazol-2-yl)benzamid**e** **32***

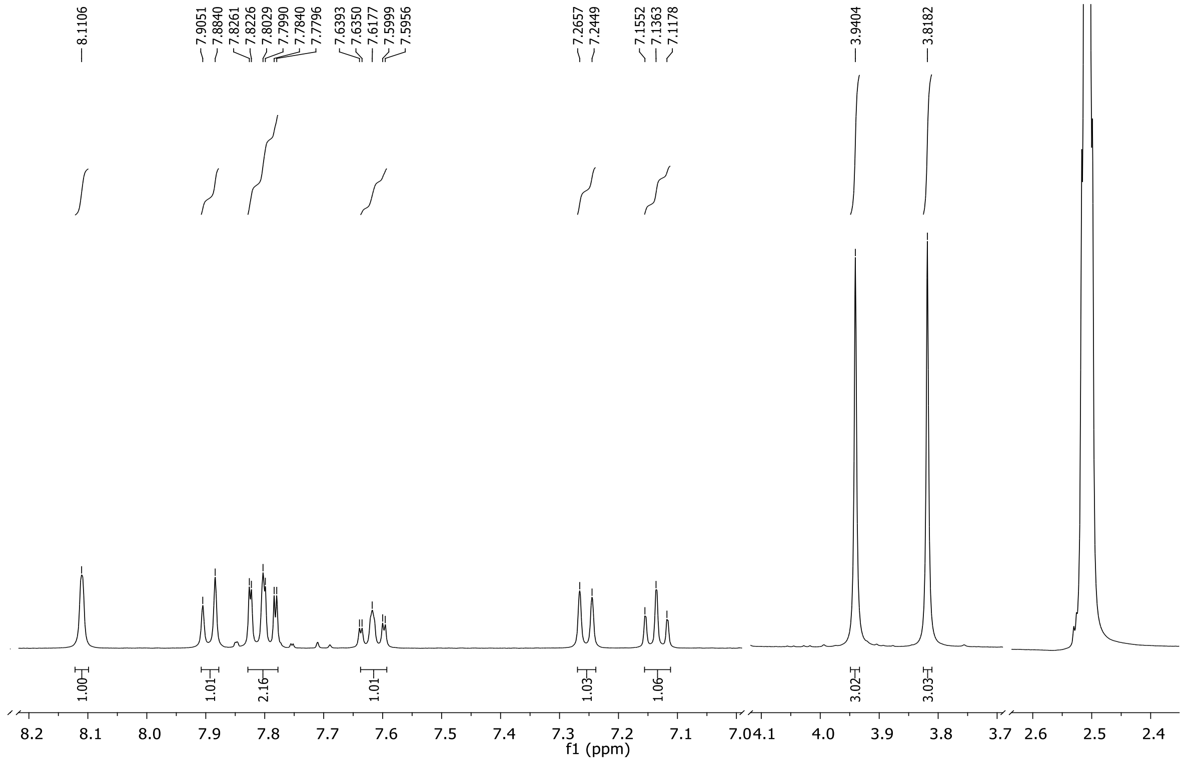

Figure S49. ^{1}H NMR spectrum (DMSO-d_6, 400 MHz) of *N-(6-cyano-1-methyl-1H-benzo[d]imidazol-2-yl)-2-methoxybenzamide **33***

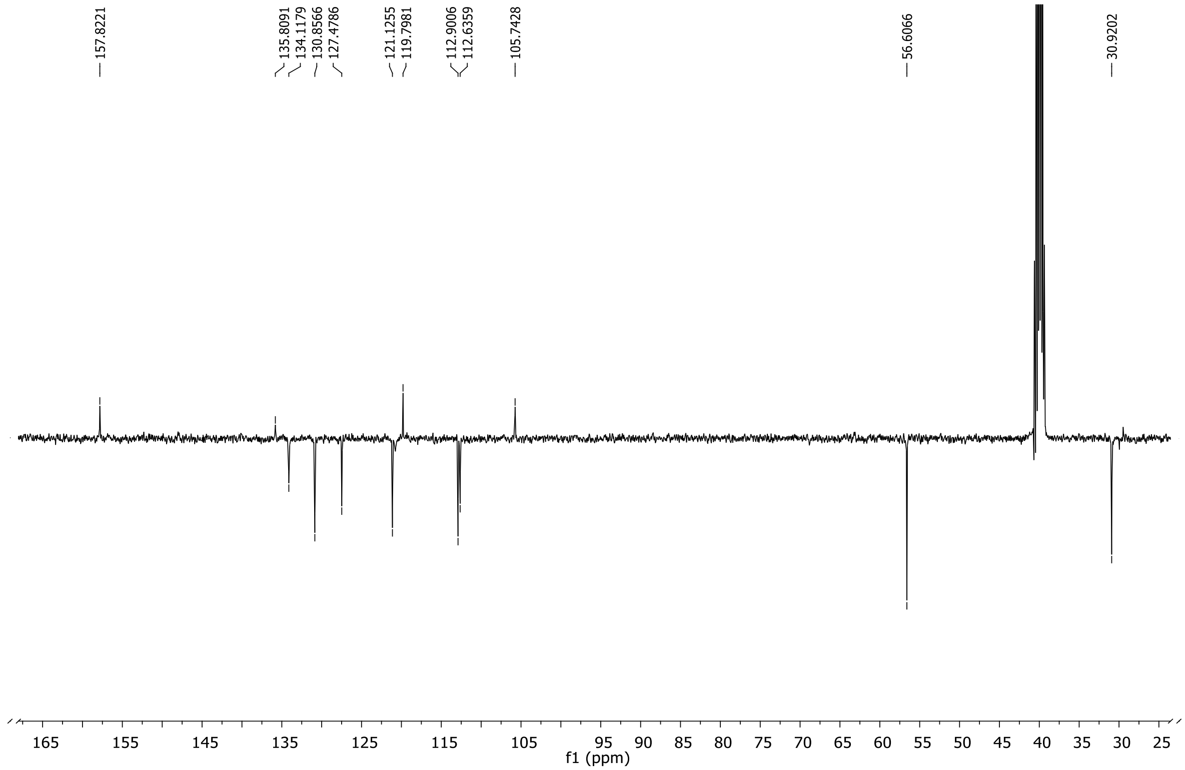

Figure S50. ^{13}C NMR spectrum (DMSO-d_6, 101 MHz) of *N-(6-cyano-1-methyl-1H-benzo[d]imidazol-2-yl)-2-methoxybenzamide **33***

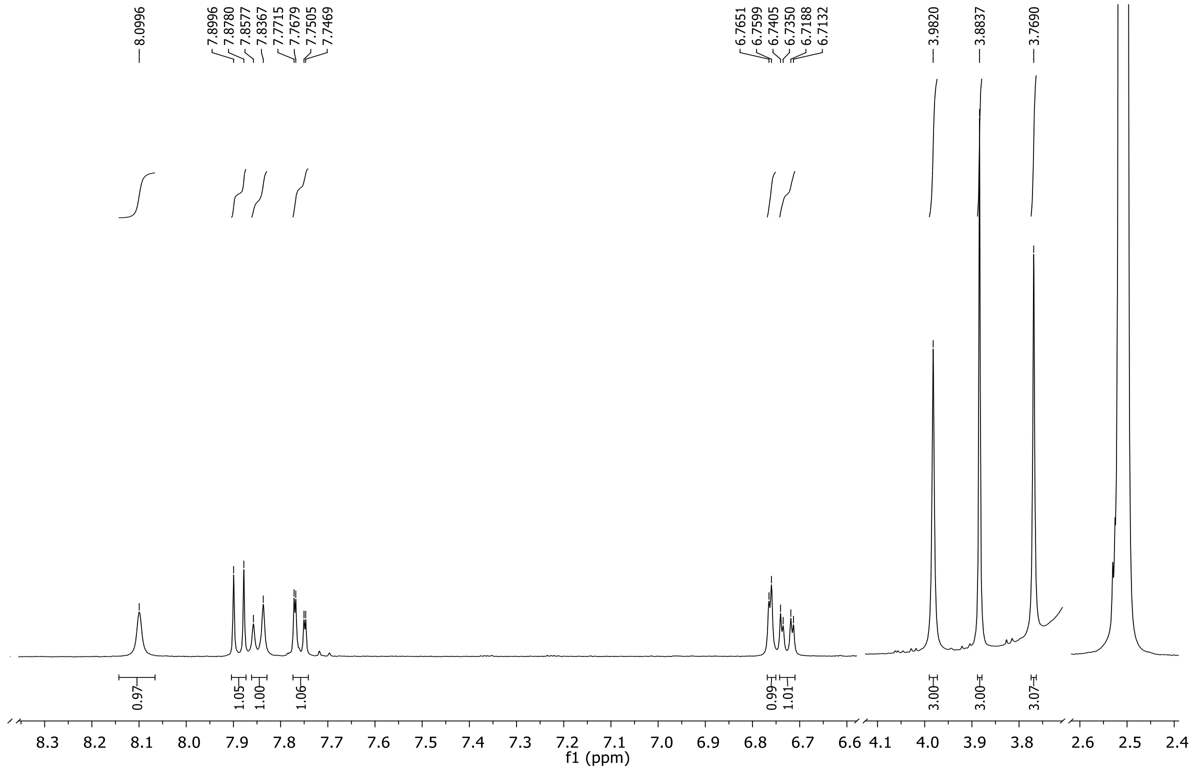

Figure S51. ^{1}H NMR spectrum (DMSO-d_6, 400 MHz) of *N-(6-cyano-1-methyl-1H-benzo[d]imidazol-2-yl)-2,4-dimethoxybenzamide **34***

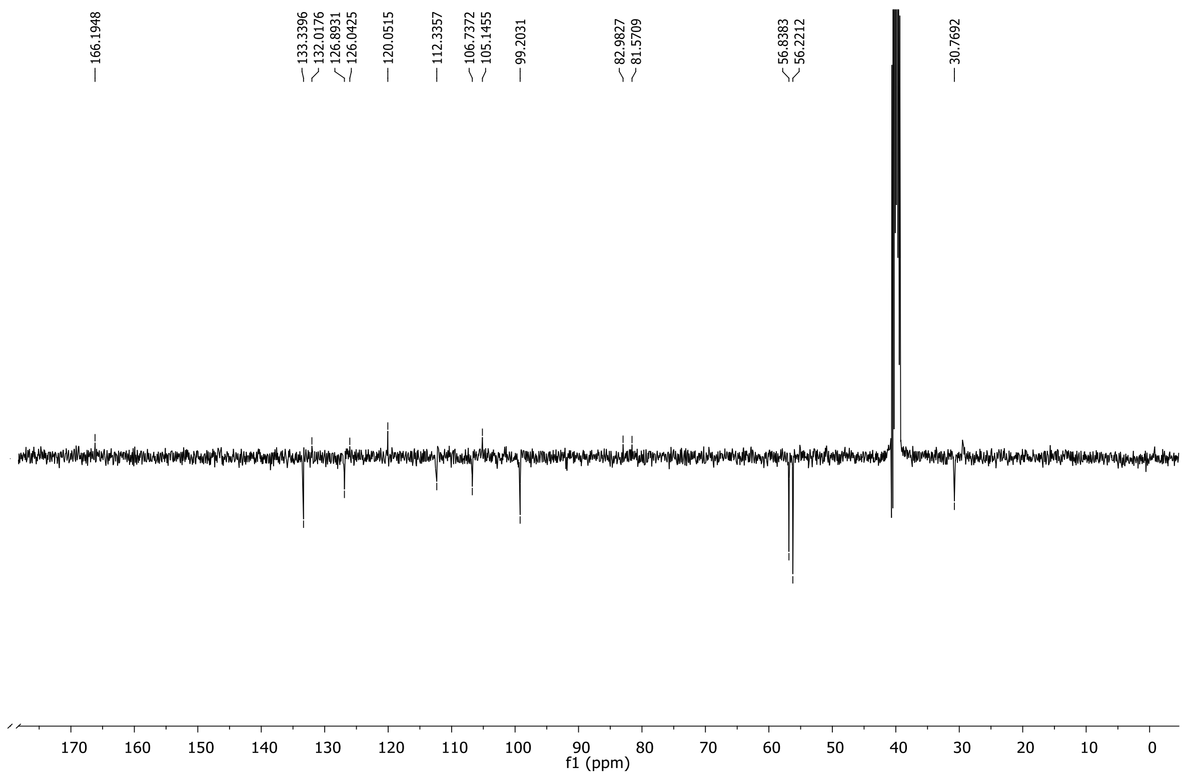

Figure S52. ^{13}C NMR spectrum (DMSO-d_6, 101 MHz) of *N-(6-cyano-1-methyl-1H-benzo[d]imidazol-2-yl)-2,4-dimethoxybenzamide* ***34***

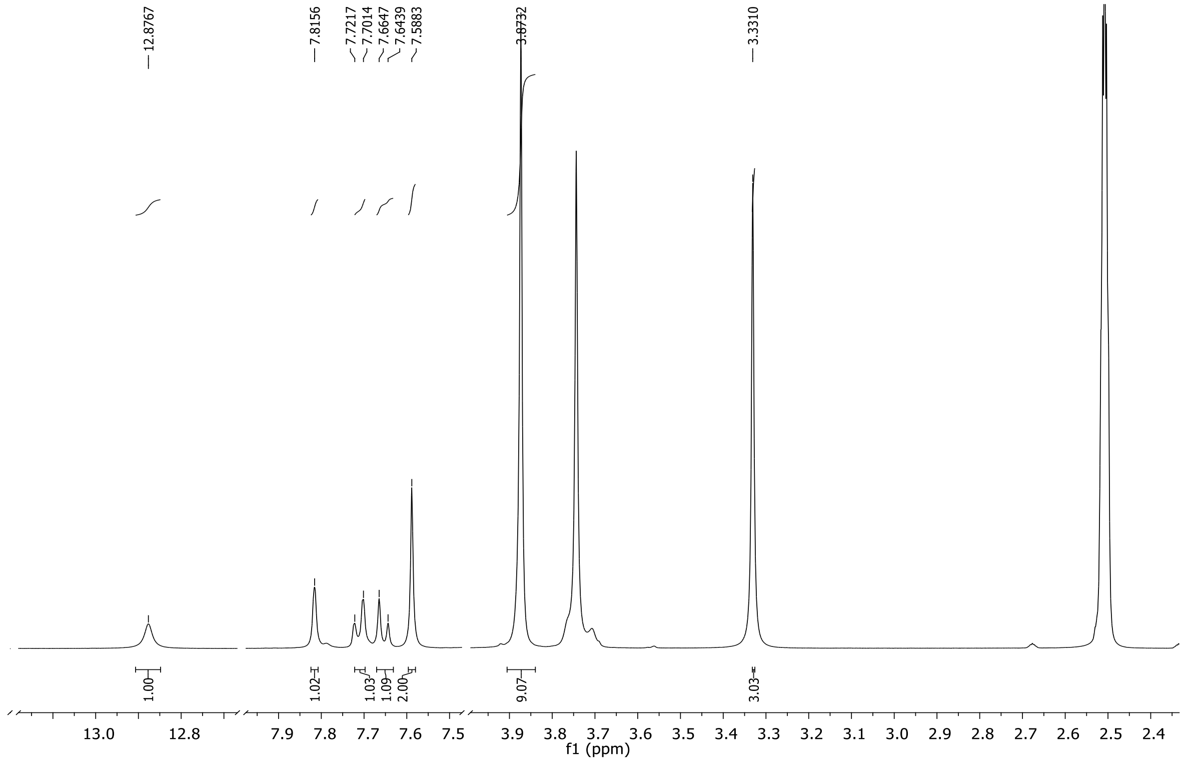

Figure S53. ^{1}H NMR spectrum (DMSO-d_6, 400 MHz) of *N-(6-cyano-1-methyl-1H-benzo[d]imidazol-2-yl)-3,4,5-trimethoxybenzamide* ***35***

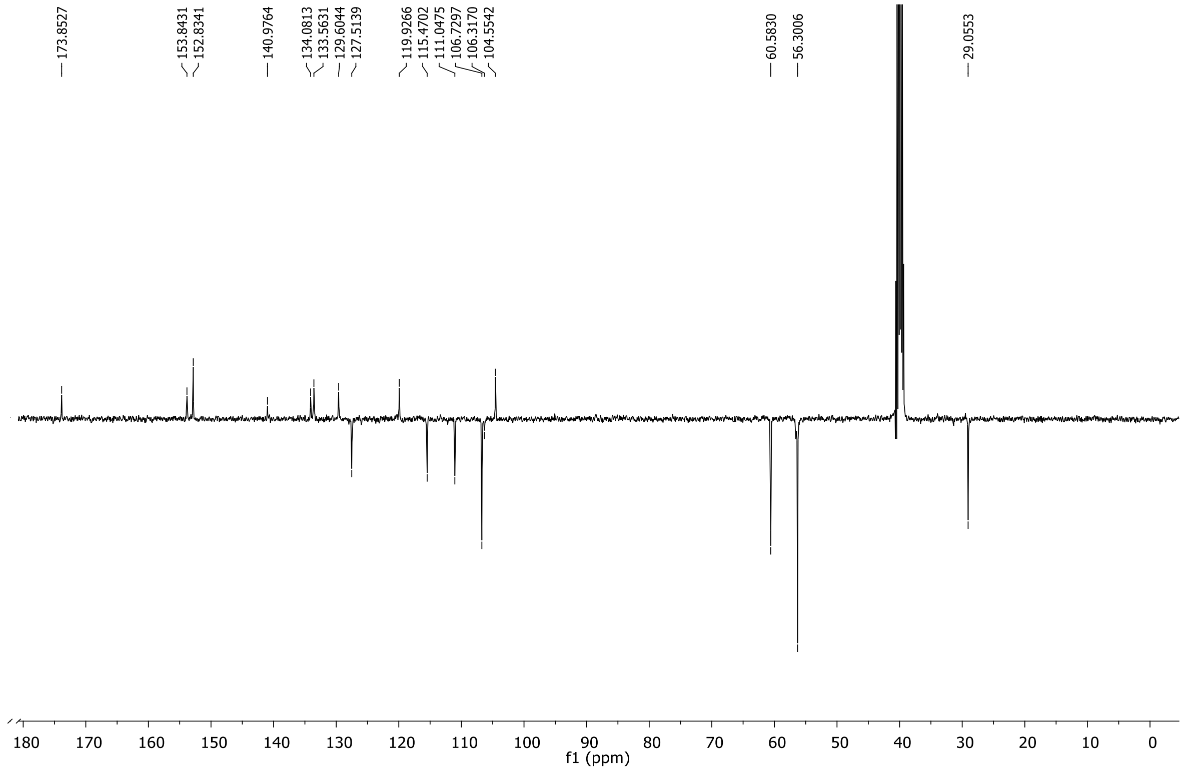

Figure S54. ^{13}C NMR spectrum (DMSO-d_6, 101 MHz) of *N-(6-cyano-1-methyl-1H-benzo[d]imidazol-2-yl)-3,4,5-trimethoxybenzamide* ***35***

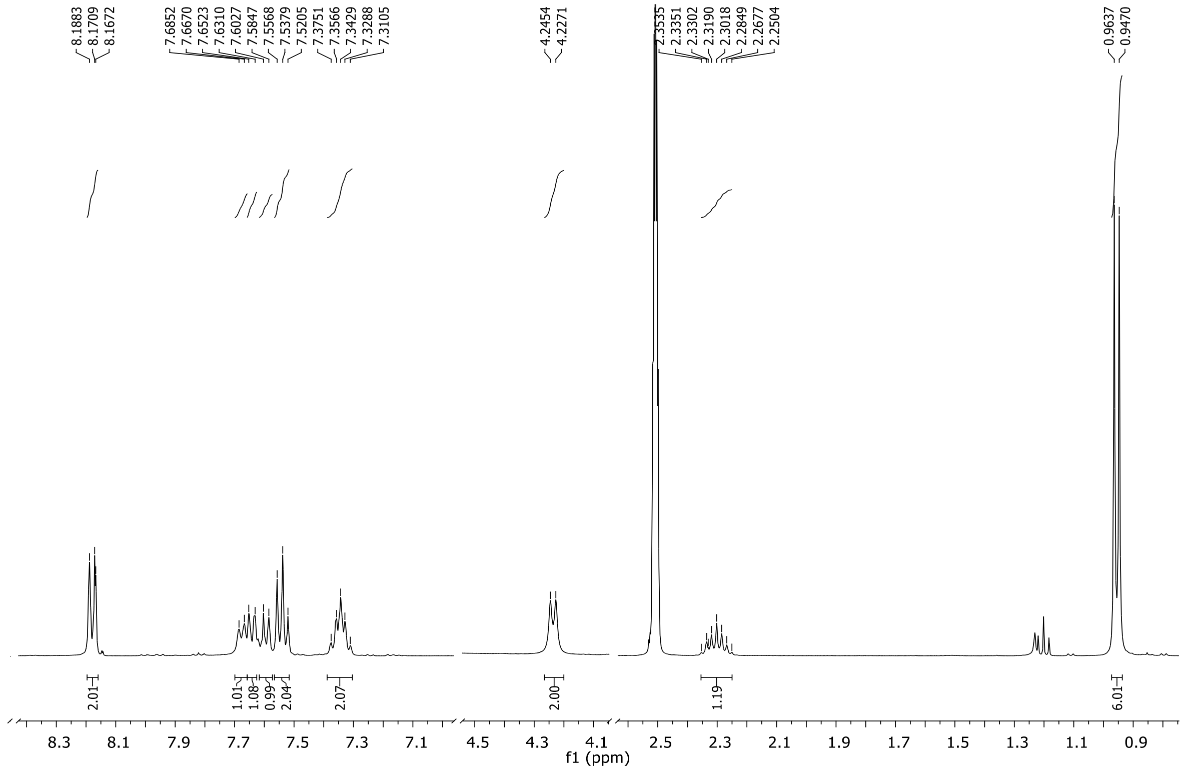

Figure S55. ^{1}H NMR spectrum (DMSO-d_6, 400 MHz) of *N-(1-isobutyl-1H-benzo[d]imidazol-2-yl)benzamide* ***36***

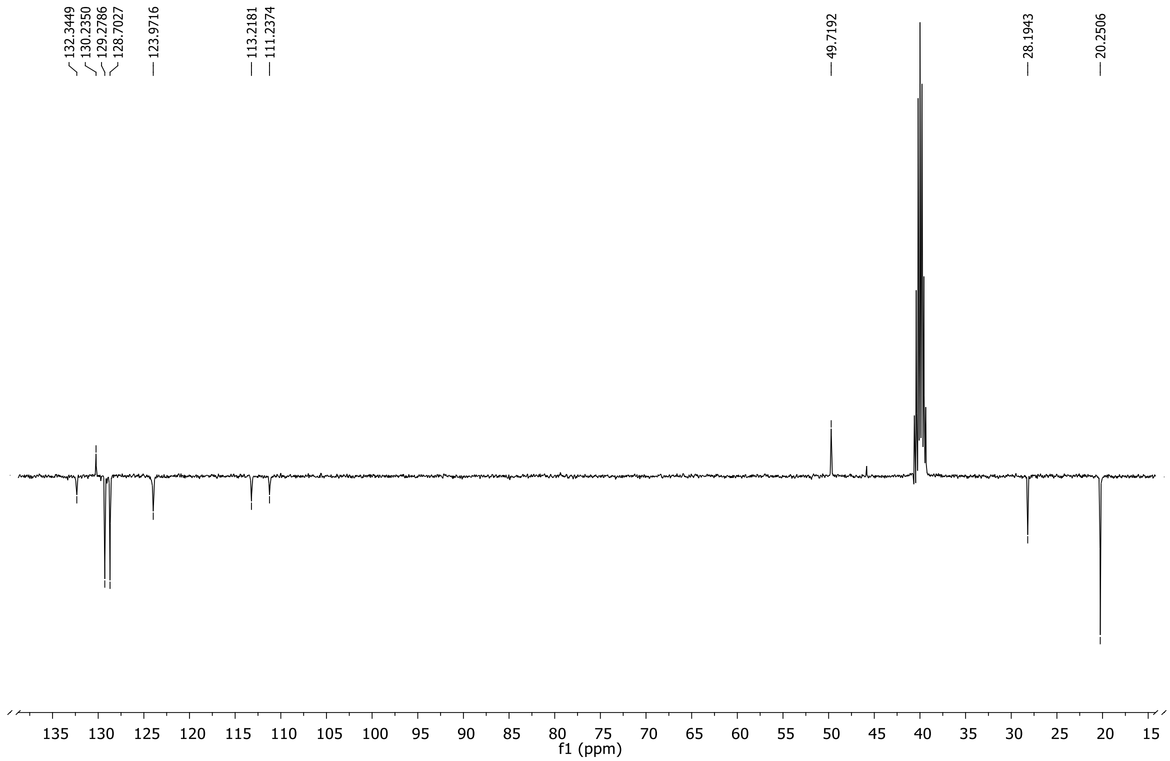

Figure S56. ^{13}C NMR spectrum (DMSO-d_6, 101 MHz) of *N-(1-isobutyl-1H-benzo[d]imidazol-2-yl)benzamide* ***36***

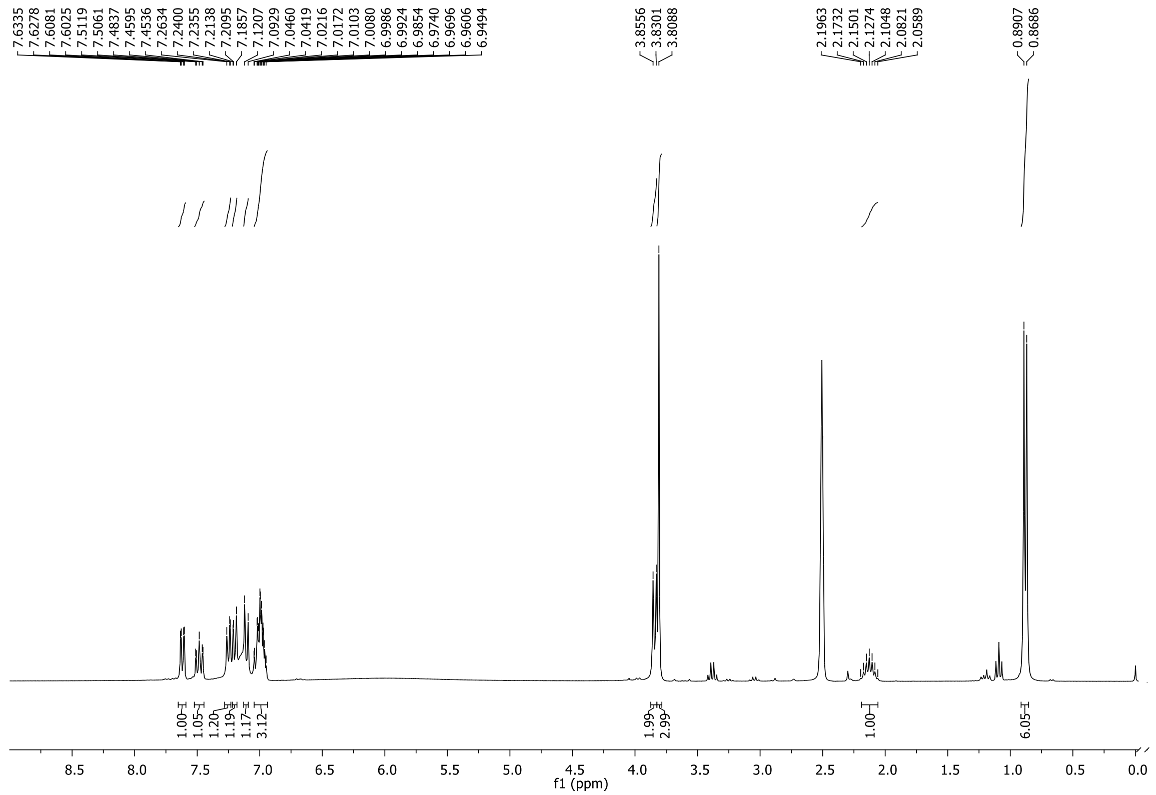

Figure S57. ^{1}H NMR spectrum (DMSO-d_6, 300 MHz) of *N-(1-isobutyl-1H-benzo[d]imidazol-2-yl)-2-methoxybenzamide* ***37***

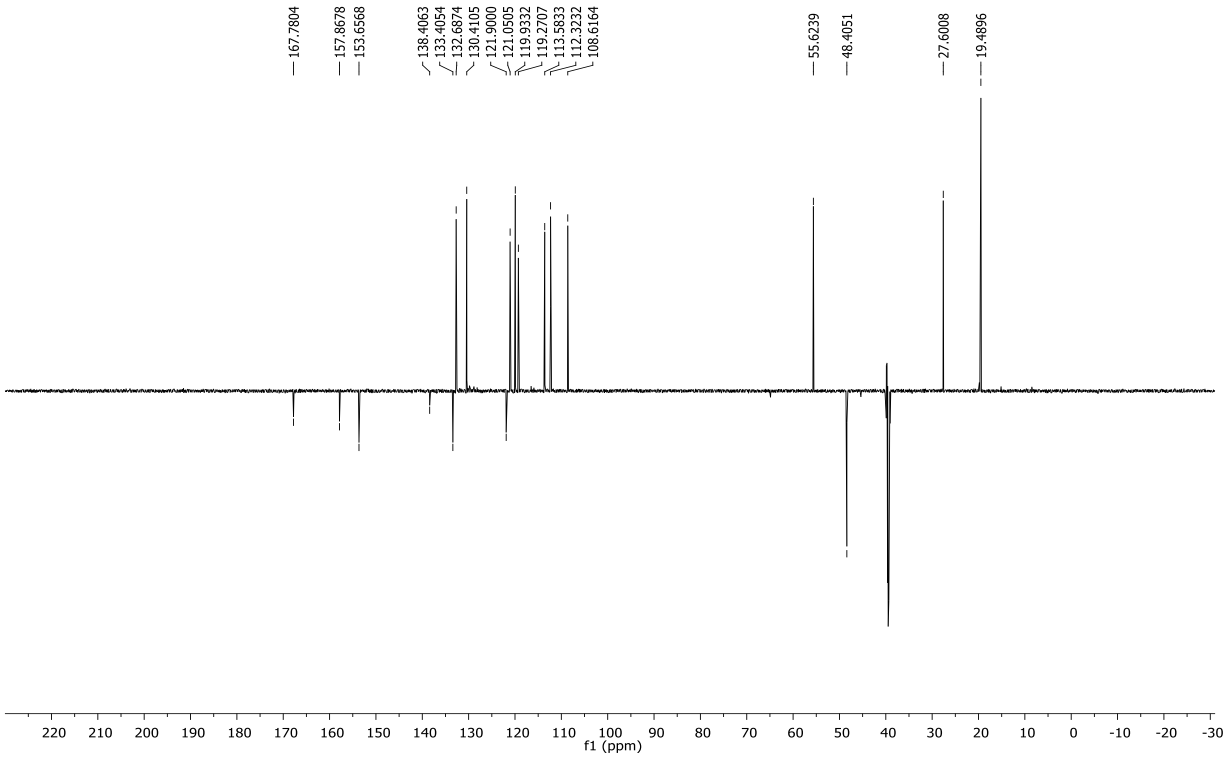

Figure S58. ^{13}C NMR spectrum (DMSO-d_6, 151 MHz) of *N-(1-isobutyl-1H-benzo[d]imidazol-2-yl)-2-methoxybenzamide* ***37***

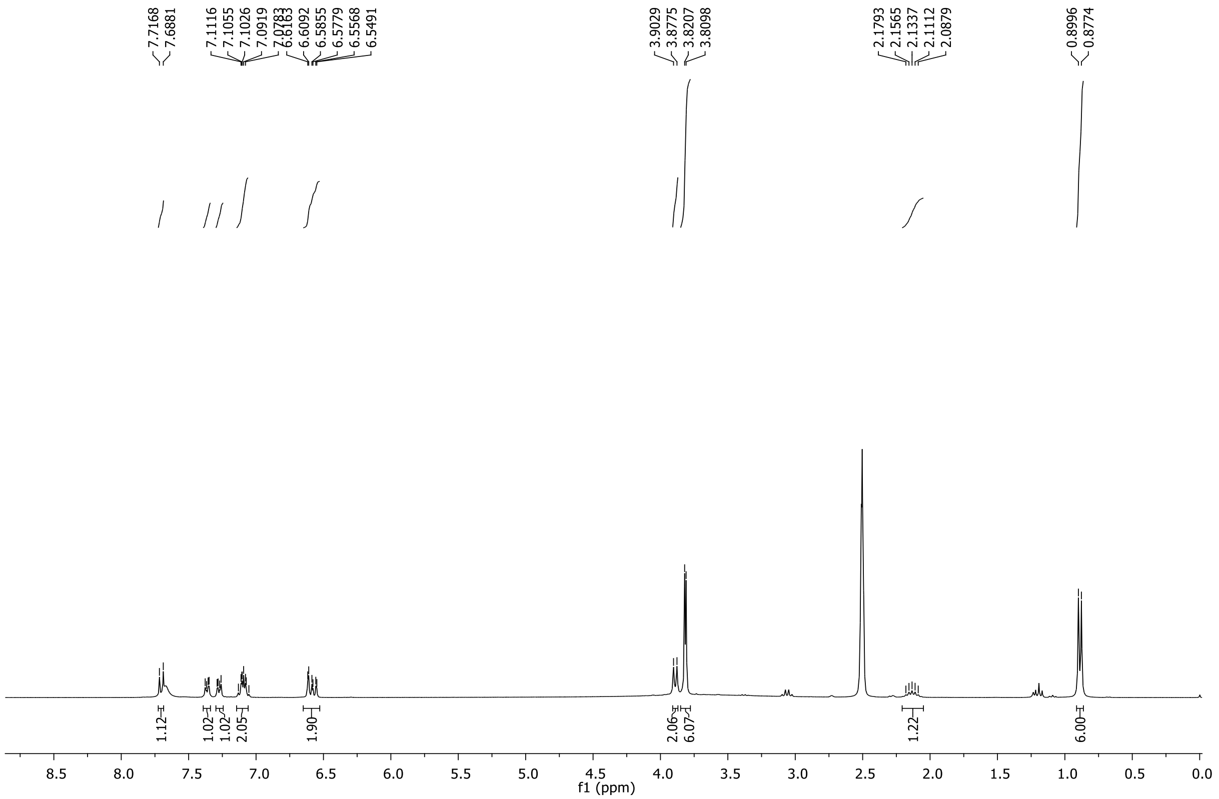

Figure S59. ^{1}H NMR spectrum (DMSO-d_6, 300 MHz) of *N-(1-isobutyl-1H-benzo[d]imidazol-2-yl)-2,4-dimethoxybenzamide* ***38***

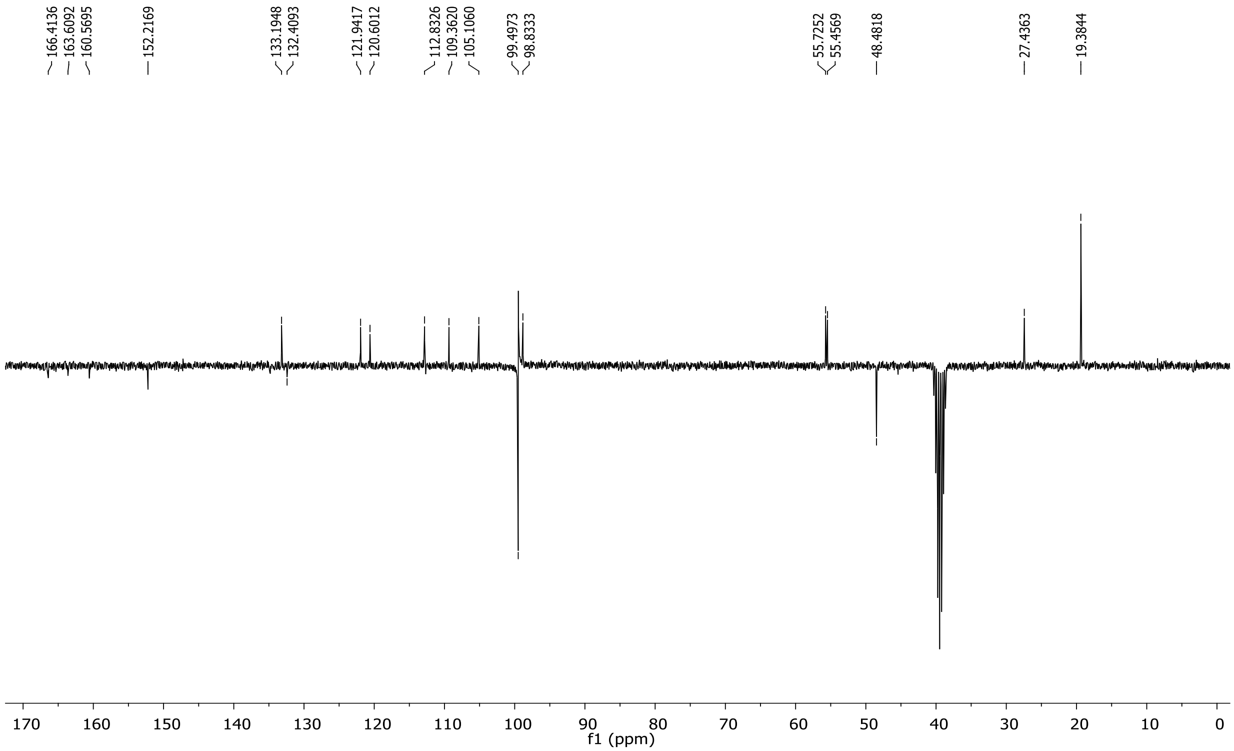

Figure S60. ^{13}C NMR spectrum (DMSO-d_6, 75 MHz) of *N-(1-isobutyl-1H-benzo[d]imidazol-2-yl)-2,4-dimethoxybenzamide* ***38***

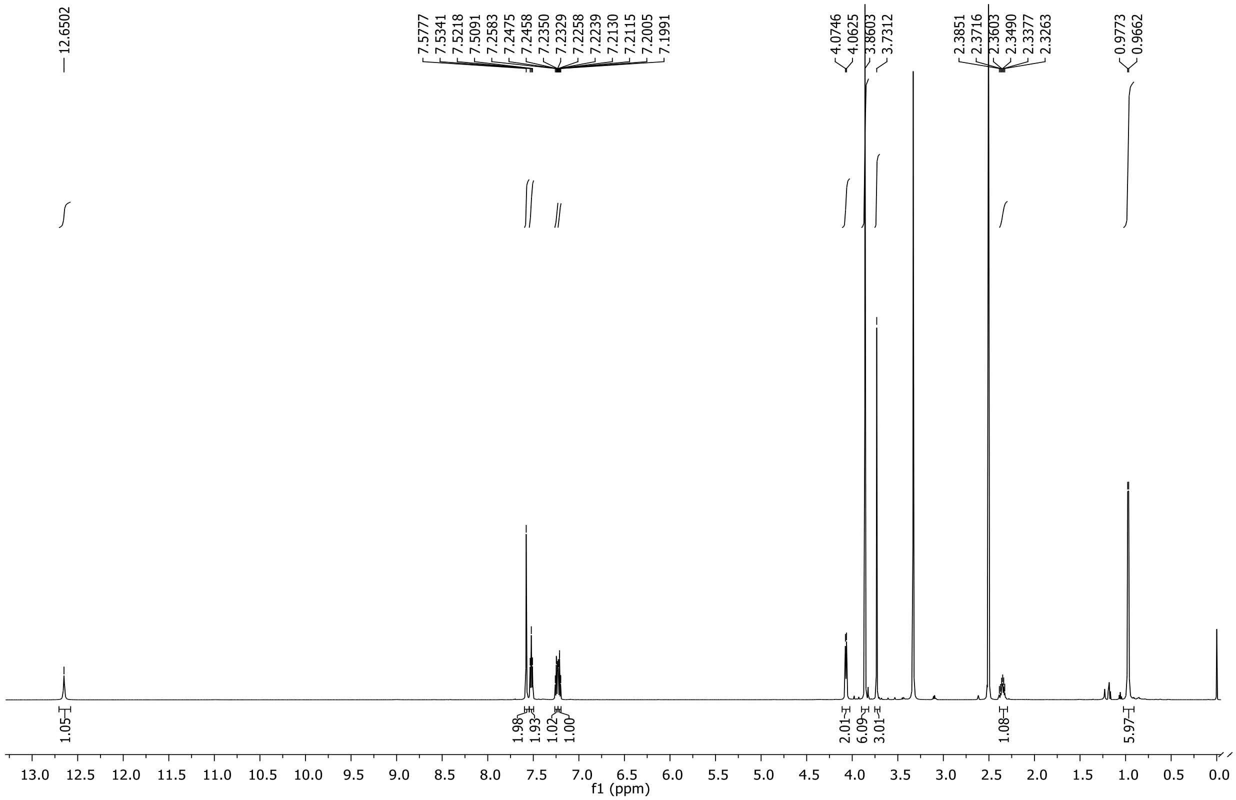

Figure S61. ^{1}H NMR spectrum (DMSO-d_6, 600 MHz) of *N-(1-isobutyl-1H-benzo[d]imidazol-2-yl)-3,4,5-trimethoxybenzamide* ***39***

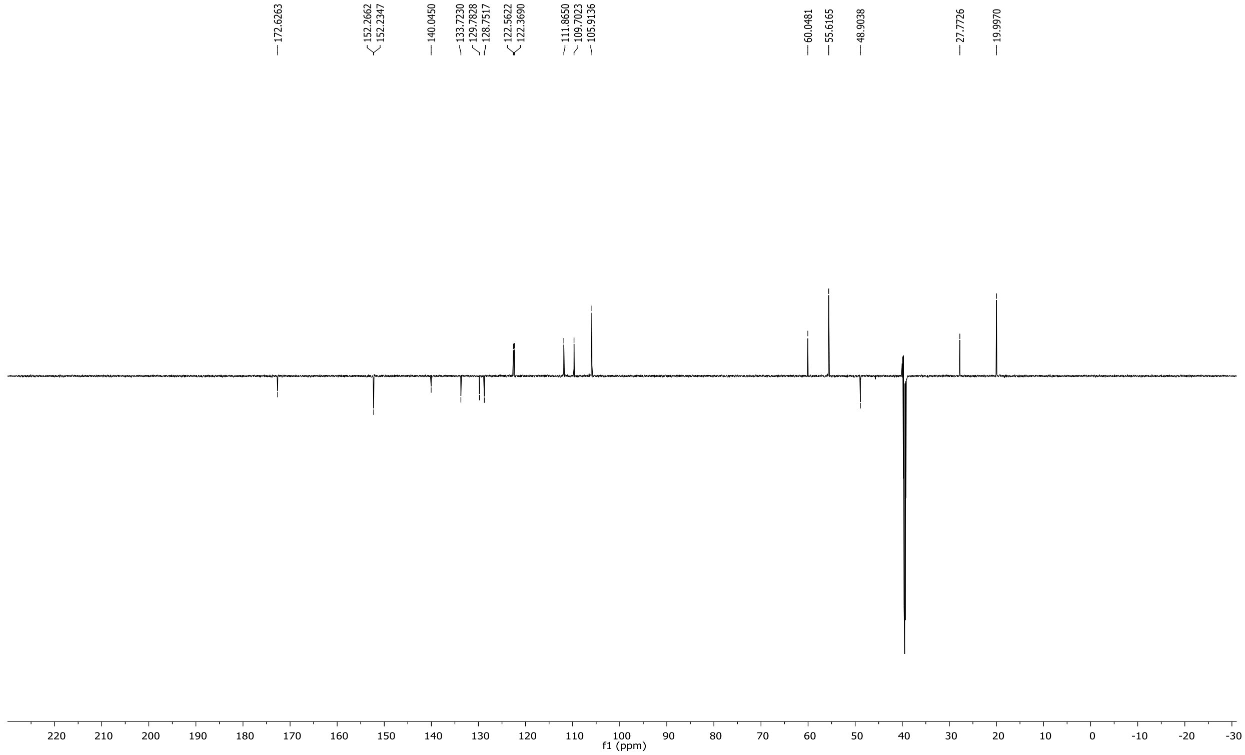

Figure S62. ^{13}C NMR spectrum (DMSO-d_6, 151 MHz) of *N-(1-isobutyl-1H-benzo[d]imidazol-2-yl)-3,4,5-trimethoxybenzamide **39***

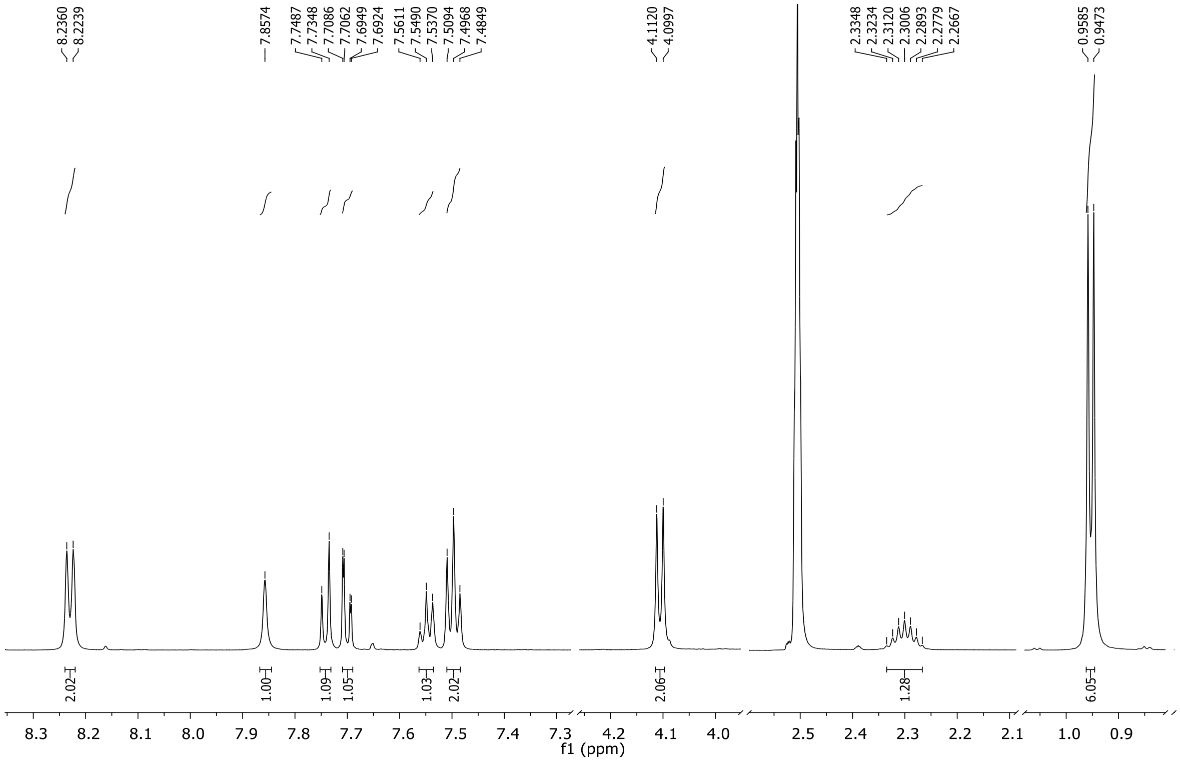

Figure S63. ^{1}H NMR spectrum (DMSO-d_6, 600 MHz) of *N-(6-cyano-1-isobutyl-1H-benzo[d]imidazol-2-yl)benzamide **40***

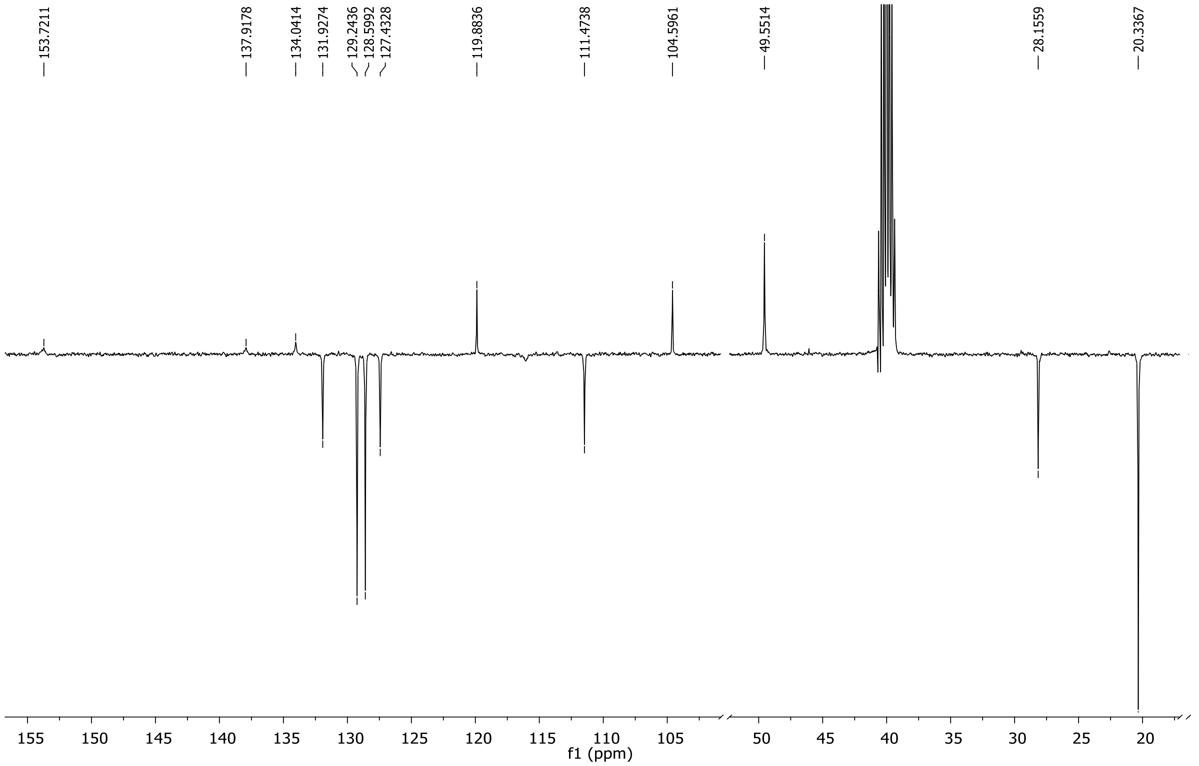

Figure S64. ^{13}C NMR spectrum (DMSO-d_6, 101 MHz) of *N-(6-cyano-1-isobutyl-1H-benzo[d]imidazol-2-yl)benzamide* ***40***

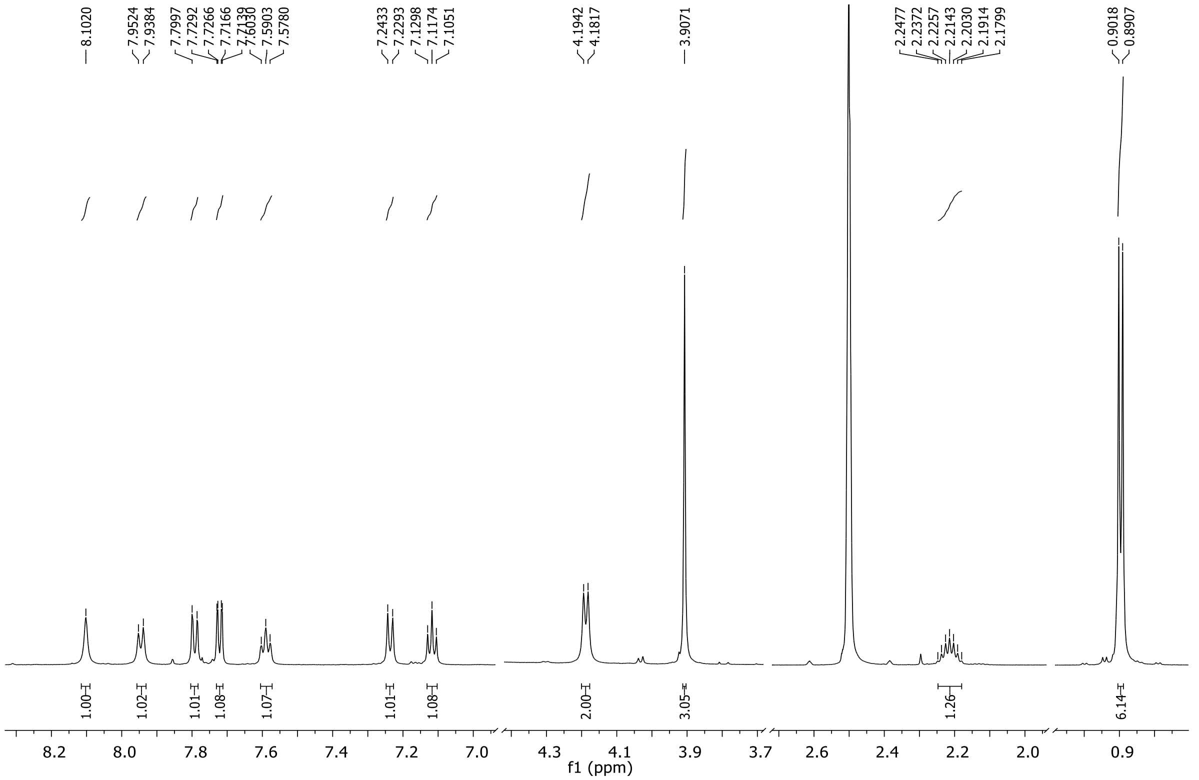

Figure S65. ^{1}H NMR spectrum (DMSO-d_6, 600 MHz) of *N-(6-cyano-1-isobutyl-1Hbenzo[d]imidazol-2-yl)-2-methoxybenzamide* ***41***

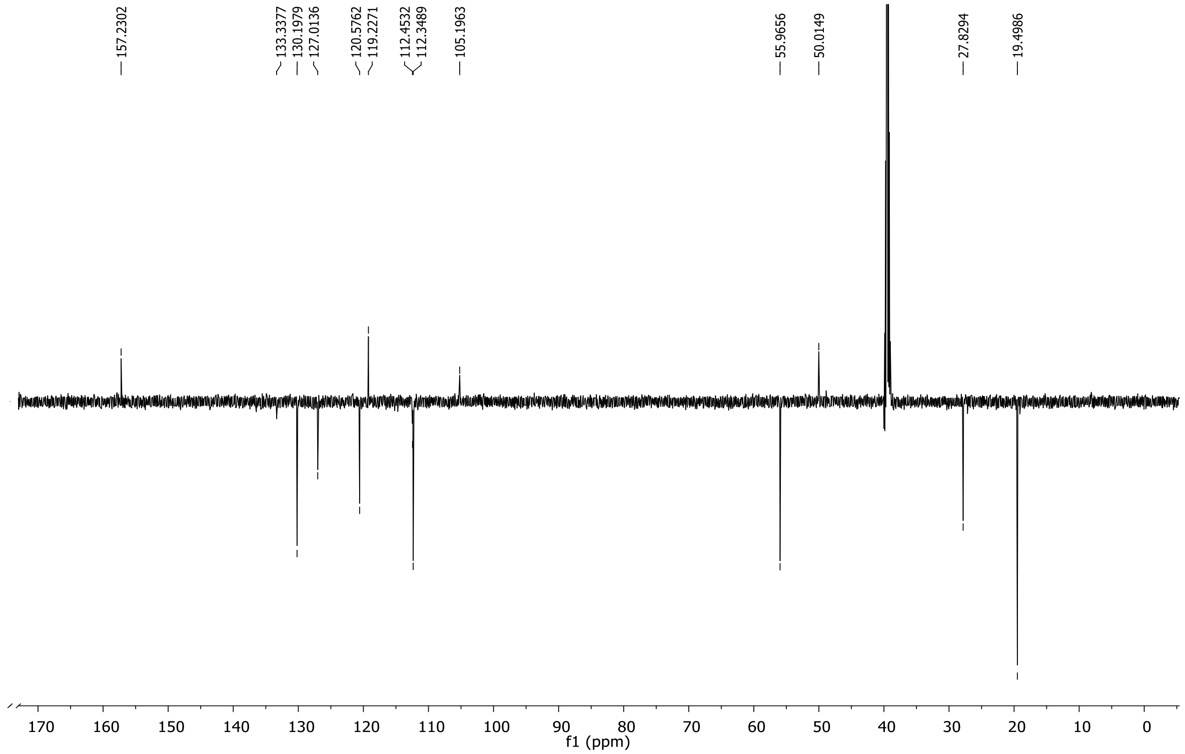

Figure S66. ^{13}C NMR spectrum (DMSO-d_6, 151 MHz) of *N-(6-cyano-1-isobutyl-1H-benzo[d]imidazol-2-yl)-2-methoxybenzamide* ***41***

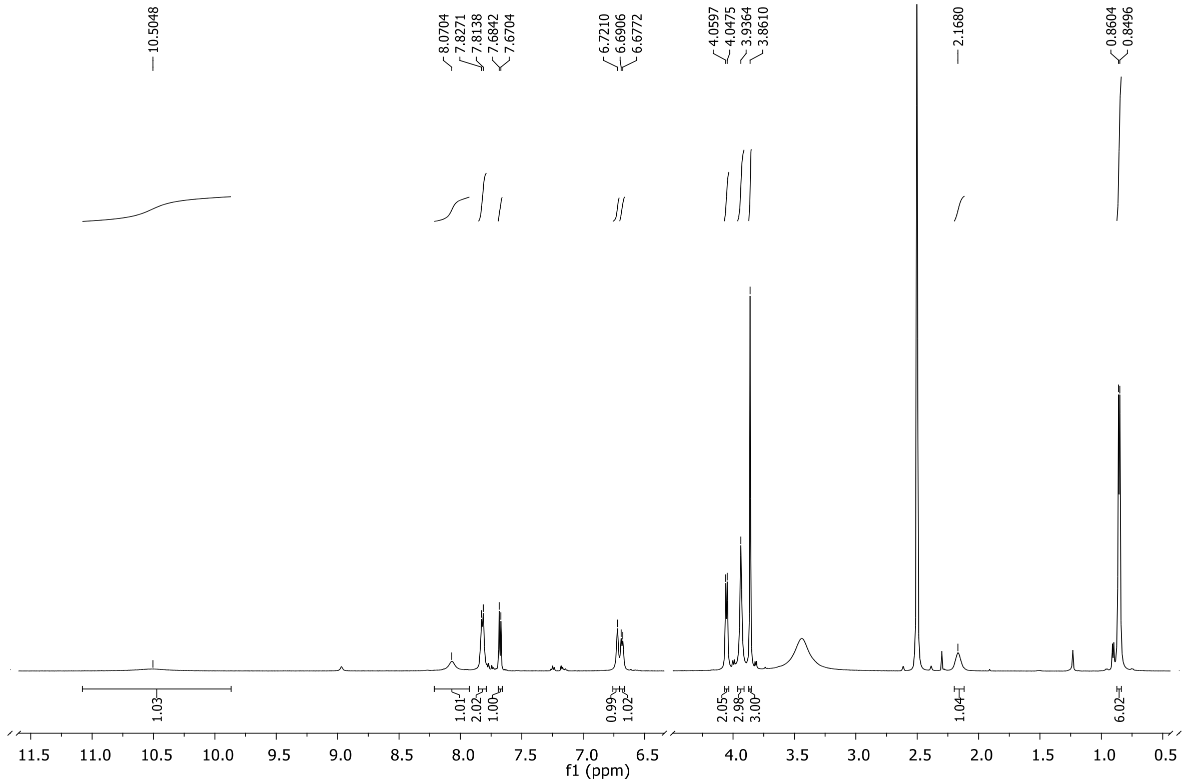

Figure S67. ^{1}H NMR spectrum (DMSO-d_6, 300 MHz) of *N-(6-cyano-1-isobutyl-1H-benzo[d]imidazol-2-yl)-2,4-dimethoxybenzamide* ***42***

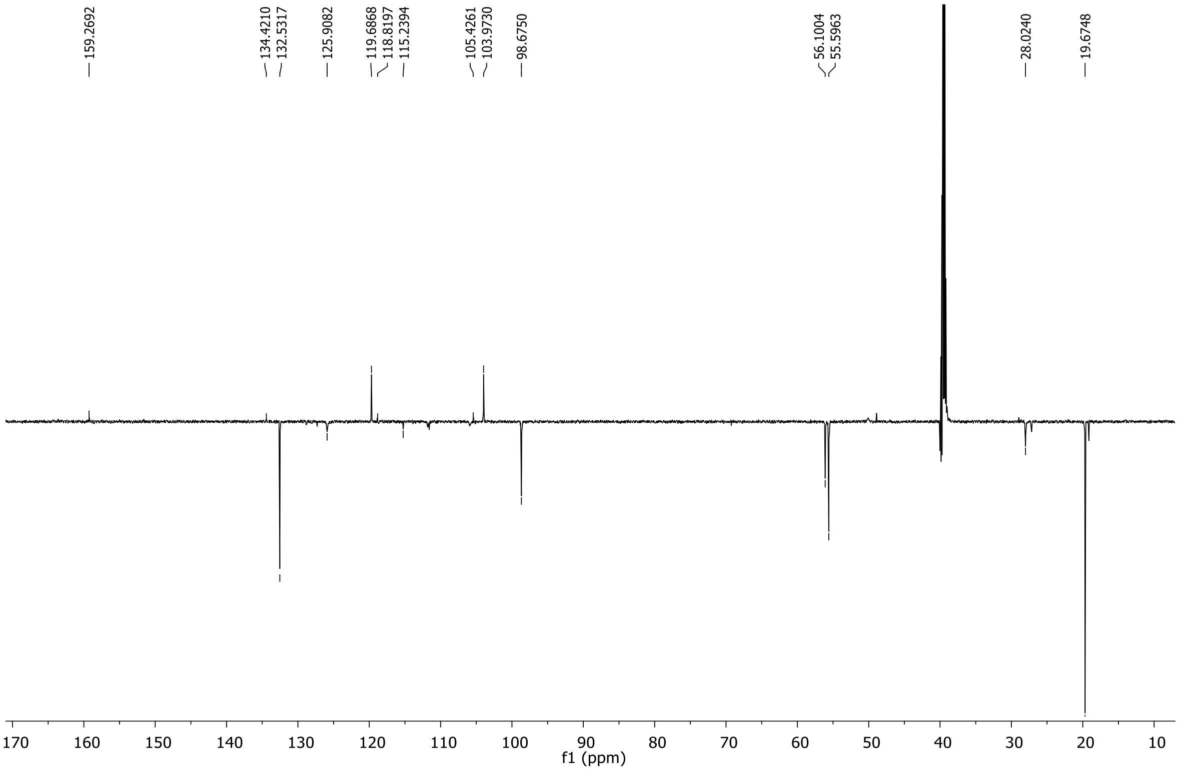

Figure S68. ^{13}C NMR spectrum (DMSO-d_6, 75 MHz) of *N-(6-cyano-1-isobutyl-1H-benzo[d]imidazol-2-yl)-2,4-dimethoxybenzamide* ***42***

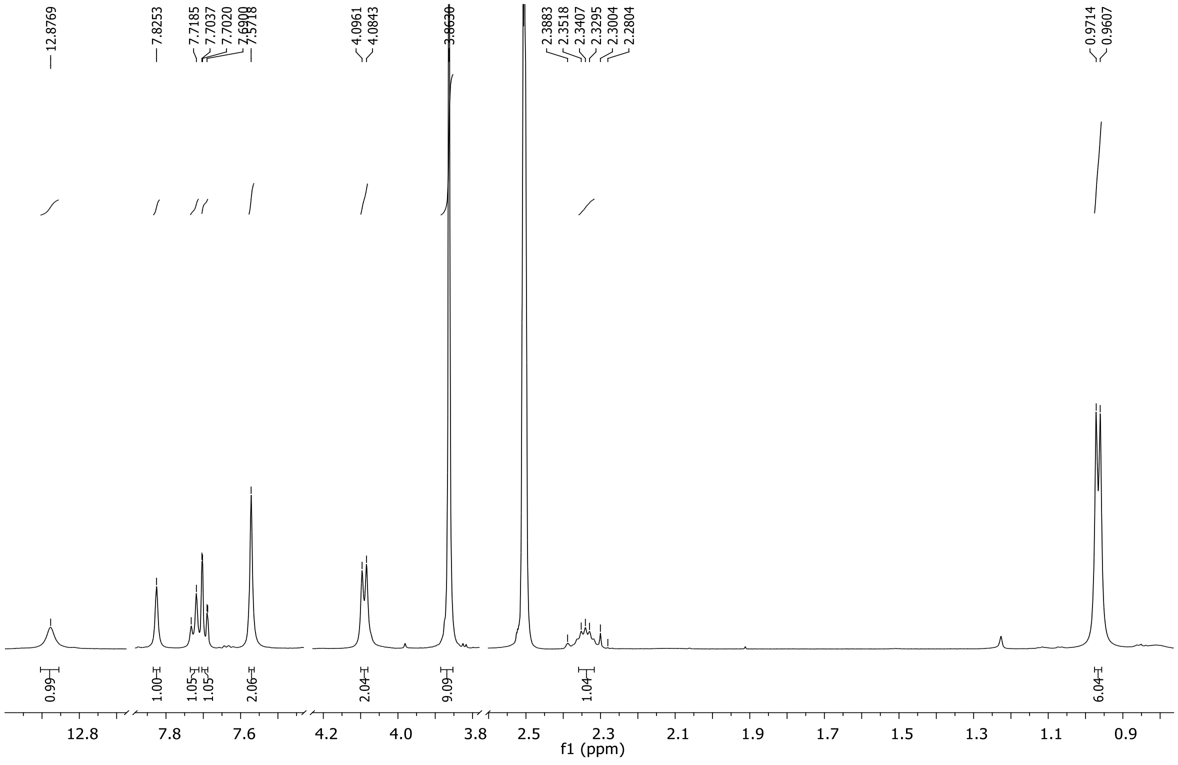

Figure S69. ^{1}H NMR spectrum (DMSO-d_6, 600 MHz) of *N-(6-cyano-1-isobutyl-1H-benzo[d]imidazol-2-yl)-3,4,5-trimethoxybenzamide* ***43***

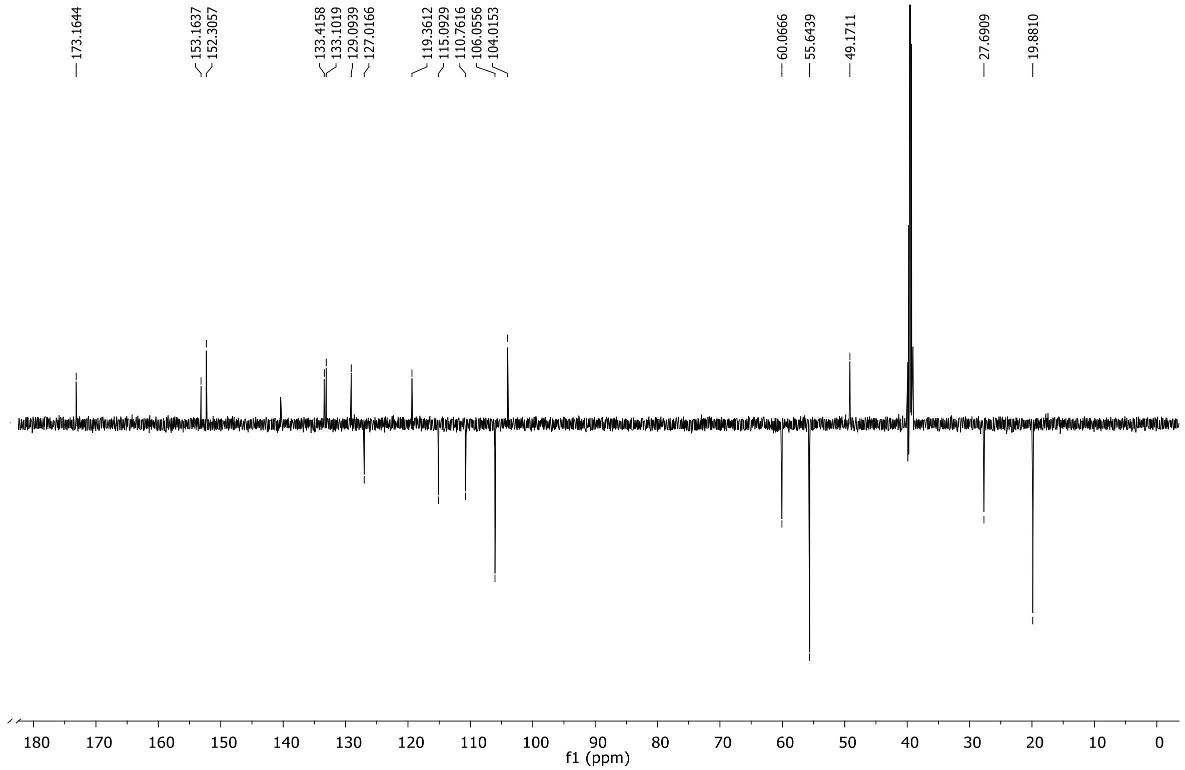

Figure S70. ^{13}C NMR spectrum (DMSO-d_6, 151 MHz) of *N-(6-cyano-1-isobutyl-1H-benzo[d]imidazol-2-yl)-3,4,5-trimethoxybenzamide* ***43***

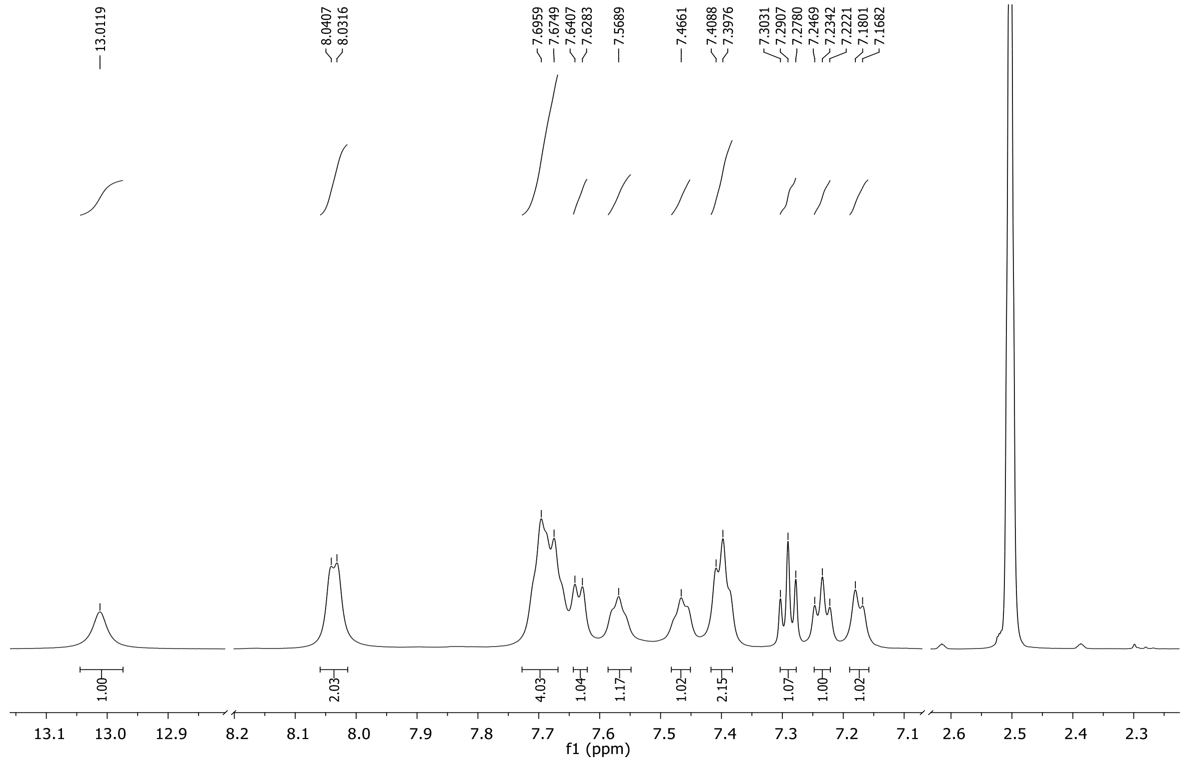

Figure S71. ^{1}H NMR spectrum (DMSO-d_6, 600 MHz) of *N-(1-phenyl-1H-benzo[d]imidazol-2-yl)benzamide* ***44***

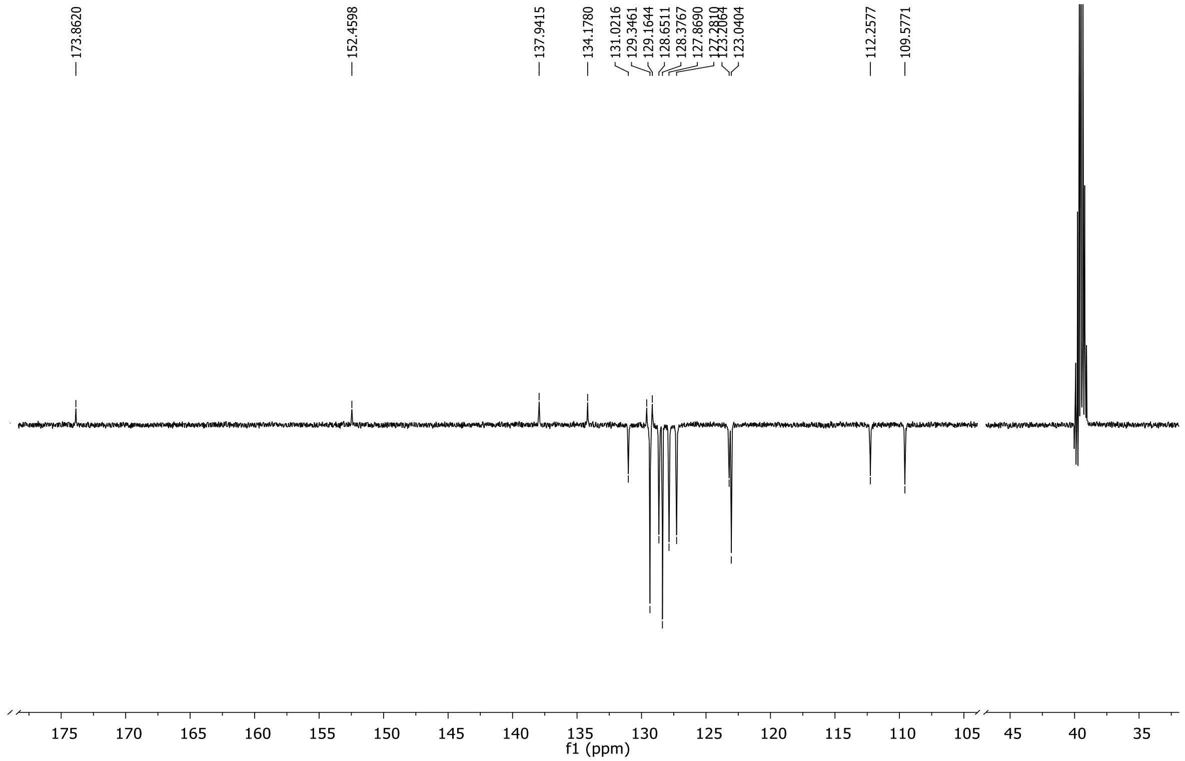

Figure S72. ^{13}C NMR spectrum (DMSO-d_6, 151 MHz) of *N-(1-phenyl-1H-benzo[d]imidazol-2-yl)benzamide **44***

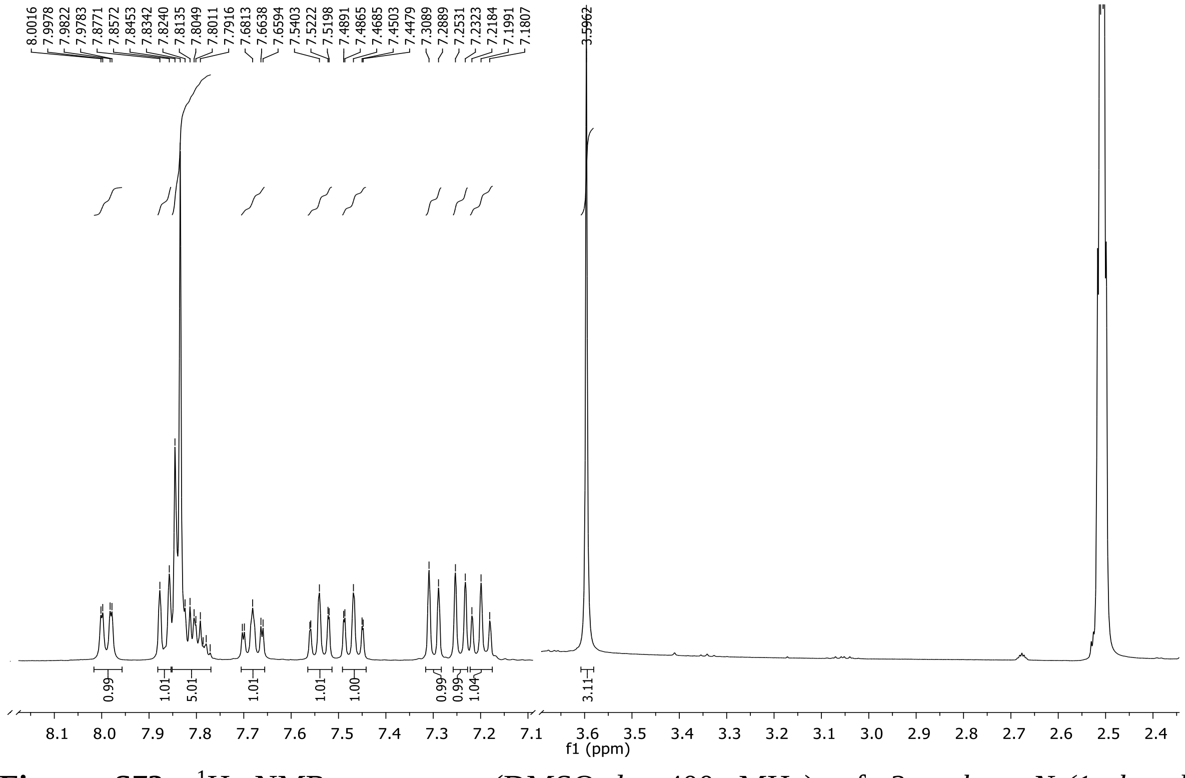

Figure S73. ^{1}H NMR spectrum (DMSO-d_6, 400 MHz) of *2-methoxy-N-(1-phenyl-1H-benzo[d]imidazol-2-yl)benzamide **45***

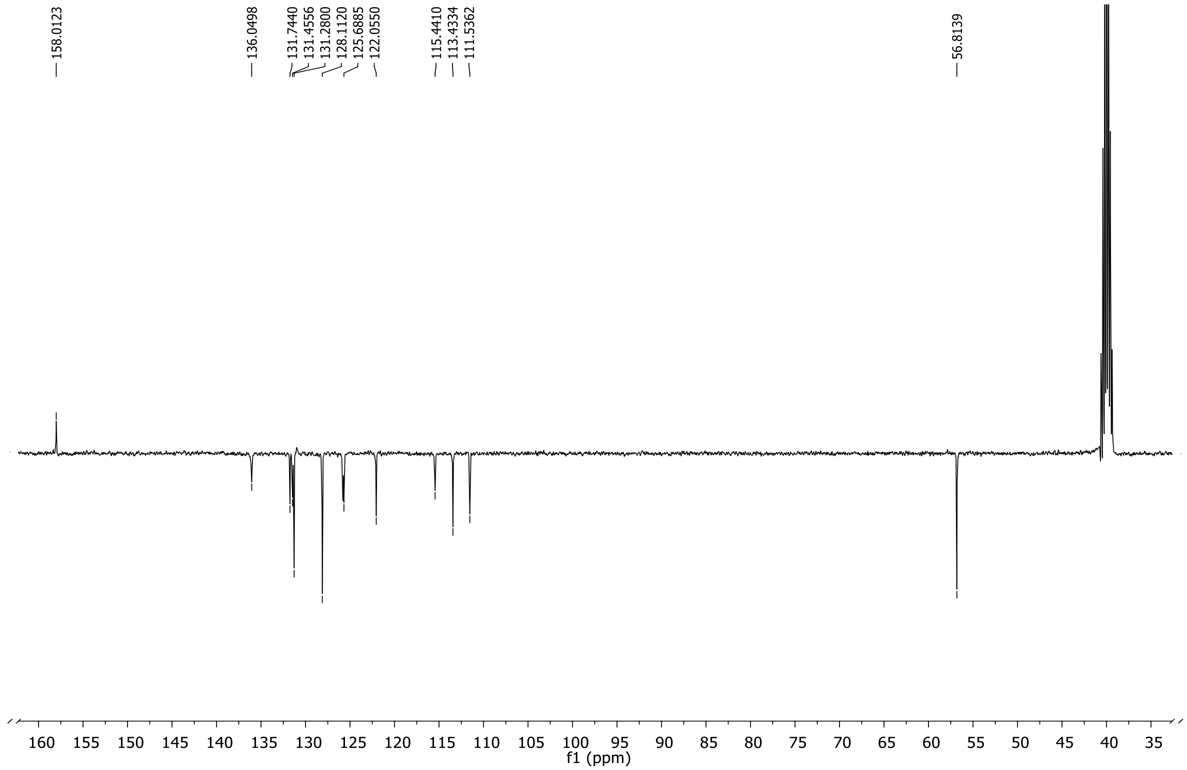

Figure S74. ^{13}C NMR spectrum (DMSO-d_6, 101 MHz) of *2-methoxy-N-(1-phenyl-1H-benzo[d]imidazol-2-yl)benzamide **45***

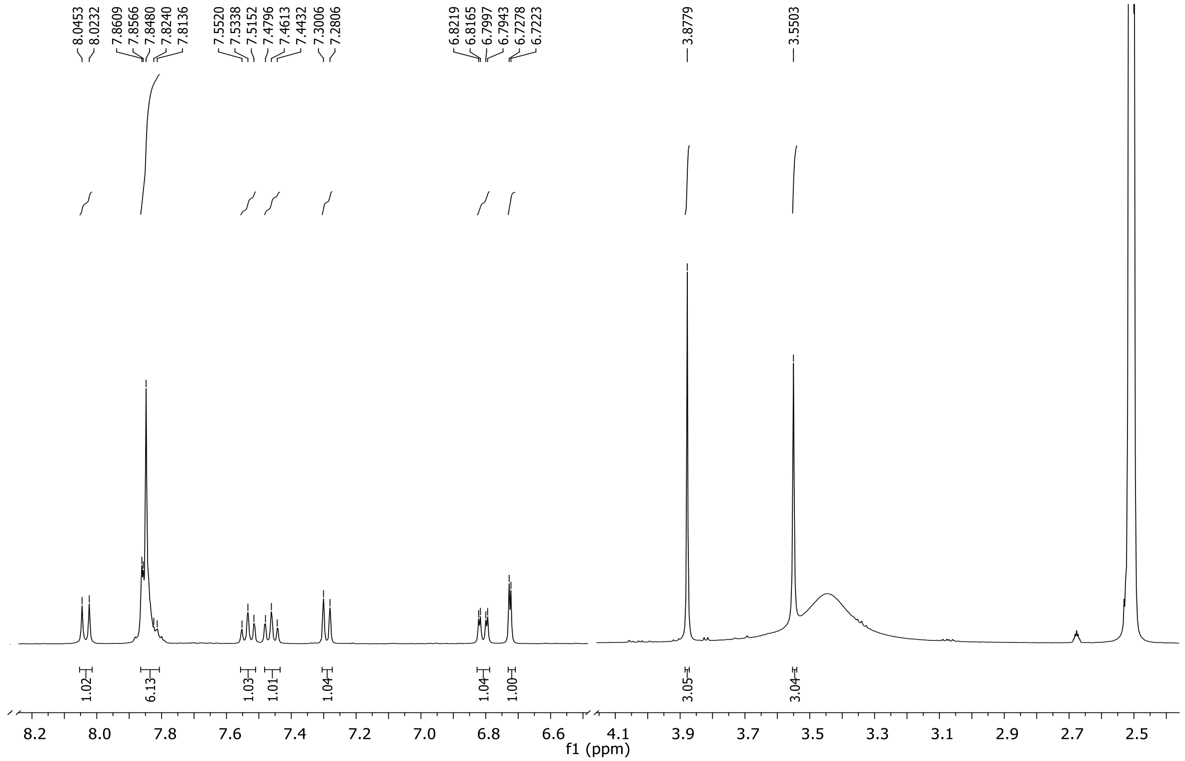

Figure S75. ^{1}H NMR spectrum (DMSO-d_6, 400 MHz) of *2,4-dimethoxy-N-(1-phenyl-1H-benzo[d]imidazol-2-yl)benzamide **46***

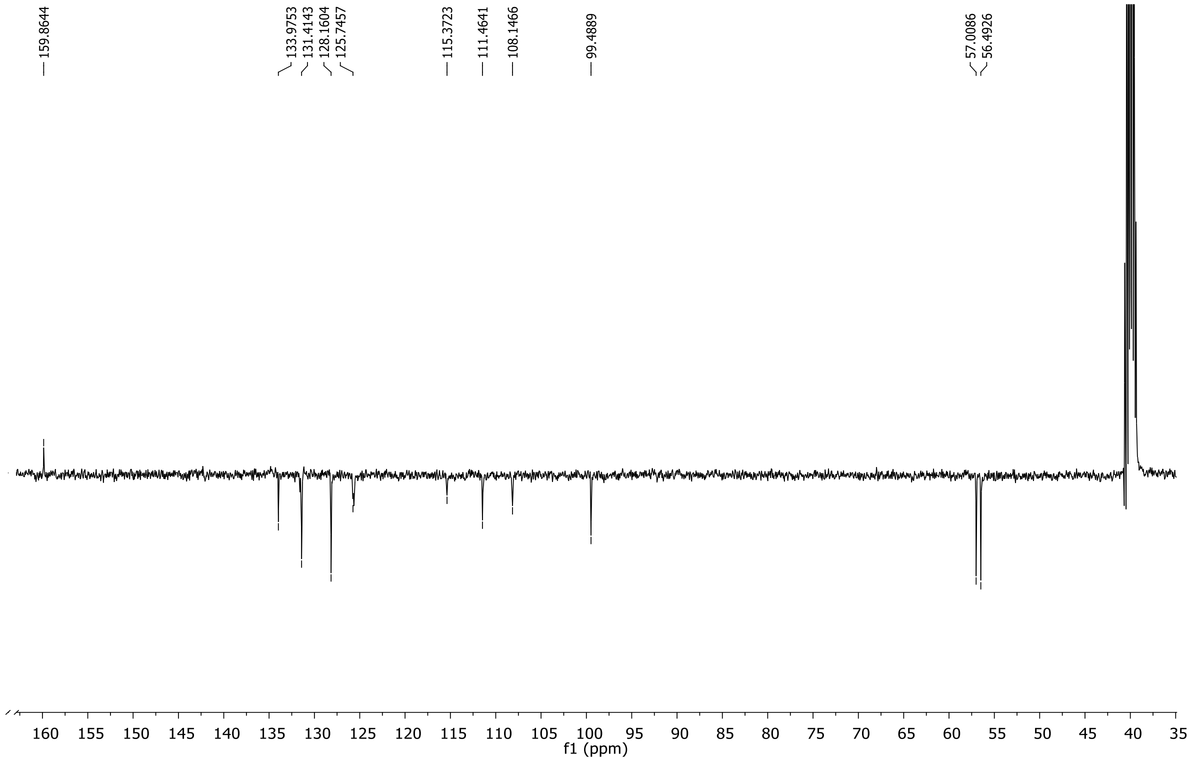

Figure S76. ^{13}C NMR spectrum (DMSO-d_6, 75 MHz) of *2,4-dimethoxy-N-(1-phenyl-1H-benzo[d]imidazol-2-yl)benzamide* ***46***

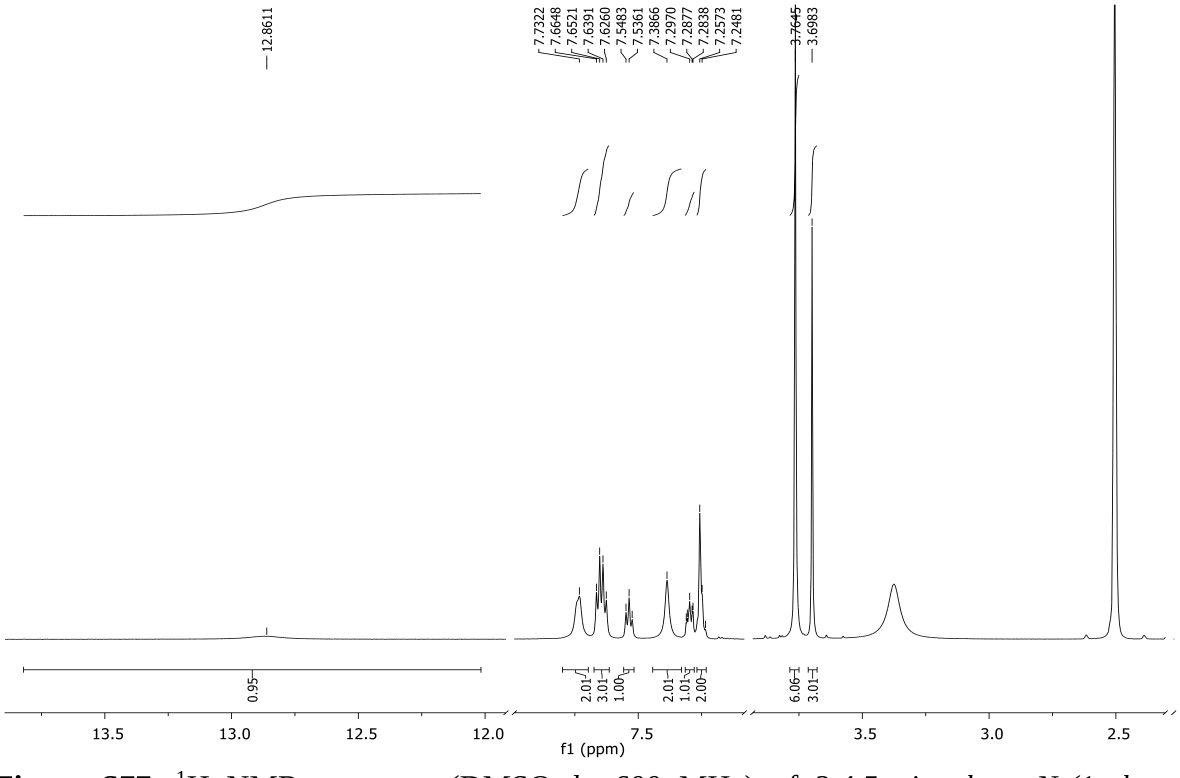

Figure S77. ^{1}H NMR spectrum (DMSO-d_6, 600 MHz) of *3,4,5-trimethoxy-N-(1-pheny-1H-benzo[d]imidazol-2-yl)benzamide* ***47***

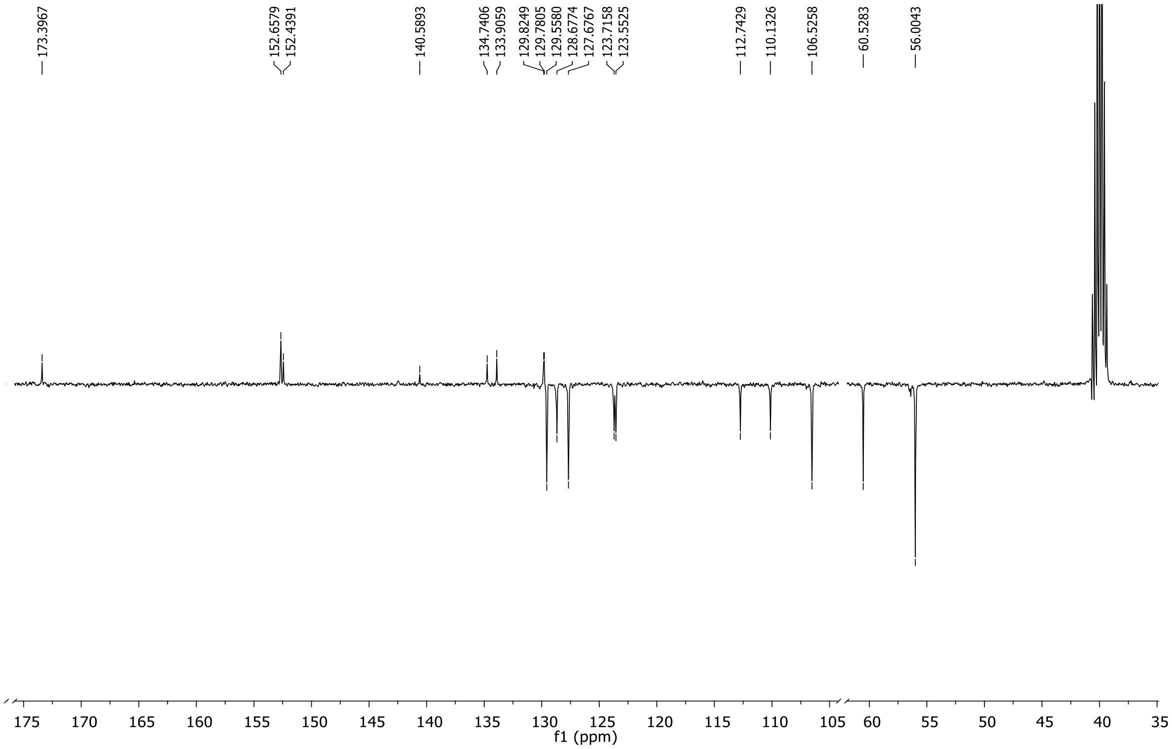

Figure S78. ^{13}C NMR spectrum (DMSO-d_6, 151 MHz) of *3,4,5-trimethoxy-N-(1-pheny-1H-benzo[d]imidazol-2-yl)benzamide **47***

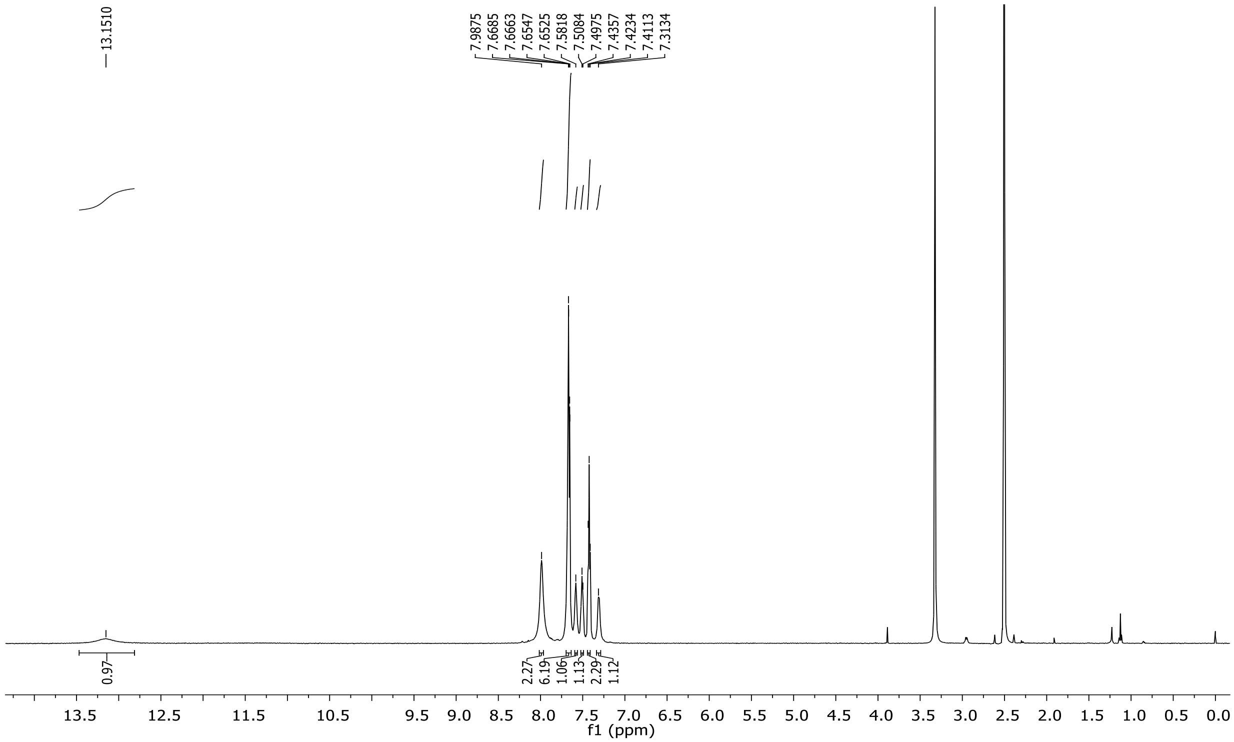

Figure S79. ^{1}H NMR spectrum (DMSO-d_6, 600 MHz) of *N-(6-cyano-1-phenyl-1H-benzo[d]imidazol-2-yl)benzamide **48***

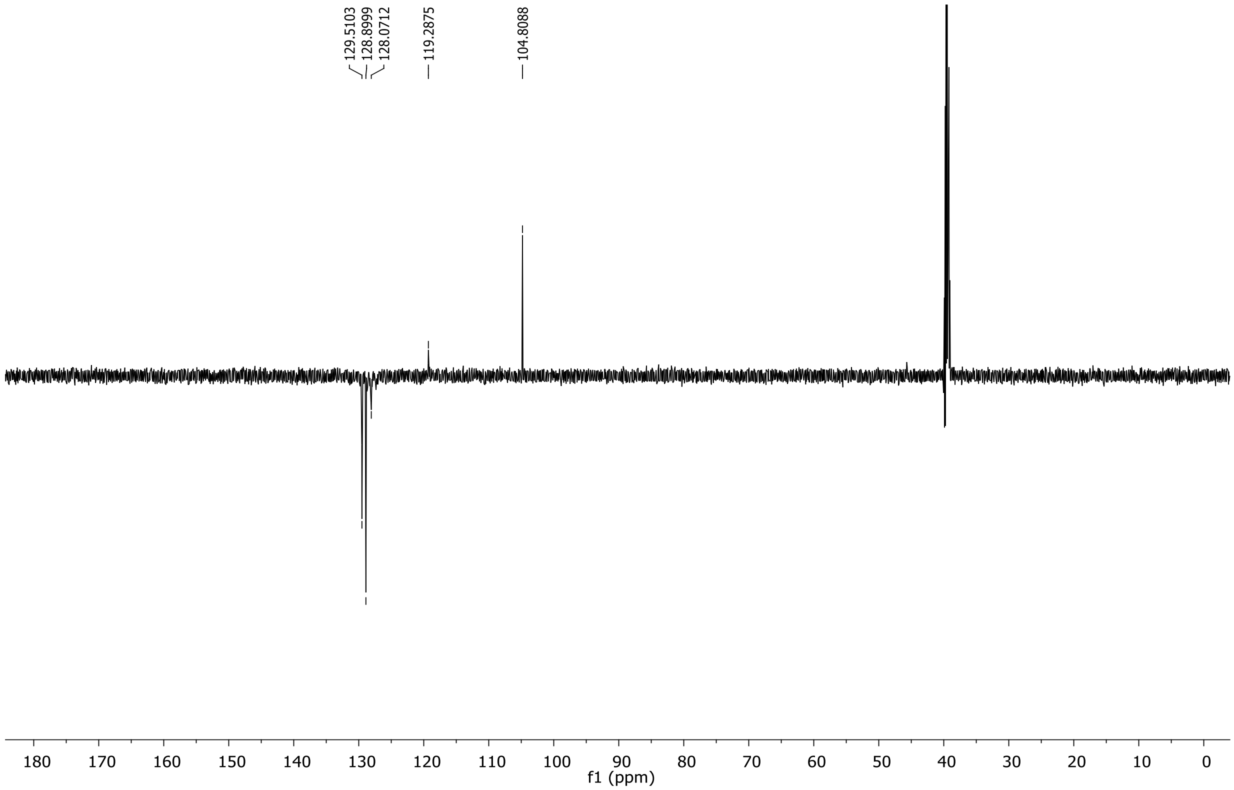

Figure S80. ^{13}C NMR spectrum (DMSO-d_6, 151 MHz) of *N-(6-cyano-1-phenyl-1H-benzo[d]imidazol-2-yl)benzamide* ***48***

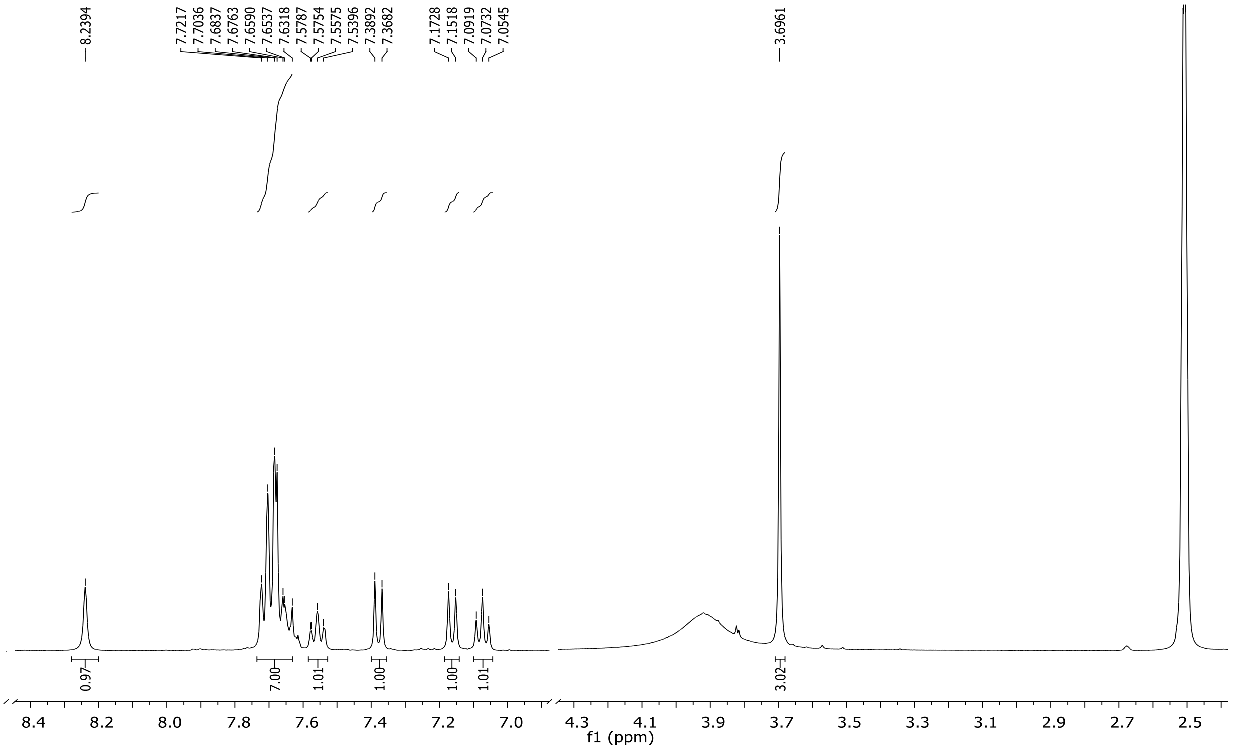

Figure S81. ^{1}H NMR spectrum (DMSO-d_6, 400 MHz) of *N-(6-cyano-1-phenyl-1H-benzo[d]imidazol-2-yl)-2-methoxybenzamide* ***49***

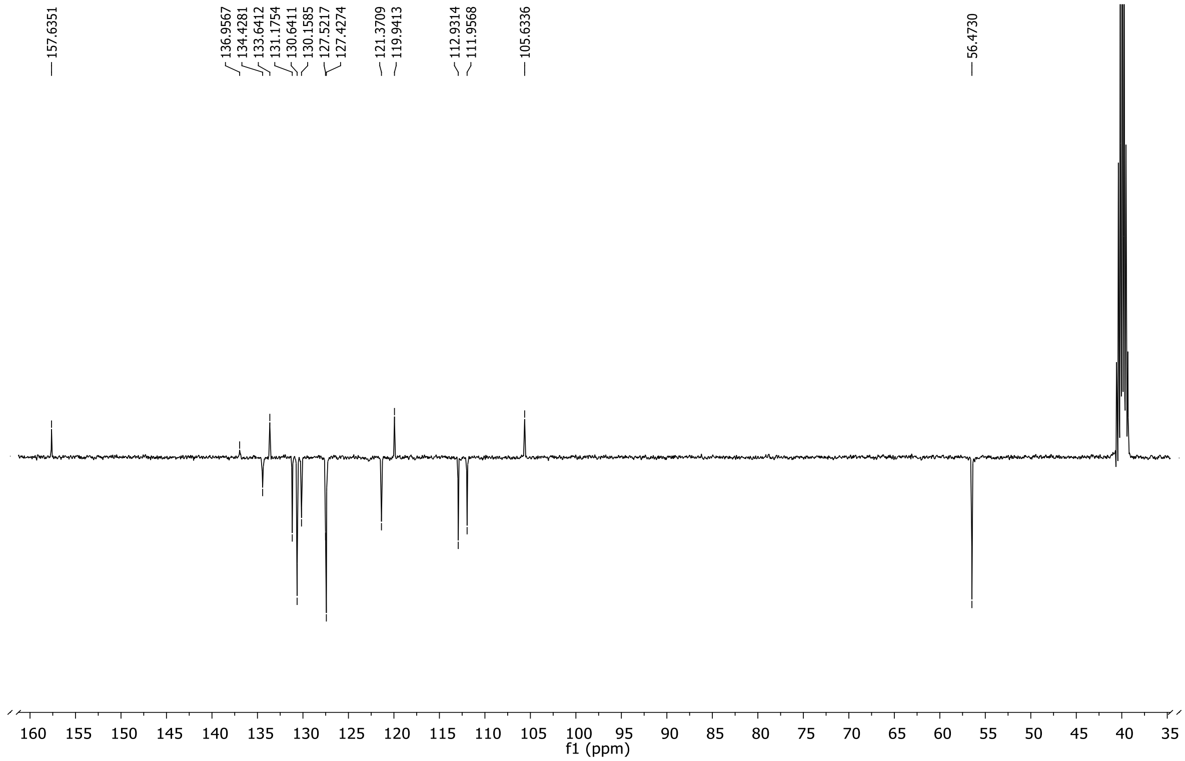

Figure S82. ^{13}C NMR spectrum (DMSO-d_6, 101 MHz) of *N-(6-cyano-1-phenyl-1H-benzo[d]imidazol-2-yl)-2-methoxybenzamide* ***49***

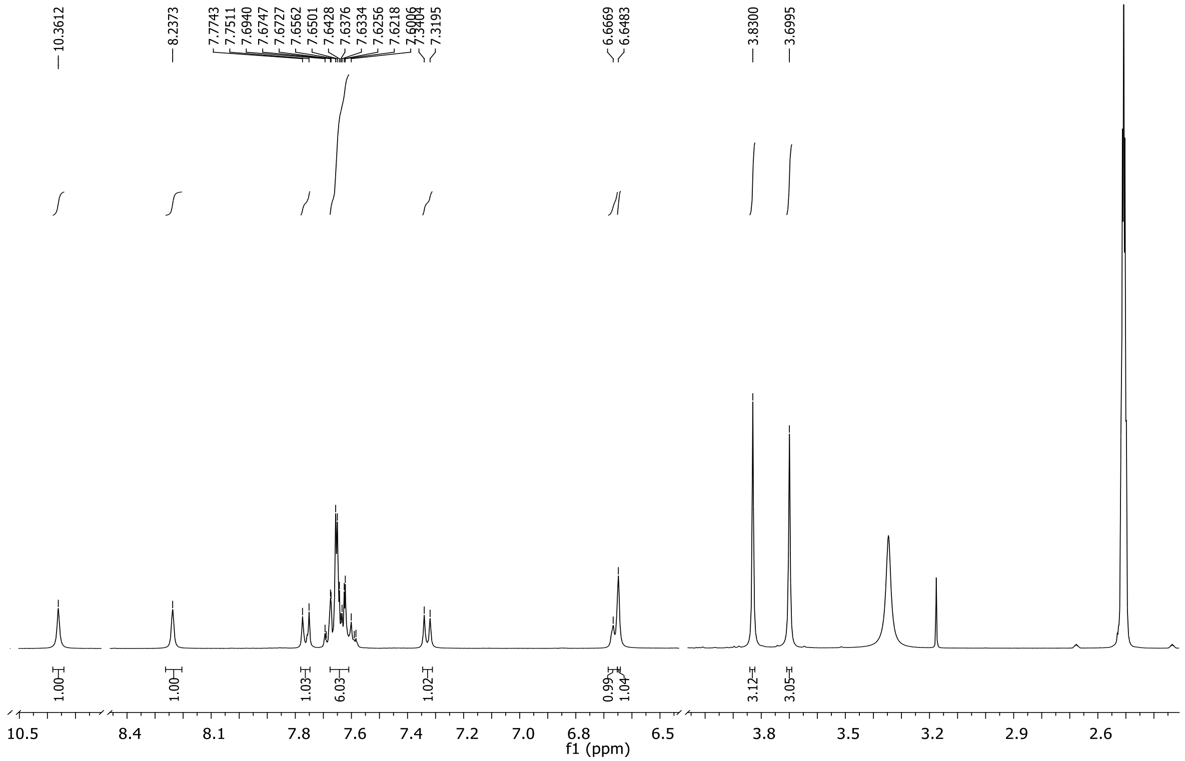

Figure S83. ^{1}H NMR spectrum (DMSO-d_6, 400 MHz) of *N-(6-cyano-1-phenyl-1H-benzo[d]imidazol-2-yl)-2,4-dimethoxybenzamide* ***50***

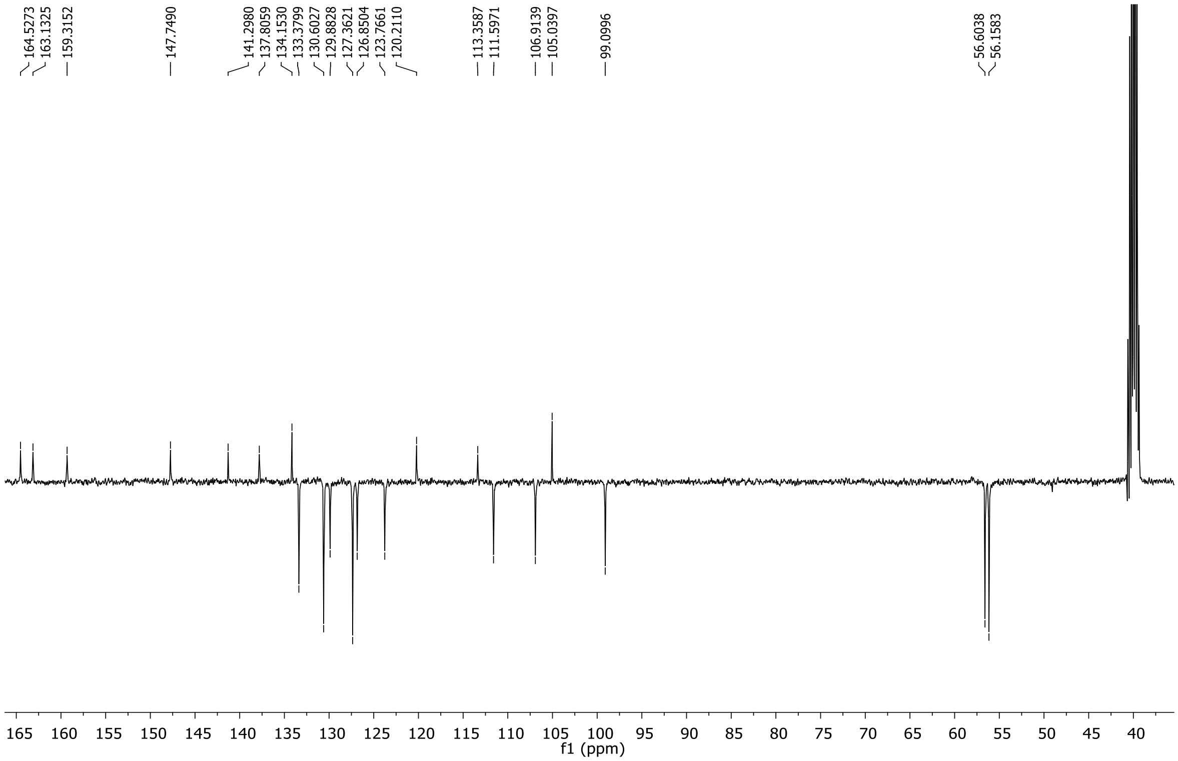

Figure S84. ^{13}C NMR spectrum (DMSO-d_6, 101 MHz) of *N-(6-cyano-1-phenyl-1H-benzo[d]imidazol-2-yl)-2,4-dimethoxybenzamide **50***

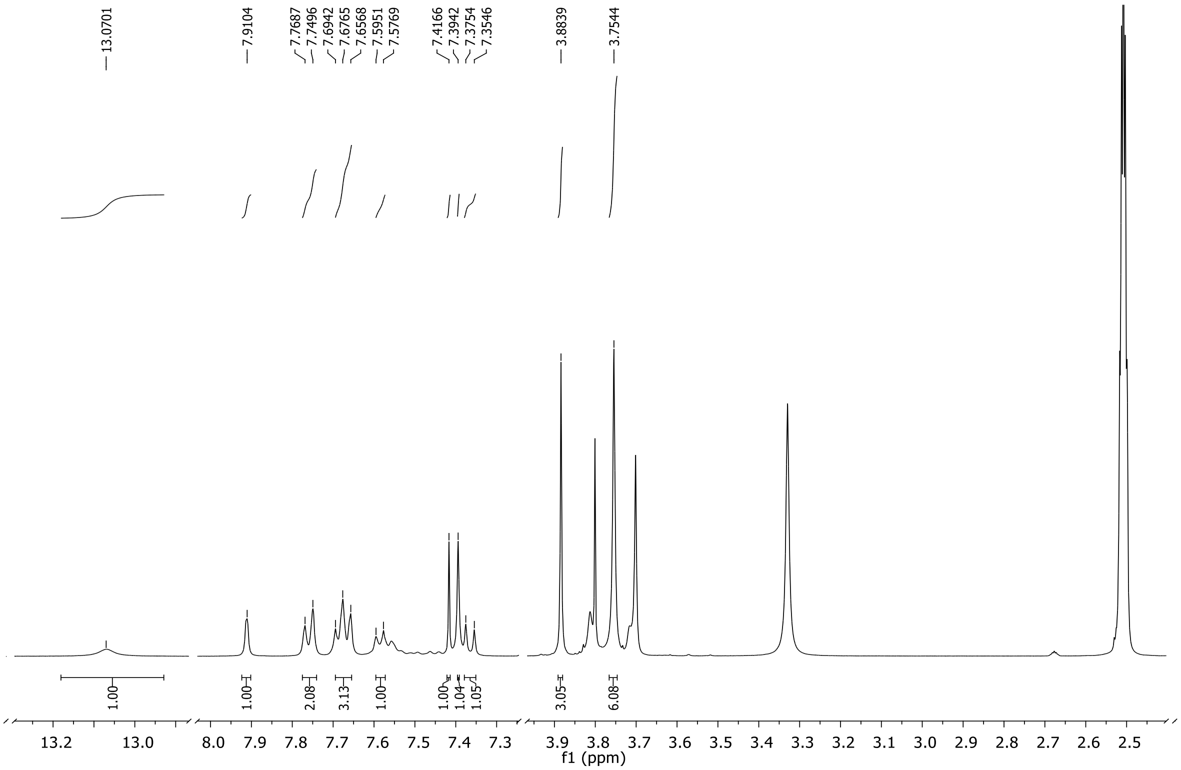

Figure S85. ^{1}H NMR spectrum (DMSO-d_6, 400 MHz) of *N-(6-cyano-1-phenyl-1H-benzo[d]imidazol-2-yl)-3,4,5-trimethoxybenzamide **51***

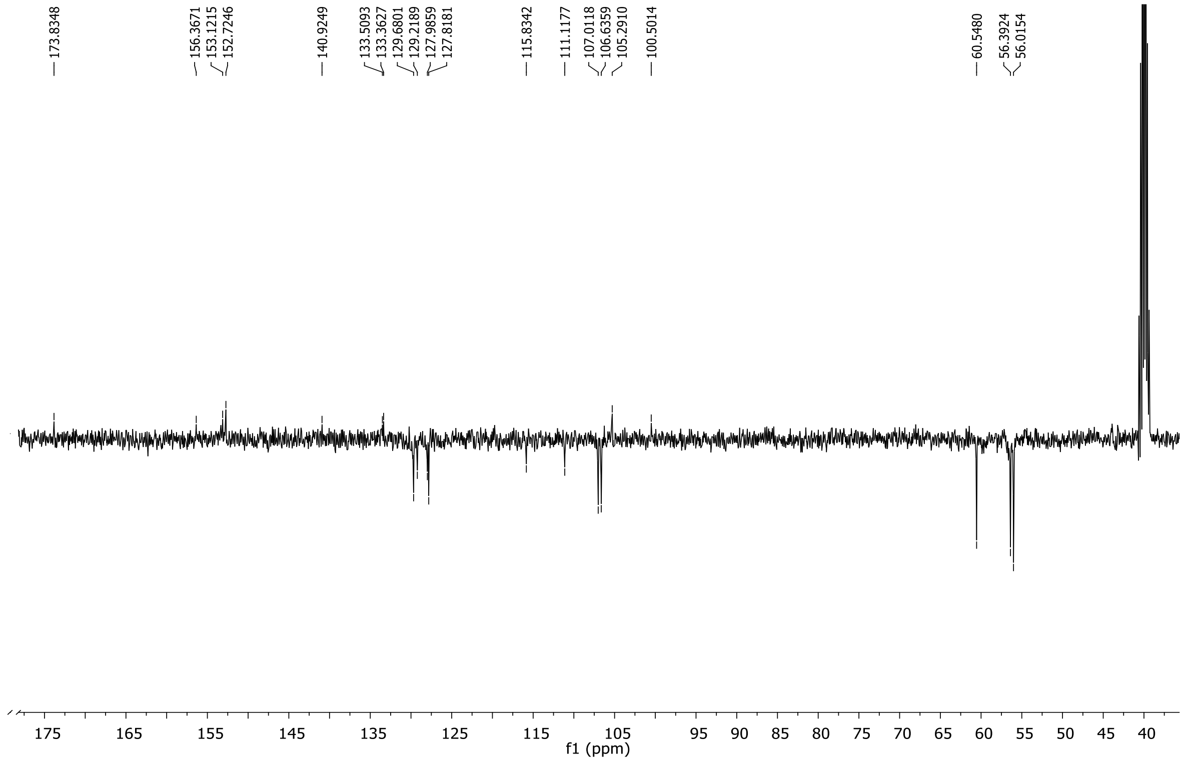

Figure S86. ^{13}C NMR spectrum (DMSO-d_6, 101 MHz) of *N-(6-cyano-1-phenyl-1H-benzo[d]imidazol-2-yl)-3,4,5-trimethoxybenzamide* ***51***
